# A Brief Home-Based Parenting Intervention to Reduce Behavior Problems in Young Children: A Pragmatic Randomized Clinical Trial

**DOI:** 10.1001/jamapediatrics.2020.6834

**Published:** 2021-03-15

**Authors:** Christine O’Farrelly, Hilary Watt, Daphne Babalis, Marian J. Bakermans-Kranenburg, Beth Barker, Sarah Byford, Poushali Ganguli, Ellen Grimas, Jane Iles, Holly Mattock, Julia McGinley, Charlotte Phillips, Rachael Ryan, Stephen Scott, Jessica Smith, Alan Stein, Eloise Stevens, Marinus H. van IJzendoorn, Jane Warwick, Paul G. Ramchandani

**Affiliations:** 1 Division of Psychiatry, Imperial College London, London, United Kingdom; 2 Play in Education, Development, and Learning (PEDAL) Research Centre, Faculty of Education, University of Cambridge, Cambridge, United Kingdom; 3 School of Public Health, Imperial College London, London, United Kingdom; 4 Imperial Clinical Trials Unit, Imperial College London, London, United Kingdom; 5 Clinical Child and Family Studies, Faculty of Behavioural and Movement Sciences, Vrije Universiteit Amsterdam, Amsterdam, the Netherlands; 6 Institute of Psychology, Psychiatry, and Neuroscience, King’s College London, London, United Kingdom; 7 School of Psychology, University of Surrey, Guildford, United Kingdom; 8 Parent Supporter Service, Netmums, London, United Kingdom; 9 Department of Psychiatry, University of Oxford, Oxford, United Kingdom; 10 African Health Research Institute, Durban, South Africa; 11 Department of Psychology, Education, and Child Studies, Erasmus University Rotterdam, Rotterdam, the Netherlands; 12 Warwick Clinical Trials Unit, University of Warwick, Coventry, United Kingdom

## Abstract

**Importance:**

Behavior problems are one of the most common mental health disorders in childhood and can undermine children’s health, education, and employment outcomes into adulthood. There are few effective interventions for early childhood.

**Objective:**

To test the clinical effectiveness of a brief parenting intervention, the Video-feedback Intervention to promote Positive Parenting and Sensitive Discipline (VIPP-SD), in reducing behavior problems in children aged 12 to 36 months.

**Design, Setting, and Participants:**

The Healthy Start, Happy Start study was a 2-group, parallel-group, researcher-blind, multisite randomized clinical trial conducted via health visiting services in 6 National Health Service trusts in England. Baseline and 5-month follow-up data were collected between July 30, 2015, and April 27, 2018. Of 818 eligible families, 227 declined to participate, and 300 were randomized into the trial. Target participants were caregivers of children who scored in the top 20% for behavior problems on the Strengths and Difficulties Questionnaire. Participants were randomly allocated on a 1:1 basis to receive either VIPP-SD (n = 151) or usual care (n = 149), stratified by site and number of participating caregivers. Analysis was performed on an intention-to-treat basis. Statistical analysis was performed from September 5, 2019, to January 17, 2020.

**Interventions:**

All families continued to access usual care. Families allocated to VIPP-SD were offered 6 home-based video-feedback sessions of 1 to 2 hours’ duration every 2 weeks.

**Main Outcomes and Measures:**

The primary outcome was the score on an early childhood version of the Preschool Parental Account of Children’s Symptoms, a semistructured interview of behavior symptoms, at 5 months after randomization. Secondary outcomes included caregiver-reported behavior problems on the Child Behavior Checklist and the Strengths and Difficulties Questionnaire.

**Results:**

Among 300 participating children (163 boys [54%]; mean [SD] age, 23.0 [6.7] months), primary outcome data were available for 140 of 151 VIPP-SD participants (93%) and 146 of 149 usual care participants (98%). There was a mean difference in the total Preschool Parental Account of Children’s Symptoms score of 2.03 (95% CI, 0.06-4.01; *P* = .04; Cohen *d* = 0.20 [95% CI, 0.01-0.40]) between trial groups, with fewer behavior problems in the VIPP-SD group, particularly conduct symptoms (mean difference, 1.61 [95% CI, 0.44-2.78]; *P* = .007; *d* = 0.30 [95% CI, 0.08-0.51]). Other child behavior outcomes showed similar evidence favoring VIPP-SD. No treatment or trial-related adverse events were reported.

**Conclusions and Relevance:**

This study found that VIPP-SD was effective in reducing symptoms of early behavior problems in young children when delivered in a routine health service context.

**Trial Registration:**

isrctn.org Identifier: ISRCTN58327365

## Key Points

**Question:**

Does a brief video-feedback parenting intervention delivered in a routine health context improve behavior outcomes for at-risk children aged 12 to 36 months?

**Findings:**

This randomized clinical trial that included 300 children and their caregivers found lower levels of behavior problems among families allocated to receive the intervention vs those who received usual care. In particular, conduct problems decreased.

**Meaning:**

This brief parenting intervention can benefit the mental health of very young children and can be delivered by frontline staff in a routine community health service setting.

## Teaser

This randomized clinical trial tests the clinical effectiveness of a brief video feedback–based parenting intervention in reducing behavior problems in children aged 12 to 36 months.

## Introduction

Behavior problems are one of the most common mental health disorders in childhood, affecting 5% to 10% of children.^1,2^ Children with enduring behavior problems are at risk of poorer health, social, and educational outcomes across the life course, causing distress to families and generating large costs for society.^2,3,4,5,6^

A key risk factor for behavior problems is the parental care that children receive.^7^ Parenting interventions are effective in reducing behavior problems; however, most programs target preschool-aged and school-aged children.^8,9^ Intervening earlier in childhood could be more effective from a clinical, economic, and educational perspective because there is greater opportunity to intercept psychopathologic symptoms before they become embedded.^10^ However, to our knowledge, relatively few interventions have targeted behavior problems at their onset in 1- and 2-year-old children, particularly in routine health services.^8,11^ Notable exceptions support the feasibility and efficacy of parent training for families of infants in the home^12,13^ and the effectiveness of group parent training for 2- to 4-year-old children in health care settings.^14^ There is also some support for the long-term benefits of early interventions on behavior outcomes.^13,15^

This study aimed to test the effectiveness of a brief parenting intervention in preventing behavior problems in at-risk children as compared with usual care (UC). Video-feedback Intervention to promote Positive Parenting and Sensitive Discipline (VIPP-SD) targets parents’ sensitivity and sensitive discipline and is suitable for children as young as 12 months. It has an established evidence base from 12 randomized clinical trials (RCTs) for caregivers’ sensitivity (combined effect size, Cohen *d* = 0.47) and children’s behavior problems (7 trials; *d* = 0.26).^16^ However, VIPP-SD has yet to be tested in a routine health service, to our knowledge.

The primary hypothesis was that, among children aged 12 to 36 months with high levels of behavior problems, adding a brief video-feedback intervention to UC would reduce behavior problems, measured by a clinical interview at 5 months after randomization. We also hypothesized that the intervention would reduce behavior problems as measured by caregiver-reported questionnaires.

## Methods

### Study Design, Setting, and Participants

Healthy Start, Happy Start was a 2-group, parallel-group, researcher-blind, multisite RCT.^17^ The protocol was approved by a National Health Service (NHS) Ethics Committee and is available with the statistical analysis plan in Supplement 1. Parents or caregivers provided written informed consent. This study followed the Consolidated Standards of Reporting Trials (CONSORT) reporting guideline.

Recruitment via 6 NHS trusts in the UK involved a screening stage followed by a trial stage. Recruitment to the screening stage was through face-to-face or postal contacts in health visiting services, supplemented by advertisements in other clinical and community services and online outlets. Screening identified families whose children scored in the top 20% for behavior problems (≥8 on the externalizing subscale) on the Strengths and Difficulties Questionnaire (SDQ)^18^ based on the 2- to 4-year-old norming sample (R. Goodman, PhD, email, May 7, 2015). Caregivers whose children met eligibility owing to their SDQ score were contacted via telephone to determine the family’s full eligibility and interest in the trial. One or 2 caregivers could participate.

Families were included if the parent(s) or caregiver(s) were older than 18 years and provided written informed consent and the child was aged 12 to 36 months and scored in the top 20% for externalizing behaviors on the SDQ. Families were excluded if the child or parent had a sensory impairment, learning disability, or language limitation that precluded their participation; if a sibling was already participating in the study; or if the family was participating in another closely related research trial, receiving an individualized video-feedback intervention, and/or participating in active court proceedings. Recruitment took place between July 30, 2015, and July 26, 2017. Posttreatment assessments took place between December 9, 2015, and April 27, 2018.

### Randomization

Research assistants enrolled participants on the study electronic data capture system. Participants were randomly allocated on a 1:1 basis to receive VIPP-SD or UC, stratified by recruitment site and willingness and availability of caregivers (1 vs 2) to participate. The randomization list, prepared by an independent statistician using varying block sizes (of 2, 4, and 6), was uploaded to the electronic data capture system, and participants were allocated to the next available treatment code in the appropriate list. The allocation sequence was not accessible to the trial manager, chief investigator, or clinical supervisors. Research assistants who conducted assessments were blinded to treatment allocation, and clinical staff who delivered the intervention did not conduct assessments.

### Intervention Description

Families in the VIPP-SD group were offered 6 home-based sessions of 1- to 2-hour duration every 2 weeks by a trained health professional. Video-feedback Intervention to promote Positive Parenting and Sensitive Discipline is a manualized intervention based on attachment and social learning theories.^16,19^ During 4 core and 2 booster sessions, the intervener films parents interacting with their child (approximately 10 minutes) during play-based and challenging interactions and then provides focused feedback on the filmed interactions from the previous visit.^20^ Treatment fidelity was monitored through regular clinical supervision, and visits were audio recorded to allow a random sample (10%) to be rated for fidelity.

Interveners were trained health professionals, predominantly public health nurses and nursery nurses, as well as a small number of professionals from therapy, psychology, and psychiatry backgrounds. Interveners completed 4 days of VIPP-SD training and 3 supervised practice sessions. Participants were allocated to the next available local intervener.

### Usual Care

Participants in both groups continued to receive UC, which was typically minimal (there are no standard care pathways in the NHS for early-onset behavior problems). Some participants received support and advice from a health visitor or general practitioner, referral to early intervention mental health services, or parenting support.

### Outcomes

Assessments were made at baseline (prerandomization) and 5 months after randomization (posttreatment; primary end point) by trained researchers in participants’ homes or children’s centers. The primary outcome was severity of behavior problems, assessed using a modified early childhood version of the Preschool Parental Account of Children’s Symptoms (PPACS).^21^ The PPACS is a semistructured researcher-led interview administered to a parent or caregiver. Interviews are the criterion standard outcome measure as they provide a more complete picture of children’s symptoms than is possible from questionnaires.^22,23^ To determine scores, the primary caregiver provides detailed examples of the child’s typical behavior over the last week and indicates how representative the behavior is of the last 4 months. A trained interviewer then rates the severity and frequency of the symptoms based on their professional judgment and written thresholds. The measure comprises 2 subscales: conduct problems and attention-deficit/hyperactivity disorder or hyperkinesis. The PPACS has good psychometric properties and has been used in previous RCTs.^21,24,25,26,27^ Interviews were recorded, and 10% (30 of 300) were randomly selected for double scoring at each time point; high reliability was observed (intraclass correlations, 0.93-0.97). The PPACS scores at 2 years after randomization were included as a secondary outcome and will be reported later.

Two other parent-reported measures of child behavior were used: the Child Behavior Checklist (CBCL)^28^ and the SDQ.^18^ The widely used and psychometrically robust CBCL provides total, internalizing, and externalizing scores. The externalizing scale comprises syndrome scores for attention and aggression problems. The SDQ is a brief and widely used measure that yields a total difficulties score and scores for emotional difficulties, peer problems, conduct problems, hyperactivity, and prosocial behavior. The conduct problems and hyperactivity scales can be combined to yield an externalizing subscale.

Other secondary outcomes were parent-reported parental discipline (the Parenting Scale), mood (the Patient Health Questionnaire–9), anxiety (the Generalized Anxiety Disorder Questionnaire–7), and relationship adjustment (the Revised Dyadic Adjustment Scale). As this was a low-risk study, only serious adverse events were recorded.

Fidelity was assessed on approximately 10% of sessions (77 of 777) by 2 VIPP-SD–trained assessors (interrater reliability; intraclass correlation, 0.69) using a 5-point scale of manual adherence. A score of 3 indicated the presence of most core intervention components.

### Changes to Protocol

The posttreatment assessment was changed from 4 to 5 months after randomization early in the study, as the full intervention was not always completed within this time frame.

### Sample Size and Statistical Analysis

Analysis was performed on an intention-to-treat basis. Results were interpreted based on point estimates and 95% CIs. The target sample size was 300 families, which would provide 80% power to detect a standardized effect size of 0.36 and 90% power to detect a standardized effect size of 0.42 at the 5% significance level, assuming 20% attrition (based on mean comparison at follow-up, with greater anticipated power because analyses adjust for baseline scores). A pooled effect size on child behavior was not available at study design. However, the sample size was considered reasonable because the effect size was 0.46 for previous RCTs of VIPP-SD on parental sensitivity and 0.69 from a National Institute for Health and Care Excellence systematic review of interventions targeting behavior problems,^29^ albeit in older children using researcher-rated outcomes.

Statistical analysis was performed from September 5, 2019, to January 17, 2020. The primary analysis was based on multiple linear regression to assess the primary outcome: group difference in mean PPACS score at 5 months after randomization. The PPACS score at 5 months was the dependent variable; trial group, PPACS score at baseline, time since randomization, recruitment center, age of child at recruitment, and number of parents or caregivers participating (1 or 2) were included as independent variables. Similar models, with adjustment for the appropriate baseline score, assessed difference in CBCL and SDQ scores between the trial groups at 5 months. Missing data for individual PPACS items at baseline and completely missing PPACS scores at 5 months were imputed. Missing items in the other outcomes (parental discipline, mood, anxiety, and couple functioning) were scaled up.

For imputation of the primary outcome, the level, pattern, and likely causes of missingness in the baseline variables and primary outcome were investigated. The imputation of individual missing items when the PPACS interview was mostly but not fully completed was based on completed items and subscales from baseline PPACS (for baseline scores) and PPACS at 5 months (for 5-month scores). Multiple imputation of the whole PPACS score when the entire scale was missing (it was never missing at baseline) was based on randomized group, child sex, child age at 5-month assessment, and baseline PPACS, CBCL, and SDQ scores.

As sensitivity analyses, analysis of the primary outcome was repeated without adjustment for time since randomization and using complete cases after multiple imputation of missing items only, assuming that losses to follow-up do better or worse than expected per multiple imputation and assuming the highest and lowest possible scores for missing items. Secondary analysis involved complier average causal effects analysis using 2-stage, least-squares regression analysis to determine the effect of actually receiving the intervention, predefined as receipt of 4 core VIPP-SD visits.

Planned subgroup analyses of the primary outcome involved the effects of child age at baseline (12-23 months vs 24-36 months) and the number of caregivers participating (1 vs 2). We also undertook post hoc subgroup analyses to assess the effect of severity of child behavior problems at study entry (by quartile of baseline SDQ score) and to compare White caregivers with caregivers of all other racial/ethnic groups combined.

Standardized effect sizes were calculated as Cohen *d* by dividing the mean difference (from the linear regression models) by the SD at follow-up in the UC group. Statistical analyses used Stata, versions 13 and 15 (StataCorp LLC). A data monitoring and ethics committee oversaw the study. Serious adverse events were compared between groups using the Fisher exact test.

## Results

A total of 2248 potential participants were screened between July 30, 2015, and July 26, 2017. Of these, 300 eligible participants consented to the trial and were randomly allocated to receive either VIPP-SD plus UC (n = 151) or UC alone (n = 149). The Figure shows participant flow. Table 1 shows that baseline characteristics of participants and their children (mean [SD] age, 23.0 [6.7] months) were generally well balanced between groups but that there were slightly more male children in the UC group than the VIPP-SD group (87 [58%] vs 76 [50%]) and there were small differences in reported race/ethnicity because the randomization was not stratified by these factors (see eTable 1 in Supplement 2 for baseline characteristics of second caregivers).

**Figure.**
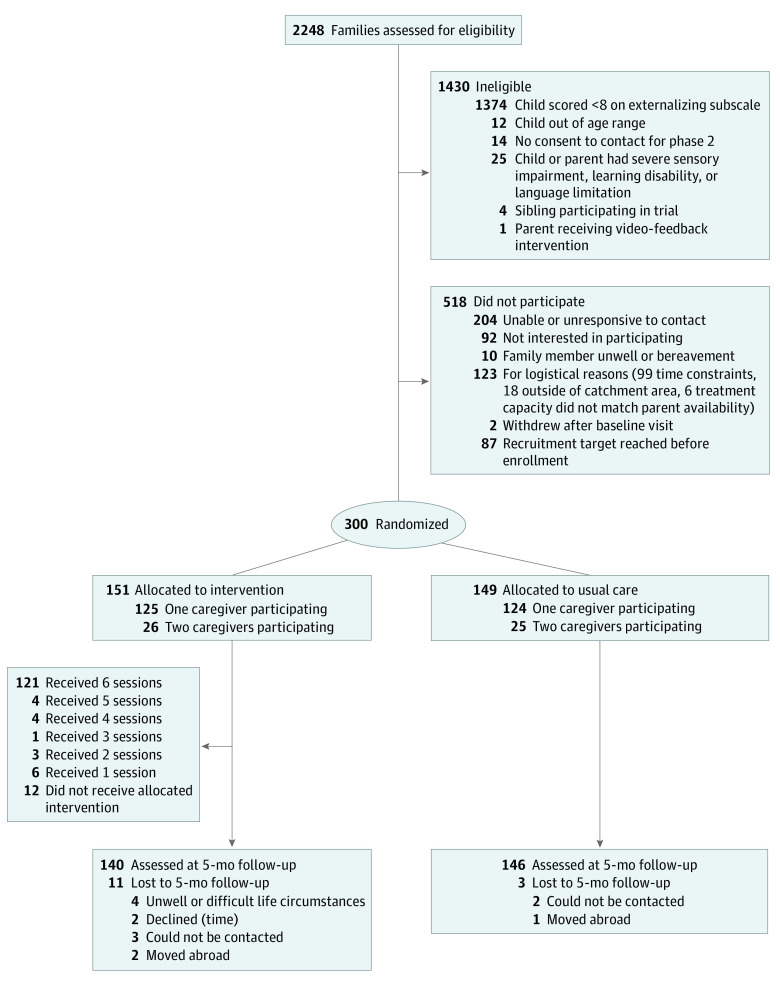
CONSORT Flow Diagram of Participants

**Table 1.** Baseline Characteristics of Participants by Treatment Allocation

Characteristic	Trial group, No. (%)	
	VIPP-SD (n = 151)	UC (n = 149)
**Children**		
Male	76 (50)	87 (58)
Age, mean (SD), mo	22.8 (6.8)	23.2 (6.5)
Race/ethnicity^a^		
White	100 (66)	94 (63)
Mixed	36 (24)	25 (17)
Asian	9 (6)	8 (5)
Black	3 (2)	15 (10)
Other	3 (2)	7 (5)
**Primary caregivers**		
Male	8 (5)	5 (3)
Age, mean (SD), y	33.7 (5.6)	34.7 (5.9)
Race/ethnicity^a^		
White	114 (75)	103 (69)
Mixed	11 (7)	11 (7)
Asian	15 (10)	16 (11)
Black	3 (2)	15 (10)
Other	8 (5)	4 (3)
Employment status		
Employed	66 (44)	64 (43)
Paid parental leave	6 (4)	10 (7)
Self-employed	20 (13)	12 (8)
Student	3 (2)	7 (5)
Looking after home and children	56 (37)	56 (38)
Highest qualification		
GCSE or lower	17 (11)	14 (9)
A level, NVQ, or BTEC	42 (28)	36 (24)
University graduate or postgraduate degree	92 (61)	99 (66)

Abbreviations: BTEC, Business and Technology Education Council; GCSE, General Certificate of Secondary Education; NVQ, National Vocational Qualification; UC, usual care; VIPP-SD, Video-feedback Intervention to promote Positive Parenting and Sensitive Discipline.

^a^ White includes White, White British, White Irish, and any other White background; mixed includes mixed White and Black Caribbean, mixed White and Black African, mixed White and Asian, and any other mixed background; Asian includes Asian or Asian British Indian, Asian or Asian British Pakistani, Asian or Asian British Bangladeshi, Asian or Asian British Chinese, and any other Asian background; Black indicates Black or Black British Caribbean, Black or Black British African, and any other Black background; and other includes any other ethnicity not represented in these 16 categories.

Treatment adherence was high: 121 of 151 (80%) participants received all 6 sessions, and 129 of 151 (85%) received 4 or more sessions (the cutoff for treatment adherence). Treatment fidelity was also high: 72 of the 77 randomly assessed sessions (94%) met the minimum fidelity threshold. The mean (SD) score for fidelity across sessions was 3.66 (0.60).

Child behavior outcome results are given in Table 2. There was a difference in mean PPACS score (primary outcome) between the trial groups (adjusted mean difference, 2.03 [95% CI, 0.06-4.01]; *P* = .04; *d* = 0.20 [95% CI, 0.01-0.40]), with fewer behavior problems in children in the VIPP-SD group (mean [SD] PPACS score, 28.8 [9.2]) compared with the UC group (mean [SD] PPACS score, 30.3 [9.9]). There was a difference in scores on the PPACS conduct problems subscale between the trial groups (mean difference, 1.61 [95% CI, 0.44-2.78]; *P* = .007; *d* = 0.30 [95% CI, 0.08-0.51]) but little difference in scores on the hyperactivity subscale (mean difference, 0.29 [95% CI, −1.06 to 1.65]; *P* = .67; *d* = 0.05 [95% CI, –0.17 to 0.27]). The mean group differences in primary caregiver–reported CBCL and SDQ scores were also consistent with the primary outcome findings (see subscale analysis in eTable 2 in Supplement 2). There were no appreciable group differences in the other secondary outcomes of parent or caregiver–reported disciplinary behavior, mood, anxiety, or couple functioning (Table 3).

**Table 2.** Child Behavior Outcomes

Outcome	VIPP-SD group^a^		UC group^a^		Adjusted mean difference (95% CI)^b^	Standardized effect size (95% CI)^c^	*P* value
	No.	Mean (SD)	No.	Mean (SD)			
**Primary outcome**							
Preschool Parental Account of Childhood Symptoms: total							
Baseline	151	33.5 (9.0)	149	32.4 (10.6)	NA	NA	NA
5-mo Follow-up	140	28.8 (9.2)	146	30.3 (9.9)	2.03 (0.06 to 4.01)	0.20 (0.01 to 0.40)	.04
Preschool Parental Account of Childhood Symptoms: conduct							
Baseline	151	16.0 (5.8)	149	15.5 (6.4)	NA	NA	NA
5-mo Follow-up	140	14.8 (5.1)	146	15.8 (5.4)	1.61 (0.44 to 2.78)	0.30 (0.08 to 0.51)	.007
Preschool Parental Account of Childhood Symptoms: ADHD							
Baseline	151	17.5 (5.8)	149	16.9 (6.6)	NA	NA	NA
5-mo Follow-up	140	14.0 (6.1)	146	14.5 (6.2)	0.29 (−1.06 to 1.65)	0.05 (−0.17 to 0.27)	.67
**Secondary outcomes**							
Child Behavior Checklist: total							
Baseline	151	40.7 (21.7)	149	42.7 (21.1)	NA	NA	NA
5-mo Follow-up	140	32.5 (20.6)	145	37.2 (21.0)	3.24 (−0.06 to 6.54)	0.15 (0.00 to 0.31)	.05
Strength and Difficulties Questionnaire: total							
Baseline	150	13.8 (4.8)	149	14.0 (4.7)	NA	NA	NA
5-mo Follow-up	140	11.3 (5.1)	145	12.2 (5.2)	0.93 (−0.03 to 1.9)	0.18 (−0.01 to 0.36)	.06

Abbreviations: ADHD, attention-deficit/hyperactivity disorder; NA, not applicable; UC, usual care; VIPP-SD, Video-feedback Intervention to promote Positive Parenting and Sensitive Discipline.

^a^ Lower scores indicate fewer behavior problems.

^b^ Difference in mean is the difference between treatment groups from linear regression analysis of the outcome measure on the baseline score of that same measurement, on treatment center, on randomized group, on length of follow-up, on age of child, and on number of caregivers participating (all treated as fixed effects). Positive differences represent greater adjusted decreases in symptoms in the VIPP-SD rather than in the UC group.

^c^ Standardized effect size is the standardized difference in mean (adjusted as above) and is presented as Cohen *d*, the difference in mean divided by the SD of controls at follow-up. Cohen *d* values of 0.4 to 0.6 represent typical values of Cohen *d*. These values do not directly relate to the clinical importance of the results because an assessment of a characteristic of each individual scale or subscale is required.

**Table 3.** Remaining Secondary Outcomes: Caregiver-Reported Parenting and Mental Health

Outcome	VIPP-SD group^a^		UC group^a^		Mean difference (95% CI)^b^	Standardized effect size (95% CI)^c^	*P* value
	No.	Mean (SD)	No.	Mean (SD)			
**Mothers or female caregivers**							
Parenting practice (Parenting Scale)							
Baseline	146	2.96 (0.52)	147	2.95 (0.58)	NA	NA	NA
5-mo Follow-up	135	2.90 (0.50)	143	2.90 (0.60)	0.06 (−0.22 to 1.23)	0.11 (−0.37 to 2.06)	.22
Parental or caregiver mood (PHQ-9)							
Baseline	145	4.34 (4.00)	147	4.28 (4.35)	NA	NA	NA
5-mo Follow-up	135	3.99 (4.49)	144	4.20 (4.71)	0.25 (−0.69 to 1.20)	0.05 (−0.15 to 0.25)	.60
Parental or caregiver anxiety (GAD-7)							
Baseline	145	4.89 (4.33)	147	4.73 (4.22)	NA	NA	NA
5-mo follow-up	134	4.29 (4.46)	144	3.92 (4.00)	0.05 (−0.85 to 0.95)	0.01 (−0.21 to 0.24)	.91
Parental or caregiver couple functioning (RDAS)							
Baseline	130	49.18 (8.36)	126	50.50 (9.22)	NA	NA	NA
5-mo Follow-up	118	49.19 (9.32)	120	49.92 (9.59)	0.20 (−1.44 to 1.83)	0.02 (−0.15 to 0.19)	.81
**Fathers or male caregivers**							
Parenting practice (Parenting Scale)							
Baseline	31	2.98 (0.54)	25	2.78 (0.48)	NA	NA	NA
5-mo Follow-up	29	2.90 (0.50)	24	2.89 (0.41)	0.10 (−0.13 to 0.33)	0.24 (−0.32 to 0.80)	.39
Parental or caregiver mood (PHQ-9)							
Baseline	31	3.03 (2.64)	25	2.56 (2.96)	NA	NA	NA
5-mo Follow-up	29	2.48 (2.34)	24	3.25 (3.57)	0.63 (−0.86 to 2.12)	0.18 (−0.24 to 0.60)	.40
Parental or caregiver anxiety (GAD-7)							
Baseline	31	2.35 (2.69)	25	2.84 (2.98)	NA	NA	NA
5-mo Follow-up	29	2.21 (2.32)	24	3.21 (3.66)	−0.04 (−1.54 to 1.45)	−0.01 (−0.42 to 0.40)	.95
Parental or caregiver couple functioning (RDAS)							
Baseline	31	50.56 (5.20)	25	50.23 (6.51)	NA	NA	NA
5-mo Follow-up	29	50.38 (6.42)	24	48.31 (8.80)	0.11 (−3.42 to 3.65)	0.01 (−0.39 to 0.41)	.95

Abbreviations: GAD-7, Generalized Anxiety Disorder–7; NA, not applicable; PHQ-9, Patient Health Questionnaire–9; RDAS, Revised Dyadic Adjustment Scale; UC, usual care; VIPP-SD, Video-feedback Intervention to promote Positive Parenting and Sensitive Discipline.

^a^ Parenting Scale: higher scores indicate more ineffective parenting strategies. PHQ–9: higher scores represent higher symptom severity. GAD–7: higher scores represent higher symptom severity. RDAS: higher scores indicate greater relationship satisfaction.

^b^ Difference in mean is the difference between treatment groups from linear regression analysis of the outcome measure on the baseline score of that same measurement, on treatment center, on randomized group, on length of follow-up, on age of child, and on number of caregivers participating (all treated as fixed effects). Positive differences represent greater adjusted decreases in symptoms in the VIPP-SD rather than in the UC group.

^c^ Standardized effect size is the standardized difference in mean (adjusted as above) and is presented as Cohen *d*, the difference in mean divided by the SD of controls at follow-up. Cohen *d* values of 0.4 to 0.6 represent typical values of Cohen *d*. These values do not directly relate to the clinical importance of the results because an assessment of a characteristic of each individual scale or subscale is required.

Sensitivity analyses (eFigure 1 in Supplement 2) suggest that the difference in PPACS scores between the trial groups is approximately 2 under most alternatives. Assuming that losses to follow-up were associated with better outcomes gave an estimated treatment effect of 2.42, and replacing missing items with the maximum possible score gave an estimated treatment effect of 2.56. Complier average causal effects analysis suggested that the treatment effect was stronger in those with acceptable levels of treatment adherence (≥4 core VIPP-SD sessions; mean difference between trial groups per PPACS, 2.59 [95% CI, 0.24-4.94; *d* = 0.26]; mean difference between trial groups per CBCL, 3.56 [95% CI, 0.04-7.09; *d* = 0.17]; mean difference between trial groups per SDQ, 1.03 [95% CI, −0.01 to 2.06; *d* = 0.20]) (eTable 3 in Supplement 2).

Subgroup analyses (eFigure 2 in Supplement 2) all had substantially overlapping 95% CIs; hence, there is considerable uncertainty regarding conclusions. Treatment effects appear to be greater in younger children, in families with 1 participating caregiver, and in those with the worst baseline behavior scores. There were no clear differences between racial/ethnic groups: the adjusted difference in mean PPACS score between treatment groups is 2.17 (95% CI,−0.13 to 4.47) in White primary caregivers and is 2.45 (95% CI,−1.90 to 6.79) in caregivers of other racial/ethnic groups.

In families who received VIPP-SD, 3 of 139 children (2%) had serious adverse events compared with 4 of 161 children from other families (3%; *P* = .60). All serious adverse events were unrelated inpatient hospital admissions (eg, accidents or respiratory infections). The mean (SD) follow-up was 161 (26) days from randomization in UC and 204 (62) days in the VIPP-SD group. Protocol deviations and violations pertained mainly to noncompletion of VIPP-SD visits and/or noncompletion or delays in posttreatment assessments (eTable 4 in Supplement 2).

## Discussion

We found clear evidence that a brief, home-based intervention, VIPP-SD, was more effective than UC in reducing behavior problems in this group of 1-year-old and 2-year-old children. Video-feedback Intervention to promote Positive Parenting and Sensitive Discipline uses video feedback from sessions involving a parent’s or caregiver’s interactions with their child, where the therapist shares feedback to promote sensitive responding and consistent discipline. Evidence of superiority was found for the primary outcome (the interview-based PPACS assessment), with results on the 2 measures of child problems (CBCL and SDQ questionnaires) being consistent with this finding. The secondary analysis of treatment compliance further supported the intervention’s efficacy such that families who received the core number of VIPP-SD sessions (≥4) demonstrated a larger improvement in behavior. The effect size for the VIPP-SD intervention (0.20) is somewhat difficult to interpret for an individual child or family; however, it represents a mean difference of 2 points on the PPACS measure. By way of example, for tantrums, this 2-point difference would equate to a change from severe (breaking things) to mild (shouting) or a change in frequency from daily to once or twice per week. For destructiveness, this 2-point difference would be a change from deliberately destroying items in the home or causing mild damage outside the home to no destructive behavior. These examples demonstrate that a 2-point difference represents a clear change in child behavior.

The beneficial effect of VIPP-SD appeared to be most prominent in the PPACS assessment of conduct problems rather than attention problems. This finding is in keeping with the sensitive discipline focus of the intervention, which targets conduct problems. Evidence since this trial was designed also suggests that conduct problems in particular are associated with poorer long-term outcomes for children and are more amenable to treatment than early attention-deficit/hyperactivity disorder or hyperkinesis problems.^30,31,32^ The findings also suggest that the effect of treatment may be greater in those with higher levels of symptoms and that the intervention is at least as effective for 1-year-old children as for 2-year-old children.

Previous smaller studies of VIPP-SD have shown similar findings, with a meta-analysis demonstrating a similar effect size for child behavior.^16^ However, to our knowledge, this is the first pragmatic trial of VIPP-SD conducted in a routine NHS health care setting, and the findings are robust in this setting. The intervention is also acceptable for a psychological treatment as indicated by the high levels of adherence. A substantial body of evidence for the efficacy of several interventions already exists for childhood behavior problems; however, there is much less evidence for very young children in routine practice and no evidence of this kind in the UK. This is the first effectiveness study, to our knowledge, to demonstrate a beneficial treatment effect in 1-year-old children and in routine practice. This finding is important, as there are many examples of promising interventions for child mental health problems that do not show a clear benefit when used in routine care despite initial evidence of efficacy in other more tightly controlled trial conditions.^33,34,35^ Consequently, these findings, including high levels of fidelity, indicate that the intervention can be reliably and successfully delivered in routine NHS practice by health visitors and community nursery nurses. The study findings also add to encouraging evidence for other interventions focused on improving caregiver sensitivity in community settings, such as Attachment and Biobehavioral CatchUp.^36,37^

We did not observe treatment effects on parental mood, anxiety, relationship adjustment, or self-reported discipline. Evidence suggests that parenting programs may not be sufficiently powerful in and of themselves to shift global indicators of distress.^38^ Alternative measures of parental factors, such as parental self-efficacy, may be useful for future trials, as this may be one pathway by which interventions like VIPP-SD exert their effect.

### Strengths and Limitations

This study has some strengths, including its multicenter pragmatic design, which recruited families in routine health care settings in urban, suburban, and rural settings in the UK. The VIPP-SD intervention was acceptable to participants, with a very high level of retention (95%). This level of retention may result in part because of the intervention’s home delivery, which is likely to be more acceptable for families. The study also benefited from an interview measure of child behavior, which allowed the researcher to gather detailed information about symptoms based on both their severity and frequency that was not weighted by the parent but by the researcher according to strict criteria.

This study also has some limitations. It did not include a formal assessment of impairment, which is challenging to do in younger children. Secondary outcomes relied on parent-completed questionnaires, and no other reporters of child behavior were used in this assessment. The SDQ and CBCL have limited previous use in children as young as 1 year. Uptake of the invitation to participate in the study was in line with similar trials but could possibly be improved in clinical practice with more direct contact with families and use of motivational techniques. Like many RCTs, the study participants had somewhat higher levels of education than the national average, although in most other respects they were similar to families in recruitment areas. In addition, we found that not all therapists were able to deliver the intervention, largely owing to service changes, which meant that planned time to deliver the treatment became restricted. Further research is needed to examine the long-term implications of the intervention.

## Conclusions

The findings of this RCT of VIPP-SD, a brief, home-based parenting intervention, demonstrated that the intervention was effective at reducing behavior problems, particularly conduct problems, in very young children. The study indicates that there is substantial potential for VIPP-SD to be delivered successfully by community health staff in routine practice. Despite a strong global policy focus on early intervention, a lack of effective interventions is one of the key problems holding back the field from improving children’s health, education, and social outcomes.^39,40,41^ Thus, the results of this study represent a new opportunity for effective early childhood intervention to prevent mental health problems.

## Notes

Supplement 1. Trial Protocol

Supplement 2. eTable 1. Baseline Characteristics of Participating Secondary Caregivers by Treatment Allocation eTable 2. ITT Analysis of Change in Children’s Behavior Problems on the Secondary Outcome Subscales (Primary Caregiver Reported CBCL and SDQ) at 5-Month Follow-up eTable 3. Complier Average Causal Effects Analysis on Primary and Secondary Outcomes of Child Behavior eTable 4. Occurrence of Protocol Deviations and Violations eFigure 1. Forest Plot for Sensitivity Analysis at 5-Month Follow-up eFigure 2. Subgroup Analysis of the Primary Outcome (PPACS Total) by Age of Child, Number of Participating Caregivers, and Baseline (SDQ-Externalizing) Behavior

Supplement 3. Data Sharing Statement
