# Supplementary material for: A Brief Home-Based Parenting Intervention to Reduce Behavior Problems in Young Children: A Pragmatic Randomized Clinical Trial
Source: JAMA Pediatr. 2021 Mar 15;175(6):1–10. doi: 10.1001/jamapediatrics.2020.6834 (PMC7961467; doi:10.1001/jamapediatrics.2020.6834)
Supplement: Supplement 1. — Trial Protocol [file jamapediatr-e206834-s001.pdf]

Supplementary File: A brief home-based parenting intervention (VIPP-SD) to prevent enduring behaviour problems in young children: A pragmatic randomised clinical trial.

eDocument 1. Protocol

eDocument 2. Pre-specified statistical analysis plan

eDocument 1. Protocol

CLINICAL TRIAL PROTOCOL

Study Title: Preventing enduring behavioural problems in young children through early psychological intervention: Healthy Start, Happy Start

Protocol Number: 14HH2370

Sponsor: Imperial College London

Draft Protocol Version: 6.0

Date: 3rd July 2017

Property of: Imperial Clinical Trials Unit (ICTU)

May not be used, divulged or published without the consent of ICTU

CONTACT LIST

Chief Investigator

Professor Paul Ramchandani<br>Reader in Child and Adolescent Psychiatry<br>The Centre for Psychiatry<br>Hammersmith Hospital Campus<br>Imperial College London<br>7th Floor Commonwealth Building<br>Du Cane Road<br>London<br>W12 0NN<br><br>Tel: 020 8383 4161<br>Email: p.ramchandani@imperial.ac.uk

Sponsor

AHSC Joint Research Compliance Office<br>Imperial College London<br>Room 15<br>Medical School Building<br>St Marys Campus<br>Norfolk Place<br>London W2 1PG<br><br>Contact person: Becky Ward<br>Tel: +44 (0) 0207 594 9459<br>Email: becky.ward@imperial.ac.uk

Funder

National Institute for Health Research Health Technology Assessment Programme (NIHR HTA)<br>National Institute for Health Research<br>Evaluation, Trials and Studies Coordinating Centre<br>University of Southampton<br>Alpha House, Enterprise Road<br>Southampton SO16 7NS<br><br>Tel +44 (0) 23 8059 5586<br>Fax +44 (0) 23 8059 5639<br>Email: info@netscc.ac.uk

ICTU Operations Manager

Daphne Babalis<br>Imperial Clinical Trials Unit<br>Imperial College London<br>Stadium House<br>68 Wood Lane<br>London, W12 7RH<br><br>Tel: 0207 594 3403<br>e-mail: d.babalis09@imperial.ac.uk

Trials Coordination

Imperial Clinical Trials Unit (ICTU)
Stadium House
68 Wood Lane
London, W12 7RH

Trial Statistician

Hilary Watt
Consultant Statistician
Primary Care & Public Health
School of Public Health
Charing Cross Campus
Imperial College London

Tel: 020 7594 7451
Fax: 020 7594 0854
Email: h.watt@imperial.ac.uk

Senior Statistician

Jane Warwick
Associate Professor in Clinical Trials Statistics
Warwick Clinical Trials Unit, Division of Health Sciences, Warwick Medical School
University of Warwick
Coventry CV4 7AL

Tel: + 44 (0)2476 522498
Email: j.warwick@warwick.ac.uk

Other Participating Investigators

Stephen Scott
Institute of Psychiatry
Denmark Hill
SE5 8AF

Tel: +44 (0)20 7848 0746
Email: stephen.scott@kcl.ac.uk

Alan Stein
University of Oxford Department of Psychiatry,
Warneford Hospital
Oxford
OX3 7JX

Tel: +44 (0)1865 223911
Fax: +44 (0)1865 793101
Email: alan.stein@psych.ox.ac.uk

Sarah Byford
David Goldberg Centre
Denmark Hill
London

SE5 8AF

Tel: +44 (0)20 7848 0043
Email: s.byford@kcl.ac.uk

Marian Bakermans-Kranenburg
Pieter de la Court gebouw
Wassenaarseweg 52
2333 AK Leiden

Tel: +31 (0)71 527 3798
Email: bakermans@fsw.leidenuniv.nl

Marinus van IJzendoorn
Pieter de la Court gebouw
Wassenaarseweg 52
2333 AK Leiden

Tel: +31 (0)71 527 3435
Email: vanijzen@fsw.leidenuniv.nl

ABBREVIATIONS

AE	Adverse Event
CA-SUS	Child and Adolescent Service Use Schedule
CI	Chief Investigator
CBCL	Child Behaviour Checklist
DMEC	Data Monitoring and Ethics Committee
eCRF	Electronic Case Report Form
GAD7	Generalized Anxiety Disorder 7
ICF	Informed Consent Form
ICTU	Imperial Clinical Trials Unit
PIS	Patient Information Sheet
PMG	Project Management Group
Pre-PACS	Preschool Parent Account of Child's Symptoms
PHQ-9	Patient Health Questionnaire 9
QA	Quality Assurance
RDAS	Revised Dyadic Adjustment Scale
REC	Research Ethics Committee
SAE	Serious Adverse Event

SAP	Statistical Analysis Plan
SOP	Standard Operating Procedure
SSAR	Suspected Serious Adverse Reaction
SUSAR	Suspected Unexpected Serious Adverse Reaction
TSC	Trial Steering Committee
VIPP-SD	Video-Feedback Intervention to Promote Positive Parenting and Sensitive Discipline

Table of Contents

1. TRIAL SUMMARY ... 10
2. INTRODUCTION AND RATIONALE ... 11
3. AIMS, hypothesis and OBJECTIVES ... 12
   3.1. Aim ... 12
   3.2. Primary hypothesis ... 12
   3.3. Secondary hypotheses ... 12
4. Objectives ... 12
5. Outcome measures ... 12
   5.1. Primary endpoint ... 12
   5.2. Secondary endpoints ... 12
   5.3. Exploratory endpoints ... 13
6. STUDY Design ... 13
7. Trial Intervention ... 13
   7.1. ViPP-SD ... 13
      7.1.1. Treatment Fidelity ... 13
   7.2. Usual care ... 13
   7.3. Qualitative interviews with families ... 14
   7.4. Qualitative interviews with therapists ... 14
8. Flow Chart ... 15
9. EARLY DISCONTINUATION OF THE STUDY OR WITHDRAWAL OF INDIVIDUAL participants ... 16
   9.1. Early Discontinuation of the Study ... 16
   9.2. Withdrawal of Individual participants ... 16
10. Study population ... 16
   10.1. Eligibility criteria ... 16
      10.1.1. Inclusion criteria ... 16
      10.1.2. Exclusion criteria ... 16
11. study Procedures ... 16
   11.1. Phase 1 ... 16
   11.2. Phase 2 ... 17
   11.3. Blinding ... 18
   11.4. Randomisation ... 18
   11.5. Follow-up visits ... 18
   11.6. Assessment Visit Schedule ... 19
   11.7. ViPP-SD Visit Schedule ... 19
   11.8. Measures ... 20
      11.8.1. Pre-PACS ... 20
      11.8.2. CBCL ... 20
      11.8.3. SDQ ... 20

11.8.4. RDAS ... 20
11.8.5. Patient Health Questionnaire 9 ... 21
11.8.6. Parenting Scale ... 21
11.8.7. CA-SUS ... 21
11.8.8. GAD-7 ... 21
11.8.9. AUDIT-C ... 21
11.8.10. Parental sensitivity ... 21
11.8.11. Parental involvement ... 21
11.8.12. Temper tantrum screen ... 21
11.8.13. Emotion regulation ... 22
11.8.14. Executive function ... 22
11.8.15. Prosocial behaviour ... 22
11.8.16. Genetic Variance ... 22
11.8.17. Feedback questionnaire ... 22
12. Safety Reporting ... 22
12.1. Adverse Event (AE) ... 22
12.2. Severity of Adverse Events ... 22
12.3. Causality of Adverse Events ... 22
12.4. Serious Adverse Events (SAE) ... 23
12.4.1. Definition of SAE ... 23
12.4.2. Reporting of SAEs ... 23
12.5 Definition of a Serious Adverse Reaction (SAR) ... 24
12.6.1 Reporting of Related and Unexpected Serious Adverse Events ... 24
12.7 Annual reporting of SAEs ... 24
13. STATISTICAL ANALYSES ... 24
13.1. Sample Size and power considerations ... 24
13.2. Data Analysis ... 24
13.3. Economic analysis ... 25
13.3.1. Short-term cost-effectiveness ... 25
13.3.2. Long-term cost-effectiveness ... 25
14. Patient and public involvement (PPI) ... 26
15. REGULATORY, ETHICAL AND LEGAL ISSUES ... 26
15.1. Research Ethics Committee (REC) Approval ... 26
15.2. Approval of Amendments ... 26
15.3. Addition onto trial register ... 27
15.4. Annual Progress Reports ... 27
15.5. End of Trial Notification ... 27
15.6. NHS Health Research Authority Study Approval ... 27
15.7. Informed Consent ... 27

15.8. Contact with General Practitioner and Health visitor....28
15.9. Patient Confidentiality....28
16. End of Trial....28
17. ADMINISTRATIVE MATTERS....28
17.1. Source Data....28
17.2. Language....28
17.3. Data collection and management....28
17.4. Study Documentation and Data Storage....29
17.5. Study Management Structure....29
17.6. Trial Steering Committee (TSC)....29
17.7. Data Monitoring and Ethics Committee (DMEC)....29
17.8. Project Management Group (PMG)....29
17.9. Patient Advisory Group (PAG)....29
17.10. Target organisations....29
17.11. Monitoring....30
17.12. Quality Control and Quality Assurance....30
17.13. Publication policy....30
REFERENCES....31
SIGNATURE PAGE 1 (Chief Investigator).... **Error! Bookmark not defined.**
SIGNATURE PAGE 2 (SPONSOR).... **Error! Bookmark not defined.**
SIGNATURE PAGE 3 (STATISTICIAN).... **Error! Bookmark not defined.**
SIGNATURE PAGE 4 (PARTICIPATING INSTITUTIONAL APPROVAL).... **Error! Bookmark not defined.**
SIGNATURE PAGE 5 (INVESTIGATOR).... **Error! Bookmark not defined.**

1. TRIAL SUMMARY

**TITLE**	Preventing enduring behavioural problems in young children through early psychological intervention: Healthy Start, Happy Start
**OBJECTIVES**	**Primary objective**<br>To undertake a randomised controlled trial to evaluate whether compared to treatment as usual, a brief parenting intervention (Video Feedback to Promote Positive Parenting and Sensitive Discipline) leads to lower levels of behavioural problems in young children who are at high risk of developing these difficulties.<br><br>**Secondary objective**<br>To undertake an economic evaluation to assess the cost-effectiveness of the intervention compared to treatment as usual.
**DESIGN**	Randomised, parallel, two-arm controlled trial
**SAMPLE SIZE**	300 (150 in each arm)
**STUDY POPULATION**	Parents/caregivers of children aged 12-36 months and their infants
**ELIGIBILITY CRITERIA**	**Inclusion criteria**<br>1. Parents aged ≥ 18 years<br>2. Child aged between approximately 12-36 months<br>3. Child scores in the top 20% for behavioural problems on the Strengths and Difficulties Questionnaire (SDQ), based on population norms<br>4. Written informed parental/carer consent<br><br>**Exclusion criteria**<br>1. Child or parent has severe sensory impairment, learning disability, or language limitation, which is sufficient to preclude participation in the trial.<br>2. Siblings participating in trial<br>3. Families participating in active family court proceedings<br>4. Parent/carer is participating in another closely related research trial and/or is currently receiving an individual video-feedback based intervention.
**TREATMENT**	Video-Feedback Intervention to Promote Positive Parenting and Sensitive Discipline (VIPP-SD)
**PRIMARY ENDPOINT**	Assessment of severity of behavioural problems using the Pre-PACS interview at five months post-randomisation
**SECONDARY ENDPOINTS**	1. Child Behaviour assessed by the CBCL questionnaire<br>2. Child Behaviour assessed by the Strengths and Difficulties Questionnaire (SDQ)<br>3. Parental sensitivity in interactions with their child.<br>4. Parental mood assessed by the Parent Health Questionnaire 9<br>5. Parental anxiety assessed by the GAD-7<br>6. Couple functioning assessed by the Revised Dyadic Adjustment Scale<br>7. Parenting practice assessed by the Parenting Scale<br>8. Resource use using a modified version of the Child and Adolescent Service Use Schedule (CA-SUS)
**EXPLORATORY ENDPOINTS**	1. Parental involvement assessed using an adapted measure from Bronte-Tinkew et al. (2008)<br>2. Temper tantrum intensity assessed by the Egger Tantrum Screen (2016)<br>3. Emotion regulation assessed using the Attractive Toy in a Transparent Box task (Lab-TAB)<br>4. Executive function assessed using the Minnesota Executive Function Scale (MEFS)<br>5. Prosocial behaviour assessed using a dolls play task<br>6. Genetic variance assessed via genotyping and methylation of buccal samples

2. INTRODUCTION AND RATIONALE

Behavioural problems affect 5-10% of children, and children with established behavioural problems have significantly worse outcomes through childhood and into adult life. They have an increased risk of psychiatric disorders, antisocial behaviour and criminality, drug and alcohol misuse, educational failure and physical ill health. As well as these high levels of difficulties and unhappiness for young people and their families, there are also large costs incurred by society through the health, social care and criminal justice systems.

A key risk factor for the development of behavioural problems is the quality of the parental care that children receive: low levels of sensitive parenting and greater use of harsh discipline have been causally linked to the development of behavioural problems. Interventions which work with parents and carers to improve their parenting have been found to reduce child behavioural problems, and intervening early in children's lives has the potential to be particularly effective in improving outcomes, as well as having beneficial effects for parental health and wellbeing.

Most research to date has focussed on older children, when behavioural problems are more established, and thus more difficult to treat. Interventions have also focussed predominantly on mothers, with very few interventions involving fathers or a second caregiver, despite accumulating evidence that interventions involving two parents or caregivers can be more effective than those engaging just one. The proposed intervention (VIPP-SD) has a developing evidence base as an early preventive intervention (16-20) and has the potential to be delivered widely across the NHS as part of an early intervention programme. Young children and their carers have regular contact with the NHS, yet evidence is needed to ensure that resources are directed in the most effective manner. The trial has been designed to provide this evidence, as the first large randomised controlled trial to test whether an early video feedback intervention (ViPP-SD) is an effective and cost-effective approach to reducing behavioural problems in at-risk young children. It addresses an area of key concern to the NHS and represents an opportunity to reduce the burden of behavioural problems on individuals, families and society. If shown to be effective, the intervention could be delivered widely across the NHS to parents and carers of young children at risk of behavioural problems as part of community based services.

There are a number of systematic reviews (25-28) and policy-relevant reviews (29-31, 40) of this field. These highlight that intervening early in children's lives can be particularly effective in improving child outcomes, with evidence from three areas of research: i) trials that have tested interventions with parents of young children or with expectant parents (27, 32-34); ii) epidemiological work pointing to the potential importance of the earliest years of development in setting the trajectory for later outcomes (35-36), and iii) economic studies demonstrating the increased opportunities and cost returns achieved by effective early intervention (29, 37).

Some key early interventions for behavioural problems have been identified (27-28), including those which show promise for intervention on a wide scale. The Family Nurse Partnership (32) is being used across the UK and shows promise, but it is focussed on a limited target group and, as the Harvard Policy Review (30) points out: "No single program approach or mode of service delivery has been shown to be a magic bullet".

Alternative and complementary approaches are still needed, and Video Feedback (ViPP-SD) is a compelling alternative because it has already been evaluated and shown to improve mother-infant interaction (a key pathway for behavioural problems) (16-19), with initial evidence of improvement in child behaviour (20). Research also suggests that including two parents/caregivers in interventions, particularly fathers, may lead to increased efficacy (25, 38-39).

The ViPP intervention has been developed and evaluated in a systematic way, including six randomised controlled trials in different settings and with different groups of families. It has an evidence base for early preventive intervention with effects shown on parental sensitivity in parent-child interactions, positive parental discipline practices and child behaviour (16-20).

The intervention is derived from an understanding of attachment theory (41), whereby the promotion of sensitive parenting improves the relationship that children have with their primary caregiver. It begins with a core series of four sessions that aim to enhance the parent's capacity to identify the child's exploratory behaviour and attachment cues and to respond to them appropriately (1). Each session also includes an explicit focus on parental discipline strategies, based on the video recordings of the interactions with their own child. This incorporates aspects of social learning theory (42), with a focus on increasing positive and reducing aversive interactions. Overall, the intervention represents a powerful combination of the insights from the attachment and social learning perspectives (43).

The case for early preventive intervention is becoming increasingly established. The 2012 Chief Medical Officer's report, 'Our Children Deserve Better: Prevention Pays' (40), clearly highlights the social and economic benefits of early, preventive interventions in child health. The report focusses particularly on the need for interventions to improve the early parent-child relationship, as a way of reducing the risk of psychiatric disorder in children. However, it is essential that proposed early interventions are shown to be effective and cost-effective. This is the first RCT to test VIPP-SD in a UK setting. VIPP-SD has the potential to be an effective and cost-effective early intervention for behavioural problems.

3. AIMS, hypothesis and OBJECTIVES

3.1. Aim

To evaluate the effectiveness and cost-effectiveness of a brief early parenting intervention, designed to prevent enduring behavioural problems in young children aged 12-36 months old.

3.2. Primary hypothesis

Among children with high levels of behavioural problems aged twelve to thirty-six months, adding a brief video-feedback parenting intervention (ViPP-SD) to treatment as usual will reduce enduring behavioural problems measured at five months post-randomisation, using the Pre-PACS interview.

3.3. Secondary hypotheses

1) Among children with high levels of behavioural problems aged twelve to thirty-six months, adding a brief video-feedback parenting intervention (ViPP-SD) to treatment as usual will reduce enduring behavioural problems measured at two years post-randomisation, using the Pre-PACS interview.

2) Among children with high levels of behavioural problems aged twelve to thirty-six months, adding a brief video-feedback parenting intervention (ViPP-SD) to treatment as usual will reduce enduring behavioural problems measured at five months and two years post-randomisation, using the Child Behaviour CheckList (CBCL) and Strengths and Difficulties Questionnaire (SDQ), completed by parents/carers and the SDQ completed by a nursery carer/teacher.

3) Among children with high levels of behavioural problems aged twelve to thirty-six months, adding a brief video-feedback parenting intervention (ViPP-SD) to treatment as usual will result in higher levels of parental sensitivity in parent-child interactions, measured at 5 months.

4) Among children with high levels of behavioural problems aged twelve to thirty-six months, adding a brief video-feedback parenting intervention (ViPP-SD) to treatment as usual will provide a cost effective use of resources.

4. Objectives

1. To undertake a randomised controlled trial to evaluate whether, compared to treatment as usual in the NHS, a brief parenting intervention (Video Feedback to Promote Positive Parenting and Sensitive Discipline) leads to lower levels of behavioural problems in young children who are at high risk of developing these problems.
2. To undertake an economic evaluation to assess the cost-effectiveness of the intervention compared to treatment as usual.

5. Outcome measures

5.1. Primary endpoint

Assessment of severity of behavioural problems using the Pre-PACS interview at five months post-randomisation

5.2. Secondary endpoints

1. Child Behaviour assessed by the CBCL questionnaire
2. Strengths and Difficulties Questionnaire (SDQ)
3. Parental sensitivity in interactions with their child
4. Parental mood assessed by the Patient Health Questionnaire 9 (PHQ9)
5. Parental anxiety assessed by the Generalized Anxiety Disorder 7 (GAD7)
6. Parental couple functioning assessed by the Revised Dyadic Adjustment Scale (RDAS)Parenting practice assessed by the Parenting Scale

7. Resource use using a modified version of the Child and Adolescent Service Use Schedule (CA-SUS)

5.3. Exploratory endpoints

1. Parental involvement assessed using an adapted measure from Bronte-Tinkew et al. (2008)
2. Temper tantrum intensity assessed by the Egger Tantrum Screen (2016)
3. Emotion regulation assessed using the Attractive Toy in a Transparent Box task (Lab-TAB)
4. Executive function assessed using the Minnesota Executive Function Scale (MEFS)
5. Prosocial behaviour assessed using a dolls play task
6. Genetic variance assessed via genotyping and methylation of buccal samples

6. STUDY Design

The study is a two-arm, parallel group, researcher-blind, randomised controlled trial (RCT), to test the clinical and cost effectiveness of a video-feedback intervention (ViPP-SD) for parents of young children (12-36 months) at risk of behavioural difficulties. The trial will involve 300 families, who will be randomly allocated into one of two groups:

(1) The intervention group, who will receive the video-feedback intervention (described below) (n=150) plus treatment as usual

(2) Treatment as usual (control group) (n=150)

7. Trial Intervention

7.1. ViPP-SD

ViPP-SD is a home-based intervention, delivered over six sessions at approximately fortnightly intervals, which shows high levels of parental acceptability. Each session involves filming parent-child interactions and giving parents feedback based on these video clips. Adaptations have been made to account for treatment delivery to two parents/caregivers.

The intervention will be delivered by trained, supervised health professionals, predominantly health visitors. They will deliver the intervention in research participants' homes (or another location according to participant preference). The key role of the therapists will be to develop a trusting relationship with the participants in the treatment arm, and to deliver the treatment in 6 sessions in accordance with the manual. They will be supervised, and the treatment will be monitored closely for fidelity to the manual by the clinical supervisor (and a proportion will be taped and assessed by an independent researcher trained in the intervention).

- Four core sessions: these aim to enhance the parent's capacity to identify the child's exploratory behaviour and attachment cues and to respond to them appropriately
- Two booster sessions: these are spaced one month apart, and the key messages are repeated using continuing video interaction material at each session

Therapists responsible for delivering the intervention will be trained by the developers of ViPP-SD and will undertake supervised clinical practice before becoming a therapist on the trial.

7.1.1. Treatment Fidelity

Each therapist will be trained by an accredited VIPP trainer, and will undertake supervised clinical practice before becoming a therapist on the trial.

In order to determine treatment fidelity, the therapist will be asked to document, for each session, whether they delivered key components of the treatment as well as reporting on global adherence to the manual. All sessions will additionally be audio recorded to enable assessment of fidelity (on a random proportion) by independent raters. Preparatory work for the sessions, notes from sessions and the audio recordings will be used during monthly supervision with the lead clinical supervisor in the study, with oversight and regular review from the lead investigators.

7.2. Usual care

Participants in both groups will continue to receive their usual care. Usual care may include a range of services such as the following:

- health visitor services
- GP advice
- early intervention mental health services linked to children's centres
- parenting advice and support sessions

Data on concurrent use of health services will be collected including number of sessions offered, where they were provided, and which healthcare (or other non-healthcare) professionals provided the care.

7.3. Qualitative interviews with families

A proportion of families who receive treatment will also be invited to participate in a semi-structured qualitative interview to share information about their experiences of the VIPP intervention. A separate protocol provides further detail on this sub-study.

7.4. Qualitative interviews with therapists

Therapists delivering the treatment will be invited to participate in a semi-structured qualitative interview to share information about their experiences delivering the VIPP intervention. A separate protocol provides further detail on this sub-study.

8. Flow Chart

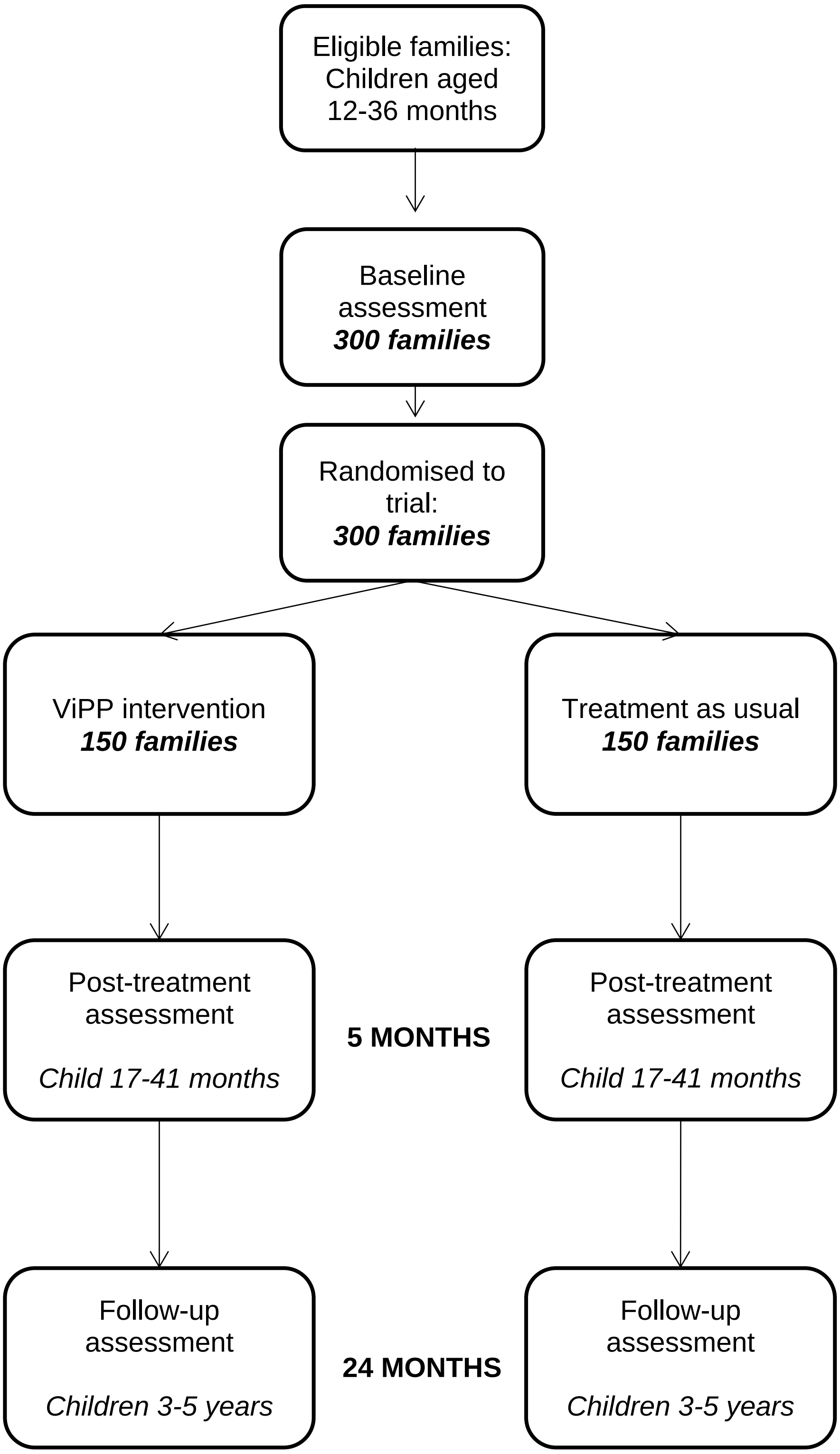
Eligible families: Children aged 12-36 months
Baseline assessment
300 families
Randomised to trial:
300 families
ViPP intervention
150 families
Treatment as usual
150 families
Post-treatment assessment
Child 17-41 months
5 MONTHS
Post-treatment assessment
Child 17-41 months
Follow-up assessment
Children 3-5 years
24 MONTHS
Follow-up assessment
Children 3-5 years

9. EARLY DISCONTINUATION OF THE STUDY OR WITHDRAWAL OF INDIVIDUAL participants

9.1. Early Discontinuation of the Study

The Data Monitoring and Ethics Committee (DMEC) for the trial will prepare a charter outlining their responsibilities and planned interim analyses. The charter will also define whether any stopping rules should be implemented for the trial.

If a decision to discontinue the trial prematurely is reached, a notification will be sent to the Research Ethics Committee within 15 days of the end date. The Project Management Group will assess how participants should be informed and whether follow-up visits to the families that have been recruited to the study should continue.

9.2. Withdrawal of Individual participants

Participants may discontinue the intervention or withdraw from the study for the following reasons:

- At the request of the child's family
- If the investigator considers that a participant's health will be compromised due to adverse events or concomitant illness that develop after entering the study

If a participant withdraws from the study intervention or from further follow-up visits, this should be documented in the participant records and electronic Case Report Form (eCRF) including the reason for withdrawal, whether study data collected up to that point can be used and whether further follow-up can be conducted.

10. Study population

Children aged 12-36 months with behavioural problems and their parents/carers.

10.1. Eligibility criteria

10.1.1. Inclusion criteria

1. Parents aged ≥ 18 years
2. Child aged between 12-36 months
3. Child scores in the top 20% for behavioural problems on the Strengths and Difficulties Questionnaire (SDQ), based on population norms.
4. Written informed parental consent

10.1.2. Exclusion criteria

1. Child or parent has severe sensory impairment, learning disability, or language limitation, which is sufficient to preclude participation in the trial.
2. Siblings participating in the trial
3. Families participating in active family court proceedings
4. Parent/carer is participating in another closely related research trial and/or is currently receiving an individual video-feedback based intervention.

11. study Procedures

11.1. Phase 1

Potential participants will be recruited from NHS health services via health visiting services, child and adolescent mental health services, GP services**,** and through links with children's centres and similar community services for families (such as family support services, libraries, and one o'clock clubs) working within the seven NHS study sites (Camden, Hillingdon, Islington, Oxfordshire, Barking and Dagenham, Peterborough, Hertfordshire). Other sites may be added as needed, subject to approval. Participants will be parent/s accessing these services already, therefore will already be known to local services.

Health visitors will recruit families to take part in the study at the routine 12 and 24-month health reviews. Clinicians and practitioners in other settings will recruit families when they are referred for support. These identified clinicians will be able to support recruitment by either signposting parents to the study, passing on screening packs**,** or by completing the screening stage of recruitment themselves. Members of the research team and clinical research network (CRN) support staff will also support recruitment of families on site in health visiting services and other venues.

Potential participants will be approached to take part in the screening process, via a questionnaire measure of child behaviour difficulties (The Strengths and Difficulties Questionnaire, SDQ) as well as providing some brief contact details which will enable the research team to make contact following screening and basic demographic variables (relationship to child, age, ethnicity, and educational attainment). The SDQ is a short questionnaire that is widely used in clinical practice assessing parental perception of child difficulties. This questionnaire is well validated and is widely used in clinical practice and research. Those scoring in the top 20% on population norms will then be invited to participate in the full study.

This screening questionnaire will be presented to participants as a pack, together with an invitation letter, an information sheet, consent form, and a freepost envelope regarding taking part in Phase 1 of the study (the screening stage of the recruitment). The written information will explain that they have the opportunity to discuss any questions with the health professional/CRN support staff member/or member of the research team, or call a member of the research team to discuss the study over the phone or email, using contact details contained on the information sheet. It will also be made clear that they will be able to withdraw their consent at any time and that they are providing consent to complete the screening questionnaire only, and not the full study (Phase 2).

The screening questionnaire pack will either be sent to participants as an enclosure in a standard letter being sent regarding the service (e.g. an invitation to attend a 12 or 24 month health review)**,** a separate mailshot if preferred by the service**,** or handed to potential participants when they attend the service. Those participants that received the screening questionnaire in the post will be invited (via written information in the pack) to return it to their healthcare professional at their subsequent appointment. They will also have the option of returning it directly to the research team in the post using an enclosed freepost envelope or filling it in online via a link to a secure website contained in the information sheet.

Those participants that are given the screening questionnaire pack in person when they attend the service, will have the opportunity to complete the SDQ and consent form whilst at the service, or they will be asked to complete the questionnaire and consent form to return by post or in person when next using the service or electronically using the link provided in the information sheet. Members of the research team or CRN support staff will also visit community centres such as children's centres and one o'clock clubs (e.g. during play, activity, information, and training sessions), with permission from management, to disseminate screening packs to families and where appropriate support families in completing the screening questionnaire, in line with the procedures outlined above. Screening packs will also be made available to families by staff in these settings and in GP practices.

Recruitment will also be supported via poster advertisements and flyers in these health and family service settings, as well as other community venues which cater for families (e.g., GP practices, libraries, nurseries, activity groups) and mailshots from these services. All poster/flyer adverts will contain an email address and phone number for parents to call if they are interested in taking part in the study. Recruitment will also be conducted via social media (facebook and twitter) and relevant online websites designed to support parents of young children, such as 'Netmums' and 'Mumsnet'. Information about the study will also be displayed on specific websites linked to the research team (e.g. http://www.ppod.org.uk). All information posted online will replicate that contained in the study's posters or leaflets. Information will also be provided through adverts in print media and on community radio stations, which will signpost families to the study team.

Those parents that see recruitment information or have been signposted to the study (e.g. via posters and leaflets displayed in health settings, or on internet advertisements) and therefore make contact directly with the research team will be provided with the same screening information pack to complete and return, if they so wish. It is estimated that in excess of 5,000 families will need to be screened in order to enrol the target sample of 300 participants in the trial.

11.2. Phase 2

Following screening, participants who score in the top 20% of population norms will be contacted by a member of the research team to see if they would like to participate in the full study.. For those that are selected, they will be contacted by phone and a date will be arranged for a member of the research team to visit them at home to complete the first assessment visit. At this stage it will be ascertained whether there are two parents in the family and whether both would like to take part.

This first assessment visit will take approximately 90 minutes with each family. During this visit the research team will provide additional written and verbal information about the study, and will allow participants to ask any questions about the study. The purpose of the study will be explained, as well as the procedures participants will be asked to complete. Participants will be informed that they can withdraw from any aspect of the home visits, and from the overall study at any time. The randomisation process will also be explained, so participants are aware that they will be randomly allocated to one of the two groups.

11.3. Blinding

Researchers assessing study outcomes will be blinded to randomised allocation. Participants (i.e. parents and their children) will be informed of their randomised allocation. In the event of a severe adverse event the Chief Investigator will be notified and may be informed of the randomised allocation.

11.4. Randomisation

Randomisation lists (one per site) will be prepared by a statistician using 1:1 allocation (ViPP intervention vs treatment as usual) and appropriate block sizes and uploaded on to InForm (the study electronic data capture system) prior to the start of the study. Eligible subjects will be allocated online to the next available treatment code in the appropriate randomisation list.

Randomisation will be stratified by treatment centre and by willingness and availability of both parents to be involved (versus one only).

11.5. Follow-up visits

Follow-up assessments will be undertaken in the family home by research assistants who are blind to treatment allocation, at a time convenient for the family.

Participants will be contacted by the research team prior to the visit time or the first post-treatment assessment and they will be sent a thank you note (via post or email) following this assessment. The thank you note will include a reminder of the later follow up arrangements and a request to contact the research team if their contact details change.

Participants will then be contacted one month prior to the planned final follow up at 2 years post randomisation to arrange a suitable time and place for the assessment. Where parents incur travel costs, these will be reimbursed.

11.6. Assessment Visit Schedule

	Baseline	5 month f/u	24 month f/u
**Visit**	**1**	**2**	**3**
Day/Week/Month		Month 5 post randomisation (+/- 3 weeks)	Month 24 post randomisation (+/- 3 months)
Informed consent	X		
Inclusion & exclusion criteria	X		
Demographics and medical history	X		
AUDIT-C	X	X	
Randomisation	X		
SDQ	X	X	X
CBCL	X	X	X
GAD-7	X	X	X
Pre-PACS interview	X	X	X
PHQ-9	X	X	X
Revised Dyadic Adjustment Scale	X	X	X
Parenting Scale	X	X	X
CA-SUS	X	X	X
Parent-Child interactions	X	X	X
Parental involvement questionnaire			X
Child temper tantrum questionnaire			X
Child emotion regulation (AITB; Lab-TAB)			X
Child executive function (MEFS)			X
Child prosocial behaviour			X
Child genetic variance			X
Feedback questionnaire		X	
Serious adverse events	X	X	X

11.7. ViPP-SD Visit Schedule

	VIPP Intervention Schedule[a]					
**Visit**	**1**	**2**	**3**	**4**	**5**	**6**
Day/Week/Month	Day 14-28 (+/- 7 days) post randomisation	Visit 1 plus 14 days (+/-7 d)	Visit 2 plus 14 days (+/-7 d)	Visit 3 plus 14 days (+/-7 d)	Visit 4 plus 21 days (+/-7 d)	Visit 5 plus 21 days (+/-7 d)

Note. [a]*Visit schedule is a guide for optimal treatment delivery, variation is expected given the pragmatic context of the trial.*

11.8. Measures

11.8.1. Pre-PACS

The primary outcome measure will be an assessment of the severity of the child's behavioural problems using a structured investigator-led interview of a parent or caregiver (the Preschool-PACS), at five and twenty four months after randomisation (the child will be aged 3-5 years at this later assessment point).Where two parents/caregivers are participating in the trial the Pre-PACS will be completed by the parent who identifies as being the primary caregiver. The Pre-PACS is a semi-structured assessment of child behaviour problems, administered by trained interviewers, which yields a score for behavioural problems based on investigator judgement. To determine pre-PACS scores, caregivers are asked to recall and describe detailed examples of their child's behaviour over the last week in a range of settings (e.g., in the home, with peers, and in public settings such as the supermarket). The parent is also asked about how representative the behaviour is of the past 4 months (to ensure the example is typical and characteristic of the child). The interviewer then rates the severity and frequency of the symptoms on the basis of their professional/clinical judgement and written definitions and thresholds of the behaviours, validated according to clinical practice. Symptoms are rated for frequency and severity on two subscales, one measuring ADHD/Hyperkinesis, and the other measuring conduct problems and antisocial behaviours.

The Pre-PACS has high inter-rater reliability and good construct validity, and has been used in previous clinical trials (e.g., 11-14). Interviewers will be blind to allocation. Semi-structured interviews are the gold-standard measure for most psychiatric disorders. They are more objective as they use investigator-based criteria for scoring symptoms, and are thus less prone to parental biases, which are seen when using parent-reported questionnaires. All PPACS interviews will be recorded for reliability purposes. Recordings will be assessed periodically to avoid drift and ensure that the measure remains robust to rater and respondent bias.

11.8.2. CBCL

The Child Behavior Checklist (45), which is a robust and widely-used questionnaire, will be used as child behaviour is the main outcome of interest.

Each question asks about a specific behaviour and a score is given (0 = Not True, 1 = Somewhat or Sometimes True, 2 = Very True or Often True). For behavioural problems (externalising problems) it yields an overall score, as well as specific subscales for attention problems and aggressive behaviours. The CBCL is a well-validated questionnaire, which has been extensively used in previous substantive clinical trials. The CBCL will be completed by one or two parents/caregivers depending on their participation in the trial.

11.8.3. SDQ

The SDQ (Strengths and Difficulties Questionnaire) is a robust and reliable measure of child behaviour. The SDQ will be used as a screening questionnaire, where those scoring in the top 20% on population norms will be eligible to take part in the trial.

The questionnaire is made up of 25 items that make up 5 subscales (5 items per subscale). The subscales include conduct problems, hyperactivity-inattention, emotional symptoms, peer problems and pro-social behaviour. Each question asks about a specific behaviour and is rated as 0 = Not True,1 = Somewhat True or 2 = Certainly True (items 1,4,9,17 and 20 make up the pro-social behaviour score, which is reverse scored). The combined scores of the subscales (not including the pro-social behaviour subscale), can be combined to generate an overall difficulties score which can range from 0-40. Higher scores in the overall difficulties scale indicate increased difficulties. The scoring for the pro-social behaviour scores are reversed, and range of scores between 0-10. The lower scores on this subscale indicate increased difficulties. The SDQ will be completed by one or two parents/caregivers depending on their participation in the trial, in addition to a nursery teacher or carer who knows the child well. Almost all of the children will be in some form of non-parental care or schooling by the age of 3-5 years (the age at final follow-up) and previous response rates in studies from nursery and preschool teachers have been excellent (97% in the FCCC study15). This will provide an independent report of the child's behaviour, in addition to the semi-structured interview and questionnaire measures completed by parents.

11.8.4. RDAS

The Revised Dyadic Adjustment Scale (RDAS) is a reliable and valid measure of relationship adjustment. This 14 item scale consists of three subscales: dyadic consensus, dyadic satisfaction and dyadic cohesion. A total DAS score is obtained by summing all items of the questionnaire. Scores range from 0 to 69, where higher scores indicate greater relationship satisfaction, and lower scores greater relationship distress. The RDAS will

be completed by one or two parents/caregivers depending on their participation in the trial and relationship status.

11.8.5. Patient Health Questionnaire 9

The Patient Health Questionnaire 9 is a widely used and reliable measure of depression severity. The measure is made up of nine statements, each corresponding to one of the 9 DSM-IV criteria for depression. Each statement is scored on the frequency the responder has experienced each problem over the past two weeks. Scores range from Not at all = 0, Several days = 1, More than half the days = 2 or Nearly every day = 3, and a total score is obtained by summing all items of the questionnaire. Scores range from 0-27, with higher scores indicating more severe depression. The PHQ-9 will be completed by one or two parents/caregivers depending on their participation in the trial.

11.8.6. Parenting Scale

The Parenting Scale is a reliable and valid measure of dysfunctional discipline practices in parents. This will be assessed on each parent separately (when both parents are available). The Parenting Scale will be completed by one or two parents/caregivers depending on their participation in the trial.

11.8.7. CA-SUS

A modified version of the Child and Adolescent Service Use Schedule (CA-SUS) will be used in the trial. The CA-SUS has been developed and successfully employed in previous evaluations with young people (e.g. 48-49), including pre-school children (50). Where two parents/caregivers are participating in the trial the PPACS will be completed by the parent who identifies as being the primary caregiver.

11.8.8. GAD-7

A seven item anxiety disorder questionnaire that has been extensively used in research as a general measure of anxiety in adults. The GAD-7 will be completed by one or two parents/caregivers depending on their participation in the trial.

11.8.9. AUDIT-C

The Alcohol Use Disorders Identification Test - Consumption (AUDIT-C) is an abbreviated version of the original AUDIT (a 10-item screening questionnaire used to detect and identify signs of hazardous, harmful, or dependent drinking). The AUDIT-C, consisting of questions 1-3 of the AUDIT, solely encompassing the consumption items, and as such will be used to obtain information regarding parents' alcohol consumption. Individual question scores range from 0-4, making the overall possible score total range from 0-12. An overall score of 5 or above on the AUDIT-C indicates increasing or higher risk drinking.

11.8.10. Parental sensitivity

Parental sensitivity will be rated based on video recorded parent-child interactions, using a standardised rating scale, by raters blinded to group allocation.

11.8.11. Parental involvement

Parental involvement will be measured using a brief reliable, and valid questionnaire (51) which asks parents/caregivers about the frequency with which they engage in several caregiving and play activities with their child. The parental involvement scale will be completed by one or two parents/caregivers depending on their participation in the trial.

11.8.12. Temper tantrum screen

A reliable, and valid three-item questionnaire assessing the frequency and intensity of temper tantrums in young children will be used (Egger, 2016). This measure has been shown to have strong predictive validity for psychopathology in childhood. Where two parents/caregivers are participating in the trial, the questionnaire will be completed by the parent who identifies as being the primary caregiver.

11.8.13. Emotion regulation

Emotion Regulation will be measured using the Attractive Toy in a Transparent Box task, taken from the Laboratory Temperament Assessment Battery (Lab-TAB) (52). This task is designed to allow for the analysis of the child's emotion regulation and response to blocked goals. During the four minute task the child is asked to retrieve a toy from a locked box using a ring of incorrect keys. The child is allowed to keep the toy following the task. Should the child give any verbal or nonverbal cues during the activity that they wish to end the task then the researcher will do so. Recordings of the activity will be coded for emotion regulation using a standardised rating scale, by raters blinded to group allocation

11.8.14. Executive function

The Minnesota Executive Function Scale (MEFS) (53) will be used to carry out an assessment of executive function skill level. The MEFS has high content validity, as well as high convergent validity with other EF assessments. The measure is delivered using a tablet and is an adaptive virtual card-sorting task which takes approximately 4-minutes for the child to complete.

11.8.15. Prosocial behaviour

A 'dolls play' task where a short play interaction between child and researcher is recorded. This task is designed to provide an index of the child's prosocial behaviours. Recordings will be coded using a standardised rating scale, by raters blinded to group allocation.

11.8.16. Genetic variance

Where participants consent to take part in the genetic aspect of the trial we will collect a buccal (mouth) swab from the child. This will be used to obtain cells to conduct an analysis of genes related to child development and behaviour, including key candidate genes such as BDNF, FKBP5, NR3C1, DRD4, SLC6A4 and 5-HTTLPR. This will allow us to determine how child behaviour relates to the methylation of certain genes and whether the effects of VIPP-SD are moderated by children's genotype. This involves a non-invasive procedure undertaken by trained researchers where a swab is gently rubbed on the inside of the child's cheek to extract cells for genetic testing. Data will be collected, transferred, analysed, and stored in accordance with the Human Tissue Act. Following analysis samples will be stored in the Imperial College tissue bank.

11.8.17. Feedback questionnaire

A brief feedback questionnaire will be given to parents at the 5-month follow-up visit. This is closely based on those previously used in ViPP studies in the United Kingdom. Questions explore participant satisfaction and experience of the intervention's helpfulness, enjoyableness, relevance, and format.

12. Safety Reporting

12.1. Adverse Event (AE)

An AE is any untoward medical occurrence which does not necessarily have a causal relationship with the trial treatment. An AE can therefore be any unfavourable and unintended sign (including an abnormal laboratory finding), symptom, or disease temporally associated with the trial treatment, whether or not considered related to the treatment.

12.2. Severity of Adverse Events

Severity of AEs will be assessed according to the following definitions:

Mild: Awareness of event but easily tolerated
Moderate: Discomfort enough to cause some interference with usual activity
Severe: Inability to carry out usual activity, including play for infants and children

12.3. Causality of Adverse Events

Causality of AEs, i.e. relationship to the trial treatment, will be assessed according to the following definitions:

Unrelated	No evidence of any causal relationship

Unlikely	There is little evidence to suggest there is a causal relationship (e.g. the event did not occur within a reasonable time after administration of the treatment). There is another reasonable explanation for the event (e.g. the participant’s clinical condition, other concomitant treatment).
Possible	There is some evidence to suggest a causal relationship (e.g. because the event occurs within a reasonable time after trial treatment). However, the influence of other factors may have contributed to the event (e.g. the participant’s clinical condition, other concomitant treatments).
Probable	There is evidence to suggest a causal relationship and the influence of other factors is unlikely.
Definite	There is clear evidence to suggest a causal relationship and other possible contributing factors can be ruled out.

12.4. Serious Adverse Events (SAE)

12.4.1. Definition of SAE

An SAE is defined as any adverse event that:

- Results in death
- Is life-threatening*
- Requires hospitalisation or prolongation of existing inpatient’s hospitalisation**
- Results in persistent or significant disability or incapacity
- *Is a congenital abnormality or birth defect****

* “Life-threatening” in the definition of “serious” refers to an event in which the participant was at risk of death at the time of the event; it does not refer to an event which hypothetically might have caused death if it were more severe.

** “Hospitalisation” means any unexpected admission to a hospital department. It does not apply to scheduled admissions that were planned before study inclusion or visits to an accident and emergency department (without admission).

*** “Congenital abnormality or birth defect” will not be applicable for this trial as all participants will be children aged 12 – 36 months.

Medical judgement should be exercised in deciding whether an adverse event/reaction is serious in other situations. Important adverse events/reactions that are not immediately life-threatening, or do not result in death or hospitalisation but may jeopardise a participant, or may require intervention to prevent one of the other outcomes listed in the definition above should also be considered serious.

12.4.2. Reporting of SAEs

Due to the low risk nature of this study, AEs that do not meet the above Seriousness criteria will not be collected during the study.

Rapid reporting of all SAEs occurring during the study must be performed as detailed in SAE reporting instructions. SAEs will be reported via the eCRF within 24 hours of becoming aware of the event. All reported SAEs will be reviewed by the Chief Investigator (or designee) within 2 working days of receiving notification of the SAE report. The SAE review will be recorded on the eCRF.

SAEs will be followed up until they are resolved.

If the investigator becomes aware of safety information that appears to be related to the treatment, involving a participant who participated in the study, even after an individual participant has completed the study, this should be reported to the Sponsor.

12.5 Definition of a Serious Adverse Reaction (SAR)

A SAR is defined as a SAE that is judged to be related to the trial treatment.

12.6 Definition of Unexpected and Related Serious Adverse Events

A Related and Unexpected Serious Adverse Events is an Adverse Event that is classed as serious, is suspected to be caused by the trial treatment and is unexpected i.e. not listed as an ‘expected SAE’ in this protocol.

12.6.1 Reporting of Related and Unexpected Serious Adverse Events

All Related and Unexpected Serious Adverse Events will be notified to the Research Ethics Committee (REC) and the Sponsor within 15 days of becoming aware of the event.

Follow up of participants who have experienced a Related and Unexpected Serious Adverse Event should continue until recovery is complete or the condition has stabilised.

12.7 Annual reporting of SAEs

Annual safety reporting will be included in the annual progress report sent to the REC, on the anniversary of Ethics approval each year.

13. STATISTICAL ANALYSES

13.1. Sample Size and power considerations

The total sample size will be 300 participants.

If losses to follow-up are 20%, this leaves 120 participants per group with follow-up data. We would then have 80% and 90% power to detect standardised effect sizes of 0.36 and 0.42 respectively, at the 5% significance level. In addition, we have stated that our analysis will adjust for baseline behavioural score, research centre and age of child, which will increase power, probably to over 90% for the 0.36 effect size (since such adjustment will reduce the residual error variance in our model). (Kahan and colleagues (2014) found that covariate adjustment for 1 to 4 variables in trials increased power from 80% to a median of 93% power in their sample of 12 outcomes assessed across 8 studies.)

We have conservatively allowed for a potential drop-out rate of 20% because of the longer follow-up time in the proposed study, even though previous intervention studies detailed below have maintained retention rates of over 90% at follow-up.

The pooled effect size for all randomised controlled trials to date that have used the same video feedback intervention (ViPP) is 0.46 (Bakermans-Kranenburg, 2013, personal correspondence). Other relevant literature for interventions for behavioural problems, predominantly in slightly older children, yield higher effect size estimates. In the systematic reviews undertaken for the most recent NICE guidance on conduct disorders in children and young people (5), the pooled effects for parent-focussed interventions yielded estimated effect sizes of 0.69 standard deviations for researcher-rated outcome (the main outcome measure in the proposed study), and 0.54 for parent-rated outcome. In a study of the Incredible Years programme in 2-9 year olds (6) effect sizes ranged from 0.48-0.78. In the SPOKES trial (7) of intervention for parents of 6 year olds, the effect size for behavioural outcomes was 0.52 SD difference between the treatment and control group. Where parenting programmes have been rolled out across the UK (8-9) similar effect sizes have been found, albeit in non-randomised designs (effect sizes ranged from 0.44-0.71).

13.2. Data Analysis

The primary analysis will be by intention to treat (ITT). Histograms and box-plots will be used to assess the distributional assumptions and to check for possible outliers. Log transformations will be applied, where appropriate, in order to render the outcomes distributions closer to the Normal. Bootstrap techniques will be used if this does not achieve reasonable normality, to the extent that this may influence the properties of the regression analysis. The relationship between the outcomes and other variables will be explored graphically, using scatter plots and box-plots. Continuous variables that follow an approximately Normal distribution will be summarised using the mean and standard deviations. Skewed variables will be summarised using the median and inter-quartile range. Categorical variables (binary and ordinal and multinomial) will be presented in terms of frequencies and percentages.

Before starting the data analysis, the level, pattern and likely causes of the missingness in the baseline variables and outcomes will be investigated by forming appropriate tables. This information will be used to determine

whether the level and type of missing data has the potential to introduce bias into the analysis results or reduce substantially the precision of estimates for the proposed statistical methods. The primary outcome, externalizing behaviour (Pre-PACS), will be analysed at follow-up using linear regression analysis (after checking regression assumptions), adjusting for treatment centre, parental willingness to participate (one or two parents), for infant's baseline behaviour and for infant's age at randomisation. Sensitivity analyses will be undertaken, based on assuming that missing outcomes are the worst possible, or the best possible, in different randomisation groups. If these show that conclusions may differ based on missing values, then supplementary multiple imputation for missing values will be undertaken. These analyses will account for results of any losses to follow-up insofar as they pertain to differences in measured variables (i.e. under the assumption of missing at random). This will enable us to effectively incorporate information gleaned from earlier follow-up times when the final follow-up outcome is absent. This will be done by incorporating outcomes at earlier time points into the predictive model for the multiple imputation of the outcome at 2 year follow-up. Secondary outcome variables will be analysed similarly.

Categorical outcome variables will be presented by treatment group, and compared using logistic/ ordered logistic regression adjusted as per linear regression above.

A detailed statistical analysis plan will be prepared and signed off prior to any interim analyses.

13.3. Economic analysis

13.3.1. Short-term cost-effectiveness

Short-term assessment of cost-effectiveness will take the NHS/Personal Social Services perspective preferred by NICE (23), and will include all hospital and community based health and social services provided for the child over the course of the trial. Data will be recorded in interview with parents at baseline, end of intervention and follow-up assessments using a modified version of the Child and Adolescent Service Use Schedule (CA-SUS), developed and successfully employed in previous evaluations with young people (e.g. 48-49), including pre-school children (50). Data on intervention contacts and other resources will be collected directly from health visitor records and indirect time (time spent on preparation, supervision, administration, travel etc) will be estimated using questionnaires completed by each health visitor delivering the intervention. National unit costs will be applied to all services (54-55), with the exception of the ViPP intervention, which will be costed using a micro-costing approach (56).

Two short-term economic evaluations will be undertaken:

i) cost-effectiveness analysis using the primary outcome measure of the trial (Pre-PACS)
ii) cost-consequences analysis, outlining the costs alongside all secondary outcome measures in order to explore potential economic impacts of the intervention more broadly. No method of direct estimation of health-related quality of life, and thus quality adjusted life years (QALYs), currently exists for infants and pre-school children, so it is not possible to undertake a cost-utility analysis at this stage. However, the feasibility of using modelling to explore longer-term cost-utility will be explored, as described below.

For the cost-effectiveness analysis, incremental cost-effectiveness ratios will be reported and uncertainty explored using cost-effectiveness acceptability curves (57-59).

13.3.2. Long-term cost-effectiveness

The economic implications of behavioural problems are long-term in nature, with childhood behaviour problems being linked to later delinquency and criminality and affecting future mental health status and education and employment outcomes (60-61). Longer term outcomes will be explored using decision analytic modelling, following methods applied in similar research (62).

Data from the trial will be supplemented with data from a systematic literature review, which takes a broader perspective, additionally including education and criminal justice sector resources, the cost of criminal activity and productivity losses. In terms of outcomes, where data allow, effectiveness estimates in the trial will be linked to estimates of health-related quality of life scores, to support a cost-utility analysis. The Strengths and Difficulties Questionnaire (SDQ) (21) will be used for this purpose, as there are known datasets containing SDQ and utility scores (e.g. 22). The SDQ, completed by parents, is suitable for children aged 3 years and upwards, the age of the proposed population at final follow-up. However, the systematic review may highlight alternative outcomes that can be mapped onto utility scores, so this will be finalised during the course of the study.

Decision analysis will be used to model data from the proposed trial plus existing data on costs, outcomes and probabilities from published studies (24, 63). The most suitable modelling framework in which to carry out the

analysis will be selected, dependent upon the results of the proposed study. In cases where individuals can be regarded as independent and interaction between them is not an issue in terms of the course or progression of an illness, as is the case in the current population, either a decision tree or a Markov model is appropriate (64). Decision trees are limited by their fairly simplistic representation of reality and they can often become unwieldy as attempts are made to make them sufficiently complex to model real-world scenarios. A Markov model may provide a useful alternative since they are better able to deal with more complicated structures and are often used when costs and outcomes need to be considered over longer periods of time. The final choice between these two frameworks will be informed by the findings of the naturalistic study.

The cost-effectiveness of the ViPP versus control groups will be analysed using incremental analysis and probabilistic sensitivity analysis. It is necessary for models to build in uncertainty estimates for the probability, cost and outcome parameters used. In this model it is likely that variability, heterogeneity and uncertainty will be important and will therefore need to be incorporated. Because many of the model parameters will be based on real data from the proposed RCT study, it will be possible to use regression models and appropriate assumptions regarding the statistical distribution of the data to handle the uncertainty (24). The model will initially be run over two years, in line with the data to be collected in the trial. However, secondary analysis will explore longer time periods, dependent on data availability.

14. Patient and public involvement (PPI)

The views of parents of young children with behavioural problems have been considered during the development of this protocol. Parents will play a significant role in helping to conduct the study, monitor study progress and disseminate study findings.

In previous trials of the ViPP-SD intervention, parents fed back that it would be useful to have support earlier on in their children’s lives, before behavioural problems become established. Repeated feedback from mothers has also suggested that they are keen for their partners to be involved in interventions and that fathers appreciate involvement. Two participants from the previous pilot studies will provide ongoing input into the trial.

Feedback from participants and service users has influenced the protocol in the following ways:

i) Plan to take pragmatic approach to the delivery of the intervention, focusing on ensuring engagement with the primary caregiver, but also actively trying to involve both parents/carers if possible;
ii) assessment and therapy sessions will be held in participants’ homes and will be flexibly timed, offering evening and weekend sessions where necessary;
iii) the intervention format is more flexible so that sessions can be held with an individual parent or with parents/carers together.

A Parent Advisory Group (PAG) will be set-up to oversee study progress throughout the duration of the trial. There will also be two service users on the Trial Steering Committee. Members of the PAG will be asked to comment on participant information before the start of the study and will also be sent a draft version of the full study report, summary reports of the study findings for participants, and all other aspects of the dissemination strategy.

15. REGULATORY, ETHICAL AND LEGAL ISSUES

The study will be conducted in accordance with the Declaration of Helsinki, the Data Protection Act and the guidelines laid down by the International Conference on Harmonisation for Good Clinical Practice (ICH GCP E6 guidelines).

15.1. Research Ethics Committee (REC) Approval

Approval from a multi-centre Research Ethics Committee (REC) will be obtained prior to the start of the trial. REC approval will include the trial protocol, parent information sheet and consent form, questionnaires, interviews, any other written information that will be provided to the participants and any advertisements that will be used during the study.

15.2. Approval of Amendments

Any amendments to the protocol and information provided to participants will be submitted to the Sponsor and the REC for approval prior to implementation. An assessment of whether the amendment is substantial or non-substantial will be made prior to submitting the amendment for review. Substantial amendments may only be implemented after written REC approval has been obtained whereas non-substantial amendments can be implemented without written approval from the REC.

Amendments that are intended to eliminate an apparent immediate hazard to participants may be implemented prior to receiving Sponsor or REC approval. However, in this case, approval must be obtained as soon as possible after implementation.

15.3. Addition onto trial register

The trial protocol will be registered on clinicaltrials.gov in accordance with the International Committee of Medical Journal editors (ICMJE) requirements. Any protocol amendments will also be registered there.

15.4. Annual Progress Reports

A progress report will be submitted to the REC on an annual basis, on the anniversary of REC approval. The progress report will also include details of safety information.

15.5. End of Trial Notification

A notification of the end of the trial will be submitted to the REC within 90 days of the final follow-up visit taking place

15.6. NHS Health Research Authority Study Approval

Approval for the study to be conducted within NHS sites will be obtained from the NHS Health Research Authority.

15.7. Informed Consent

All adult research participants (parents of the children in the study) will sign and date an Informed Consent Form (ICF) before any trial specific procedures are performed. Participants will be asked to provide written consent twice: written/electronic consent to complete the screening questionnaire and, if eligible, written consent to participate in the full study.

Health visitors will recruit families to take part in the study at the routine 12 and 24-month health reviews. Clinicians in other settings will recruit families when they are referred for support. Members of the research team and clinical research network support staff will also support recruit families on site in health visiting services.

Potential participants will be initially approached to complete a screening questionnaire. This screening questionnaire will be presented to participants as a pack, together with an information sheet and consent form regarding taking part in the screening stage of the recruitment. The screening questionnaire pack will either be sent to participants as an enclosure in a standard letter being sent regarding the service (e.g. an invitation to attend a 12 or 24 month health review), a separate mailshot if preferred by the service, or handed to potential participants when they attend the service.

Those participants that received the screening questionnaire in the post will be invited (via written information in the pack) to return it to their healthcare profession at their subsequent appointment. The written information will also explain that they will have ample time to discuss any questions they have about the screening stage of the study at their next appointment, and withdraw their consent at any time. It will be made clear to participants that they are providing consent to complete the screening questionnaire only, and not the full study.

Those participants that are given the screening questionnaire pack in person when they attend the service, and then go on to consent to take part in the screening, will have the opportunity to complete the SDQ and consent form whilst at the service, or they will be asked to complete both the questionnaire and consent form to return by post, in person when next using the service, or electronically using the link provided in the information sheet.

Those parents that see recruitment information or have been signposted to the study (e.g. via posters and leaflets displayed in health settings, or on internet advertisements) and therefore make contact directly with the research team will be provided with the same screening information pack to complete and return, if they so wish. Members of the research team or clinical research network support staff will also visit community centres such as children’s centres and one o’clock clubs (e.g., during play, activity, information, and training sessions), with permission from management, to disseminate screening packs to families and where appropriate support families in completing the screening questionnaire, in line with the procedures outlined above.

The researcher involved in consenting participants to the study will encourage them to spend as much time as they want asking questions about the study and considering whether they want to take part. In all instances potential participants will have at least 24 hours before deciding whether they wish to take part in the study.

Following screening, participants who score in the top 20% of population norms will be contacted by a member of the research team to see if they would like to participate in the full study. If verbal consent is given, a date will be arranged for one or two members of the research team to visit them at home to complete the first assessment visit. Written consent to the trial will be taken at the initial assessment home visit with families. Participants will already have received study information by post or email and will have had the opportunity to ask the study team questions over the phone. At the visit a trained research assistant will take the participants through each of the clauses on the consent form and participants will record their written consent. A copy of the information sheet and ICF will be given to the parents for their records and a further copy stored in the participant file.

Study participants will be asked to give up their time to take part in study assessments and to complete study questionnaires. Baseline assessment takes approximately 90 minutes, with a shorter period for the follow up assessments. All participants will be offered an honorarium following the assessments.

The process of obtaining informed consent will be conducted in accordance with the requirements of Research Ethics Committee guidance, the Declaration of Helsinki and Good Clinical Practice.

15.8. Contact with General Practitioner and Health visitor

It is the investigator's responsibility to inform the child's General Practitioner and Health Visitor by letter that the child is taking part in the study provided the child's parent agrees to this, and information to this effect is included in the Participant Information Sheet and Informed Consent Form. A copy of the letters should be filed in the Investigator Site File.

15.9. Patient Confidentiality

The investigator must ensure that the participant's privacy is maintained. On the eCRF or other documents submitted to the Sponsors, participants will be identified by a trial ID number only. Documents that are not submitted to the Sponsor (e.g., signed informed consent form) should be kept in a strictly confidential file by the investigator.

All audiovisual recordings made by the research team will be immediately uploaded after each session via a secure digital platform that will be supported by the Sponsor, Imperial College London. These audiovisual recordings will be backed up on an external hard drive that will be password protected and accessible only to specific members of the research team. The audiovisual recordings will be stored anonymously according to each family's study ID.

All temporary video stored on video cameras will be deleted and permanently removed immediately after each session, once the video has been uploaded to the secure digital platform.

The investigator shall permit direct access to participants' records and source document for the purposes of monitoring, auditing, or inspection by the Sponsor, authorised representatives of the Sponsor and the REC.

16. End of Trial

The end of the trial will be defined as the last data capture for the last participant recruited.

17. ADMINISTRATIVE MATTERS

17.1. Source Data

Trial therapist and Research assistant records including paper questionnaires and measures completed during study assessments.

17.2. Language

eCRFs will be in English. All written material to be used by participants must use vocabulary that is clearly understood.

17.3. Data collection and management

Data will be collected on an electronic Case Report Form (eCRF) developed using the InForm system. The eCRF will include the randomisation system and database. This will be a web-based eCRF comprising a full GCP-compliant audit trail, stored on a secure server. Access will be restricted to trained staff with unique password-protected accounts. Identifiable data will not be recorded in the eCRF and participants will be

identified by a unique trial ID only. Instructions for completion of the eCRF will be provided in a separate eCRF manual.

Hard copies of data sheets linking the participant identification number to the person's contact details will be kept securely in the Investigator Site File, in a locked filing cabinet in a locked office, accessible only to key research team members.

17.4. Study Documentation and Data Storage

The investigator will retain essential documents until notified by the Sponsor, and at least for ten years after study completion, in accordance with Sponsor requirements. Participant files and other source data (including copies of protocols, questionnaires, original reports of test results, correspondence, records of informed consent, and other documents pertaining to the conduct of the study) will be kept for the maximum period of time permitted by the institution. Documents will be stored in such a way that they can be accessed/data retrieved at a later date. Consideration will be given to security and environmental risks.

No study document will be destroyed without prior written agreement between the Sponsor and the investigator. Should the investigator wish to assign the study records to another party or move them to another location, written agreement will be obtained from the Sponsor.

All audiovisual recordings made by the research team will be immediately uploaded after each session via a secure digital platform that will be supported by the Sponsor, Imperial College London. These audiovisual recordings will be backed up on an external hard drive that will be password protected and accessible only to specific members of the research team. The audiovisual recordings will be stored anonymously according to each family's study ID.

All temporary video stored on video cameras will be deleted and permanently removed immediately after each session, once the video has been uploaded to the secure digital platform.

17.5. Study Management Structure

17.6. Trial Steering Committee (TSC)

A Trial Steering Committee (TSC) will be established to oversee the conduct of the study. TSC will comprise the lead investigators, an independent chair, additional independent members and two user representatives. The TSC will meet prior to the start of the study and every six months during recruitment and annually during follow-up, or as required throughout the duration of the trial. Reports from each meeting will be submitted to the trial funder.

17.7. Data Monitoring and Ethics Committee (DMEC)

An independent Data Monitoring and Ethics Committee (DMEC) will be established to oversee safety of the trial. The DMEC will review SAE reports and key data as required. **The DMEC will develop, in agreement with the investigators and TSC, a charter outlining their responsibilities, planned interim analyses and operational details. The DMEC will meet prior to the start of the trial to agree the charter.**

17.8. Project Management Group (PMG)

The Project Management group will be responsible for overseeing management of the study and operational issues. The PMG will meet every 2 months during the set-up phase of the trial and every 6 months thereafter. Membership will include the Chief Investigator, key investigators, the Trial Manager and Trial Statistician.

17.9. Patient Advisory Group (PAG)

At establishment of the study we will set up a Parent Advisory Group of service users drawn from clinical services in London, including two previous research participants who will also serve on the Trial Steering Group. The Parent Advisory Group will help us develop material for publicising the study and design the Patient Information Sheet that we would use. The clinical service in Westminster, where the Chief Investigator is a consultant, already has well established parent advisory groups for a range of different advisory functions.

17.10. Target organisations

NHS services for young children and linked children’s centres in the UK.

17.11. Monitoring

The study will be monitored periodically by the Trial Manager or Trial Monitor to assess the progress of the study, verify adherence to the protocol, ICTU SOPs, ICH GCP E6 guidelines and to review the completeness, accuracy and consistency of the data.

Monitoring procedures and requirements will be documented in a Monitoring Plan.

Therapists will report on fidelity in terms of the delivery of key components of the treatment as well as reporting on global adherence to the manual. Compliance will be assessed by the clinical supervisor in supervisory sessions. In addition, a random proportion of the audio recordings will be assessed by an independent assessor who is trained in the intervention.

17.12. Quality Control and Quality Assurance

Quality Control and Quality Assurance will be performed according to ICTU procedures. The ICTU QA Manager will conduct a risk assessment prior to the start of the study to assign a risk category to the trial. The monitoring plan will be developed in accordance with the outcome of the Risk Assessment. The study may be audited by a Quality Assurance representative of the Sponsor or ICTU. All necessary data and documents will be made available for inspection.

17.13. Publication policy

The results from the trial will be submitted for publication in a peer-reviewed journal irrespective of the outcome. The Trial Steering Committee will be responsible for approval of the main manuscript prior to submission for publication. At the end of the study, children's parents will be able to request a copy of the results of the study from the investigator at that site.

Authorship of presentations and reports related to the study will be in the name of the collaborative group. The final follow-up study results paper will name local co-ordinators as well as those involved in central co-ordination and trial management.

REFERENCES

1. Juffer, F., Bakermans-Kranenburg, M. and Van IJzendoorn, M. 2008. *Manual VIPP-SD. Video-feedback Intervention to promote Positive Parenting and Sensitive Discipline*. Leiden: Centre for Child and Family Studies, Leiden University.
2. Owen-Jones, E., Bekkers, M-J., Butler, C., Cannings-John, R., Channon, S., Hood, K., et al. 2013. The effectiveness and cost-effectiveness of the Family Nurse Partnership home visiting programme for first time teenage mothers in England: a protocol for the Building Blocks randomised controlled trial. *BMC Pediatrics* 2013, **13,** 114.
3. Barnes, J., Ball, M., Meadows, P., Howden, B., Jackson, A., Henderson, J. and Niven, L. 2011. The Family-Nurse Partnership Programme in England: Wave 1 Implementation in toddlerhood and a comparison between Waves 1 and 2a implementation in pregnancy and infancy. London, Department of Health.
4. Ramchandani, P.G., Psychogiou, L., Vlachos, H., Iles, J., Sethna, V., Netsi, E. and Lodder, A. 2011. Paternal depression: an examination of its links with father, child and family functioning in the postnatal period. *Depression and Anxiety*. **28**, 1091-4269
5. National Institute for Health and Care Excellence. 2013. Conduct Disorders in Children and Young People. London: NICE.
6. Gardner, F., Burton, J., Klimes, I. 2006. Randomised controlled trial of a parenting intervention in the voluntary sector for reducing child conduct problems: Outcomes and mechanisms of change. *Journal of Child Psychology and Psychiatry*. **47,** 1123–1132.
7. Scott, S., Sylva, K., Doolan, M., Price, J., Jacobs, B., Crook, S. and Landau, S. 2010. Randomised controlled trial of parent groups for child antisocial behaviour targeting multiple risk factors: the SPOKES project. *Journal of Child Psychology and Psychiatry*. **15**, 48-57.
8. Lindsay, G., Strand, S. and Davis, H. 2011. A comparison of the effectiveness of three parenting programmes in improving parenting skills, parent mental-well being and children's behaviour when implemented on a large scale in community settings in 18 English local authorities: the parenting early intervention pathfinder (PEIP). *BMC Public Health*. **11**, 962.
9. Lindsay, G. and Strand, S. 2013. Evaluation of the national roll-out of parenting programmes across England: the parenting early intervention programme (PEIP). *BMC Public Health*. **13**(1), 972.
10. Stolk, M., Mesman, J., van Zeijl, J., Alink, l., Bakermans-Kranenburg, M., van IJzendoorn, M., et al. 2008. Early parenting intervention aimed at maternal sensitivity and discipline: A process evaluation. *Journal of Community Psychology*, **36** (6), 780–797.
11. Scott, S., Spender, Q., Doolan, M., Jacobs, B., Aspland, H. and Webster-Stratton, C. 2001. Multicentre controlled trial of parenting groups for childhood antisocial behaviour in clinical practice. *BMJ*. **323**, 1-7.
12. Sonuga-Barke, E., Daley, D., Thompson, M., Laver-Bradbury, C. and Weeks, A. 2001. Parent-based therapies for preschool ADHD: A randomized, controlled trial with a community sample. *Journal of the American Academy of Child and Adolescent Psychiatry*. **40**(4), 402-8.
13. Sonuga-Barke, E., Thompson, M., Daley, D. and Laver-Bradbury, C. 2004. Parent training for ADHD: Is it as effective when delivered as routine rather than as specialist care? *British Journal of Clinical Psychology*. **43**(4), 449-57.
14. Taylor, E., Schachar, R., Thorley, G. and Wieselberg, M. 1986. Conduct disorder and hyperactivity: I. Separation of hyperactivity and antisocial conduct in British child psychiatric patients. *British Journal of Psychiatry*. **149**, 760-7.
15. Stein, A., Malmberg, L.E., Leach, P., Barnes, J., Sylva, K., and the FCCC Team. 2013. The influence of different forms of early childcare on children's emotional and behavioural development at school entry. *Child: Care, Health and Development*. **39**(5), 676-87.
16. Juffer, F., Bakermans-Kranenburg, M. and Van Ijzendoorn M. 2005. The importance of parenting in the development of disorganized attachment: Evidence from a preventive intervention study in adoptive families. *Journal of Child Psychology and Psychiatry*. **46**, 263-74.
17. Kalinauskiene, L., Cekuoliene, D., Van Ijzendoorn, M., Bakermans-Kranenburg, M., Juffer, F. and Kusakovskaja, I. 2009. Supporting insensitive mothers: The Vilnius randomized control trial of video

feedback intervention to promote maternal sensitivity and infant attachment. *Child: Care, Health & Development*. **35**, 613-23.

18. Klein Velderman, M., Bakermans-Kranenburg, M., Juffer, F. and Van Ijzendoorn, M. 2006. Preventing preschool externalizing behaviour problems through video-feedback intervention in infancy. *Infant Mental Health Journal*. **27**(5), 466-93.
19. Stein, A., Woolley, H., Senior, R., Hertzman, L., Lovel, M., Lee, J. et al. 2006. Treating disturbances in the relationship between mothers with bulimic eating disorders and their infants: A randomized controlled trial of video feedback. *The American Journal of Psychiatry.* **163,** 899-906.
20. Van Zeijl, J., Mesman, J., Van Ijzendoorn, M., Bakermans-Kranenburg, M., Juffer, F. and Stolk, M. 2006. Attachment-based intervention for enhancing sensitive discipline in mothers of 1- to 3-year-old children at risk for externalizing behaviour problems: a randomized controlled trial. *Journal of Consulting and Clinical Psychology*. **74**(6), 994-1005.
21. Goodman, R. 1997. The Strengths and Difficulties Questionnaire: A Research Note. *Journal of Child Psychology and Psychiatry.* **38**, 581-586.
22. Ravens-Sieberer, U., Wille, N., Badia, X., Bonsel, G., Burström, K., Cavrini, G., et al. 2010. Feasibility, reliability, and validity of the EQ-5D-Y: results from a multinational study. *Quality of Life Research*, **19**(6), 887-897.
23. National Institute for Health and Clinical Excellence. 2008. *Social Value Judgements: Principles for the development of NICE guidance (2nd edition).* London: NICE.
24. Briggs, A., Sculpher, M. and Claxton, K. 2006. Decision analytic modelling for health economic evaluation. Oxford: Oxford University Press, 2006.
25. Bakermans-Kranenburg, M., Van Ijzendoorn, M. and Juffer, F. 2003. Less is more: meta-analyses of sensitivity and attachment interventions in early childhood. *Psychological Bulletin.* **129**(2), 95-215.
26. Scott, S. 2008. Parenting Programs. In: Rutter, M., Bishop, D., Pine, D., Scott, S., Stevenson, J., Taylor, E. et al., eds. *Rutter's Child and Adolescent Psychiatry*. 5th ed. Oxford: Blackwell Publishing, pp. 1046-61.
27. Olds, D., Sadler, L. and Kitzman, H. 2007. Programs for parents of infants and toddlers: Recent evidence from randomized trials. *Journal of Child Psychology and Psychiatry*. **48**(3-4), 355-91
28. Barlow, J., Schrader McMillan, A., Kirkpatrick, S., Ghate, D., Barnes, J. and Smith, M. 2010. Health-led interventions in the early years to enhance infant and maternal mental health: A review of reviews. *Child and Adolescent Mental Health*. **15**(4), 178-85.
29. Allen, G. 2011. *Early Intervention: The Next Steps*. London: Cabinet Office.
30. Center on the Developing Child at Harvard University. 2007. *A Science-Based Framework for Early Childhood Policy*. Cambridge, Massachusetts: Harvard University.
31. Children and Young People’s Health Outcomes Forum. 2012. *Report of the Children and Young People’s Health Outcomes Forum*. London: Department of Health.
32. Olds, D., Henderson, C., Cole, R., Eckenrode, J., Kitzman, H. and Luckey, D. 1998. Long-term effects of nurse home visitation on children's criminal and antisocial behaviour: 15-year follow-up of a randomized trial. *Journal of the American Medical Association*. **280**, 1238-44.
33. Shaw, D., Dishion, T., Supplee, L., Gardner, F. and Arnds, K. 2006. Randomized Trial of a Family-Centred Approach to the Prevention of Early Conduct Problems: 2-Year Effects of the Family Check-Up in Early Childhood. *Journal of Consulting and Clinical Psychology*. **74**(1), 1-9.
34. Bakermans-Kranenburg, M., Van Ijzendoorn, M. and Juffer, F. 2008. Earlier is better: a meta-analysis of 70 years of intervention improving cognitive development in institutionalized children. Monographs of the Society for Research in Child Development. **73**(3), 279-93.
35. Shonkoff, J., Boyce, W. and McEwan, B. 2009. Neuroscience, molecular biology, and the childhood roots of health disparities: Building a new framework for health promotion and disease prevention. *Journal of the American Medical Association*. **301**(21), 2252-9.
36. Gluckman, P. and Hanson, M. (eds). 2005. *The Fetal Matrix: Evolution, Development and Disease*. Cambridge: Cambridge University Press.
37. Heckman, J. 2006. Skill formation and the economics of investing in disadvantaged children. *Science*. **312**(5782), 1900-2.

38. Tiano, J. and McNeil, C. 2005. The inclusion of fathers in behavioural parent training: a critical evaluation. *Child and Family Behaviour Therapy*. **27**(4), 1-28.
39. Lundahl, B., Tollefson, D., Risser, H. and Lovejoy, M. 2008. A meta-analysis of father involvement in parent training. *Research on Social Work Practice*. **18**(2), 97-106.
40. Department of Health. 2012. *Annual Report of the Chief Medical Officer 2012. Our Children Deserve Better: Prevention Pays*. London: Department of Health.
41. Bowlby, J. 1969. *Attachment and Loss*. New York: Basic Books.
42. Patterson, G. 1982. *Coercive Family Process*. Eugene, OR: Castilia.
43. Scott, S. and Dadds, M. 2009. Practitioner review: When parent training doesn't work: Theory-driven clinical strategies. *Journal of Child Psychology and Psychiatry*. **50**(12), 1441-50.
44. Campbell, M., Fitzpatrick, R., Haines, A., Kinmonth, A.L., Sandercock, P., Spiegelhalter, D., et al. 2000. Framework for the design and evaluation of complex interventions to improve health. *British Medical Journal*. **321**, 694-6.
45. Achenbach, T. and Edelbrock, C. 1983. *Manual for the child behaviour checklist*. Burlington, VT: Queen City.
46. Spanier, G. 1976. Measuring dyadic adjustment: New scales for assessing the quality of marriage and similar dyads. *Journal of Marriage and Family*. **38**, 15-28.
47. Beck, A.T. 1978. *The depression inventory*. Philadelphia: Center for Cognitive Therapy.
48. Byford, S., Barratt, B., Roberts, C., Wilkinson, P., Dubika, B., Kelvin, R. G. et al. 2007. Cost-effectiveness of selective serotonin reuptake inhibitors and routine specialist care with and without cognitive-behavioural therapy in adolescents with major depression. *British Journal of Psychiatry.* **191**, 521-527.
49. Harrington, R., Peters, S., Green, J., Byford, S., Woods, J. and McGowan, R. 2000. Randomised comparison of the effectiveness and costs of community and hospital based mental health services for children with behavioural disorders. *British Medical Journal*. **321**(7268), 1047-1050.
50. Byford, S., Cary, M., Barrett, B., Aldred, C., Charman, T., Howlin, P. et al. 2013. Cost-effectiveness of a communication-focused therapy for pre-school children with autism. *Journal of Autism and Developmental Disorders*. (Submitted).
51. Bronte-Tinkew, J., Carrano, J., Horowitz, A., & Kinukawa, A. (2008). Involvement among resident fathers and links to infant cognitive outcomes. *Journal of Family Issues*, *29*(9), 1211-1244.
52. Goldsmith, H. H., Reilly, J., Lemery, K. S., Longley, S., & Prescott, A. (1993). *Preschool Laboratory Temperament Assessment Battery (PS Lab-TAB; Version 1.0).* Technical Report, Department of Psychology, University of Wisconsin-Madison.
53. Carlson, S. M., & Zelazo, P. D. (2014). Minnesota executive function scale: test manual. *Reflection Sciences, LLC. St. Paul, MN: LLC.*
54. Department of Health. 2011. *NHS Reference Costs*. London: Department of Health.
55. Curtis, L. 2012. *The unit costs of health and social care 2012*. University of Kent at Canterbury: Personal Social Services Research Unit.
56. Drummond, M., Sculpher, M., Torrance, G.W., O'Brien, B. and Stoddart, G.L. 2005. *Methods for the economic evaluation of health care programmes*. Oxford: Oxford University Press.
57. Van Hout, B.A., Al, M.J. , Gordon, G.S. and Rutten, F.F.H. 1994. Costs, effects and c/e-ratios alongside a clinical trial. *Health Economics.* **3**, 309–19.
58. Fenwick, E. and Byford, S. 2005. A guide to cost-effectiveness acceptability curves. *British Journal of Psychiatry*. **187**, 106-108.
59. Stinnett, A.A. and Mullahy, J. 1998. Net health benefits: a new framework for the analysis of uncertainty in cost-effectiveness analysis. *Medical Decision Making*. 1998, **18**(2Suppl), 65-80.
60. Sainsbury Centre for Mental Health. 2009. *The chance of a lifetime: Preventing early conduct problems and reducing crime*. London: Sainsbury Centre for Mental Health.
61. Scott, S., Knapp, M., Henderson, J. and Maughan, B. 2001. Financial cost of social exclusion: follow up study of antisocial children into adulthood. *British Medical Journal*. **323**, 191-4.
62. Bonin, E.-M., Stevens, M., Beecham, J., Byford, S. and Parsonage, M. 2011. Costs and longer-term savings of parenting programmes for the prevention of persistent conduct disorder: a modelling study. *BMC Public Health*. **11**, 803.

63. Phillips Z, Ginnelly L, Sculpher M, et al (2004). Review of guidelines for good practice in decision-analytic modelling in health technology assessment. Health Technology Assessment, 2004, **8**(36).
64. Barton, P., Bryan, S. and Robinson, S. 2004. Modelling in the economic evaluation of health care: selecting the appropriate approach. *Journal of Health Services Research and Policy*. **9**(2), 110-8.
65. Scott, S. 2007. Conduct disorders in children. *British Medical Journal*. **334**(7595), 646.
66. Moffitt, T. and Scott, S. 2008. Conduct Disorders of Childhood and Adolescence. In: Rutter, M., Bishop, D., Pine, D., Scott, S., Stevenson, J., Taylor, E. et al., eds. *Rutter's Child and Adolescent Psychiatry*. 5th ed. Oxford: Blackwell Publishing, pp. 543-564.
67. Angold, A. and Costello, E. 2001. The Epidemiology of Disorders of Conduct: Nosological Issues and Comorbidity. In: Hill, J. and Maughan, B., eds. *Conduct Disorders in Childhood and Adolescence*. Cambridge: Cambridge University Press.
68. Petitclerc, A. and Tremblay, R. 2009. Childhood disruptive behaviour disorders: review of their origin, development, and prevention. *La Revue Canadienne de Psychiatrie*. **54**(4), 222-31.
69. Caspi, A., Harrington, H., Moffitt, T., Begg, D., Dickson, N., Langley, J. et al. 1997. Personality differences predict health-risk behaviours in young adulthood: Evidence from a longitudinal study. *Journal of Personality and Social Psychology*. **73**(5), 1052-63.
70. Shaw, D., Gilliom, M., Ingoldsby, E. and Nagin, D. 2003. Trajectories leading to school-age conduct problems. *Developmental Psychology*. **39**(2), 189-200.
71. Moffitt, T. 2003. Life-course-persistent and adolescence-limited antisocial behaviour: A 10-year research review and a research agenda. Causes of conduct disorder and juvenile delinquency. In: Lahey, B., Moffitt, T. and Caspi, A., eds. *Causes of conduct disorder and juvenile delinquency*. New York: Guildford Press. pp. 49-75.
72. Scott, S., O'Connor, T. and Futh, A. 2006. What makes parenting programmes work in disadvantaged areas? London: Institute of Psychiatry.
73. Miner, J. and Clarke-Stewart, K. 2008. Trajectories of externalizing behaviour from age 2 to age 9: Relations with gender, temperament, ethnicity, parenting and rater. *Developmental Psychology*. **44**(3), 771-86.
74. Campbell, S. 1995. Behaviour Problems in Preschool Children: A review of recent research. *Journal of Child Psychology and Psychiatry*. **36**(1), 113-49.
75. Petitclerc, A., Boivin, M., Dionne, G., Zoccolillo, M. and Tremblay, R. 2009. Disregard for rules: The early development and predictors of a specific dimension of disruptive behaviour disorders. *Journal of Child Psychology and Psychiatry*. **50**(12), 1477-84.
76. Shaw, D. and Gross, H. 2008. Early childhood and the development of delinquency: What we have learned from recent longitudinal research. In: A Lieberman , eds. *The long view of crime: A synthesis of longitudinal research*. New York: Springer, pp. 79-127.
77. Baydar, N., Reid, M. and Webster-Stratton, C. 2003. The role of mental health factors and program engagement in the effectiveness of a preventive parenting program for head start mothers. *Child Development*. **74**(5), 1433-53.
78. National Institute for Health and Care Excellence. 2013. *Antisocial behaviour and conduct disorders in children and young people: recognition, intervention and management.* London: NICE.
79. Arnold, D.S., O'Leary, S.G., Wolff, L.S. and Acker, M.M. 1993. The Parenting Scale: A measure of dysfunctional parenting in discipline situations. *Psychological Assessment.* **5**, 137-144.

eDocument 2. Pre-specified statistical analysis plan

Additional details for statistical policies

Continuous/numeric variables that follow an approximately normal distribution are summarised using the mean and standard deviations. Skewed variables are summarised using the median and inter-quartile range. Categorical variables are presented as frequencies and percentages. Scores from psychometric scales are treated as numeric. Histograms and box-plots are used to assess distributional assumptions and to check for possible outliers. The significance level for statistical tests is set at 5%. The primary analysis was conducted on an intention to treat basis. The safety population comprises children and their parents/ caregivers who had received at least one intervention session as part of the VIPP-SD programme plus those allocated to the usual care group who did not receive any VIPP intervention. All analyses (unless indicated otherwise) were undertaken using Stata version 13 and 15 following a pre-specifed statistical analysis plan.

Statistical Analysis Plan (SAP) version 2.0
for Healthy Start, Happy Start
Based on protocol version 6.0

Principal Investigator: Paul Ramchandani

Study Investigators (alphabetically):
Marian Bakermans-Kranenburg
Sarah Byford
Marinus van IJzendoorn
Stephen Scott
Alan Stein
Jane Warwick
Hilary Watt

Study Management Team: Daphne Babalis and Christine O'Farrelly

SAP Working Group: Hilary Watt and Jane Warwick (Sarah Byford and Poushali Ganguli for the economics section)

Version	Date	Lead Author
2.0	7 May 2019	Hilary Watt

1 Approvals: approved by:

Name	Signature	Role	Date
Paul Ramchandani		Chief Investigator	
Paul Stallard		Chair, Trial Steering committee	
Hilary Watt		Trial Statistician	
Jane Warwick		Senior Trial Statistician	
Sarah Byford		Senior Trial Economist	
Poushali Ganguli		Trial Economist	

Changes from version 1 of the Statistical Analysis Plan

There is a difference in the distribution of follow-up times at the "5 month" assessment of primary and secondary endpoints. Therefore we are changing the methods of analysis to take account of this.

For the primary analysis, a number of sensitivity analyses already looked at adjustment for length of follow-up (from date of randomisation to date of follow-up assessment). Therefore the series of analyses and sensitivity analyses for this primary end-point (PPACS) will be unchanged (at both 5 month and at 24 month follow-up assessments). However, to increase the credibility of our results, we shall now denote the analysis which adjusts for length of follow-up as our primary analysis (at both 5 month and 24 month follow-up assessments).

For secondary analyses, we did not previously state that we would adjust for follow-up time. We will now report analyses adjusted for follow-up time, as our primary method of analysis for all secondary outcomes. For SDQ and CBCL (total scores assessed by primary and secondary care-givers), we will report these without adjustment for age, as sensitivity analyses.

For other secondary outcomes, such as subscales of PPACS, of SDQ and of CBCL, and all parental outcomes, we shall now report analyses adjusted for length of follow-up. This will apply at both 5 month outcome assessments and 24 month outcome assessments.

All other adjustment variables will remain unchanged.

A new CACE analysis is added which refers to fidelity as well as to number of sessions received.

Changes from official signed Statistical Analysis Plan

Sample size: the statement of sample size calculation (**section 3.6 Sample Size**) was changed to clarify that this was based on an independent samples t-test.

Change from Per-protocol analysis to Complier average causal effects analysis.

Section 4.3: Complier average causal effects (CACE) population on population used in the analysis, now refers to complier average population, whereas previously a per-protocol population was described here. Similarly, **Section 6.3: Complier average causal effects analysis,** the statistical analysis section, has been updated to reflect this new analysis. The title and intended contents of **Table 0** is changed to refer to this complier average causal effects analysis.

Addition of results on Secondary Caregivers into results Tables.

8.7 Table: Primary and Secondary Outcome Measures of Child Behaviour, ITT analysis. and 0

Table: Causal Average Complier Effects analysis on Primary and Secondary Outcomes of Child Behaviour**.** For both of these tables, results on secondary care-givers have been added to the tables, and surplus columns (which were never intentionally included) on sample size were removed (with one column giving sample size retained).

Change to Section 6.1 General Methodology analysis of PPACS primary outcome

The paragraph on "statistical modelling to be used on obtaining estimates of child behaviour outcomes" was moved to improve the overall flow. This was moved from toward the end of this section to become the second paragraph of this section,

Missing outcomes of care-giver outcome will now generally be dealt with by single imputation with a random residual. Section 6.2 now reports that the primary efficacy analysis will be based on multiple imputation including all families, even those with no follow-up data. The text previously read "Any child who has a PPACS at follow-up with at least 80% of the questions completed will be include in this multiple imputation PPACS outcome analysis (and if a number have fewer than 80% complete, we may revise this threshold downwards)." Because there is indeed some missing data at baseline, because not all questions were relevant to younger children, the 80% threshold has been revised downwards to 50%. The simpler strategy for dealing with missing data was clarified; this may be used if there is little missing data or if multiple imputation fails.

Removal of technical aside from section 6.2 Primary Efficacy Analysis since it is not relevant to analysis as presented here.

Change to Section 6.2: Primary Efficacy Analysis: further sensitivity analyses have been added.

The sensitivity analyses based on questions within the PPACS measure are an addition. A further sensitivity analysis has been added to allow for differences in duration of follow-up between groups. A further sensitivity analysis will omit from the analysis anyone with no PPACS outcome (or when it is less than 80% complete). **Section 10.2: Forest Plot for Sensitivity Analysis** has been updated to refer to these additional sensitivity analyses.

An inter-rater reliability analysis of PPACS has been added as **Section 6.6: Inter-rater Reliability Analysis.**

Measurement of alcohol consumption by AUDIT questionnaire will no-longer be included in tables of outcomes.

Section 6.4: Secondary Efficacy Analysis. The description of secondary efficacy analyses no-longer refer to the measurement of alcohol consumption by AUDIT questionnaire. This will no-longer be included in **8.12 Table: Secondary Outcomes on Parental/ Care-giver Well-being and Parental Practice by Gender of Care-giver, ITT analysis.** AUDIT is no-longer being measured at 24 months (as stated in protocol version 6).

Change to 8.14 Table: Number of Therapists delivering ViPP Intervention

This table was added to the initial SAP.

There was a minor change to the title 9.4 9.4 Table: Primary Outcome, PPACS, at 5 months and at 24 months follow-up: Regression coefficients, ITT analysis. to reflect the inclusion of 24 month as well as 5 month outcome measures, which was already included in the body of the table.

2 Study Objectives and Hypotheses to be tested

2.1 Primary Objective

To undertake a randomised controlled trial to evaluate whether, compared to treatment as usual in the NHS, a brief parenting intervention (Video Feedback to Promote Positive Parenting and Sensitive Discipline) leads to lower levels of behavioural problems in young children who are at high risk of developing these problems.

2.2 Secondary Objective

To undertake an economic evaluation to assess the cost-effectiveness of the intervention compared to treatment as usual.

2.3 Primary Hypothesis

Among children at high risk of behavioural problems aged twelve to thirty-six months, adding a brief video-feedback parenting intervention (ViPP-SD) to treatment as usual will reduce enduring behavioural problems measured at five months post-randomisation, using the PPACS interview.

2.4 Secondary Hypotheses

5) Among children with high levels of behavioural problems aged twelve to thirty-six months, adding a brief video-feedback parenting intervention (ViPP-SD) to treatment as usual will reduce enduring behavioural problems measured at two years post-randomisation, using the PPACS interview.

6) Among children with high levels of behavioural problems aged twelve to thirty-six months, adding a brief video-feedback parenting intervention (ViPP-SD) to treatment as usual will reduce enduring behavioural problems measured at 5 months and two years post-randomisation, using the Child Behaviour CheckList (CBCL), completed by primary care-giver, and using the SDQ, which will be completed by primary care-giver and also by a nursery practitioner /teacher.

7) Among children with high levels of behavioural problems aged 12–36 months, adding a brief video-feedback parenting intervention (VIPP-SD) to treatment as usual will lead to improved parent-child interactions (improved parental sensitivity and engagement) measured at 5 months.

8) Among children with high levels of behavioural problems aged twelve to thirty-six months, adding a brief video-feedback parenting intervention (ViPP-SD) to treatment as usual will provide a cost effective use of resources, at two years post-randomisation.

3 Background and Introduction

3.1 Introduction

Behavioural problems affect 5-10% of children, and children with established behavioural problems have significantly worse outcomes through childhood and into adult life. They have an increased risk of psychiatric disorders, antisocial behaviour and criminality, drug and alcohol misuse, educational failure and physical ill health. As well as these high levels of difficulties and unhappiness for young people and their families, there are also large costs incurred by society through the health, social care and criminal justice systems.

A key risk factor for the development of behavioural problems is the quality of the parental care that children receive: low levels of sensitive parenting and greater use of harsh discipline have been causally linked to the development of behavioural problems. Interventions which work with parents and care-givers to improve their parenting have been found to reduce child behavioural problems, and intervening early in children's lives has the potential to be particularly effective in improving outcomes, as well as having beneficial effects for parental health and wellbeing.

Most research to date has focussed on older children, when behavioural problems are more established, and thus more difficult to treat. Interventions have also focussed predominantly on mothers, with very few interventions involving fathers or a second caregiver, despite accumulating evidence that interventions involving two parents or caregivers can be more effective than those engaging just one. The proposed intervention (VIPP-SD) has a developing evidence base as an early preventive intervention and has the potential to be delivered widely across the NHS as part of an early intervention programme. Young children and their care-givers have regular contact with the NHS, yet evidence is needed to ensure that resources are directed in the most effective manner. The trial has been designed to provide this evidence, as the first large randomised controlled trial to test whether an early video feedback intervention (ViPP-SD) is an effective and cost-effective approach to reducing behavioural problems in at-risk young children. It addresses an area of key concern to the NHS and represents an opportunity to reduce the burden of behavioural problems on individuals, families and society. If shown to be effective, the intervention could be delivered widely across the NHS to parents and care-givers of young children at risk of behavioural problems as part of community based services.

The case for early preventive intervention is becoming increasingly established. The 2012 Chief Medical Officer's report, 'Our Children Deserve Better: Prevention Pays' clearly highlights the social and economic benefits of early, preventive interventions in child health. The report focusses particularly on the need for interventions to improve the early parent-child relationship, as a way of reducing the risk of psychiatric disorder in children. However, it is essential that proposed early interventions are shown to be effective and cost-effective. This is the first RCT to test VIPP-SD in a UK setting. VIPP-SD has the potential to be an effective and cost-effective early intervention for behavioural problems.

3.2 Study Design

The study is a two-arm, parallel group, researcher-blind, randomised controlled trial (RCT), to test the clinical and cost effectiveness of a video-feedback intervention (ViPP-SD) for parents of young children (12-36 months) at risk of behavioural difficulties. The trial will involve 300 families, who will be randomly allocated into one of two groups.

3.3 Treatment Groups

- The intervention group, who will receive the video-feedback intervention (described in **2.5** below) (n=150).
- Treatment as usual (control group), where usual care is also described below in **2.5** (n=150).

3.4 Study Population

Children aged 12-36 months with behavioural problems and their parents/care-givers. Parents/ care-givers need to be aged ≥ 18 years and to provide written informed consent. Where possible, both parents/ care-givers will be involved, but if not (because of lack of availability or lack of consent), the intervention may involve the one available parent/ care-giver. To be eligible to enter the study, a child needs to score in the top 20% for behavioural problems on the Strengths and Difficulties Questionnaire (SDQ), based on population norms.

Children will be excluded for the following reasons:

- Child has severe sensory impairment or learning disability
- Parent/ care-giver has insufficient English language to complete questionnaire assessments
- Siblings participating in the trial
- Families participating in active family court proceedings
- Parent/care-giver is participating in another closely related research trial.

Any changes to the above inclusion and exclusion criteria will be reflected in a revised protocol, but will not necessarily be updated here. The analysis will reflect the patients recruited in practice, according to definitions of randomised populations given in section 4.

3.5 Intervention being tested

ViPP-SD is a home-based intervention, delivered over six sessions at approximately fortnightly intervals, which shows high levels of parental acceptability. Each session involves filming parent-child interactions and giving parents feedback based on these video clips.

The intervention will be delivered by trained, supervised health professionals, predominantly health visitors. They will deliver the intervention in research participants' homes (or another location according to participant preference). The key role of the therapists will be to develop a trusting relationship with the participants in the treatment arm, and to deliver the treatment in 6 sessions in accordance with the manual. They will be supervised, and the treatment will be monitored closely for fidelity to the manual by the clinical supervisor (and a proportion will be taped and assessed by an independent researcher trained in the intervention).

- Four core sessions: these aim to enhance the parent's capacity to identify the child's exploratory behaviour and attachment cues and to respond to them appropriately
- Two booster sessions: these are spaced one month apart, and the key messages are repeated using continuing video interaction material at each session

Therapists responsible for delivering the intervention will be trained by the developers of ViPP-SD and will undertake supervised clinical practice before becoming a therapist on the trial.

Participants in both groups will continue to receive their usual care. Usual care may include a range of services such as the following:

- health visitor services
- GP advice
- early intervention mental health services linked to children's centres
- parenting advice and support sessions

Data on concurrent use of health services will be collected including number of sessions offered, where they were provided, and which healthcare (or other non-healthcare) professionals provided the care.

3.6 Sample Size

The total sample size will be 300 participants.

If losses to follow-up are 20%, this leaves 120 participants per group with follow-up data. We would then have 80% and 90% power to detect standardised effect sizes of 0.36 and 0.42 respectively, at the 5% significance level, using an independent samples t-test. In addition, we have stated that our analysis will adjust for baseline behavioural score, research centre and age of child, and parental involvement (one versus two) which will increase power, probably to over 90% for the 0.36 effect size (since such adjustment will reduce the residual error variance in our model). Kahan and colleagues (2014, ref 1) found that covariate adjustment for 1 to 4 variables in trials increased power from 80% to a median of 93% power in their sample of 12 outcomes assessed across 8 studies.)

We have conservatively allowed for a potential drop-out rate of 20% because of the longer follow-up time in the proposed study, even though previous intervention studies detailed below have maintained retention rates of over 90% at follow-up.

The pooled effect size for all randomised controlled trials to date that have used the same video feedback intervention (ViPP) is 0.46 (Bakermans-Kranenburg, 2013, personal correspondence). Other relevant literature for interventions for behavioural problems, predominantly in slightly older children, yield higher effect size estimates. In the systematic reviews undertaken for the most recent NICE guidance on conduct disorders in children and young people (ref 2), the pooled effects for parent-focussed interventions yielded estimated effect sizes of 0.69 standard deviations for researcher-rated outcome (the main outcome measure in the proposed study), and 0.54 for parent-rated outcome. In a study of the Incredible Years programme in 2-9 year olds (ref 3) effect sizes ranged from 0.48-0.78. In the SPOKES trial (ref 4) of intervention for parents of 6 year olds, the effect size for behavioural outcomes was 0.52 SD difference between the treatment and control group. Where parenting programmes have been rolled out across the UK (ref 5-6) similar effect sizes have been found, albeit in non-randomised designs (effect sizes ranged from 0.44-0.71).

Change from official signed draft: the statement of sample size calculation (**section 3.6 Sample Size**) was changed to clarify that this was based on a t-test.

	**Baseline**	**5 month f/u**	**24 month f/u**

3.7 Schedule of Time and Events

The study funding began on 1 October 2014, with recruitment commencing in June 2015. Recruitment finished in July 2017. All children will be followed up for two years from date of randomisation.

3.8 ViPP-SD Visit Schedule

	**VIPP Intervention Schedule[a]**					
**Visit**	**1**	**2**	**3**	**4**	**5**	**6**
Day/Week/Month	Day 14-28 (+/- 7 days) post randomisation	Visit 1 plus 14 days (+/-7 d)	Visit 2 plus 14 days (+/-7 d)	Visit 3 plus 14 days (+/-7 d)	Visit 4 plus 21 days (+/-7 d)	Visit 5 plus 21 days (+/-7 d)

Note. [a]*Visit schedule is a guide for optimal treatment delivery, variation is expected given the pragmatic context of the trial.*

3.9 Figure 1. Assessment schedule

Visit	1	7	8
Day/Week/Month		Month 5 post randomisation (+/- 3 weeks)	Month 24 post randomisation (+/- 2 weeks)
Informed consent	X		
Inclusion & exclusion criteria	X		
Demographics and medical history	X		
AUDIT-C	X	X	
Randomisation	X		
CBCL on child assessed by - parent/care-giver 1	X	X	X
CBCL on child assessed by - parent/care-giver 2	X	X	X
SDQ on child assessed by - parent/care-giver 1	X	X	X
SDQ on child assessed by - parent/care-giver 2	X	X	X
SDQ on child – assessed by teacher/ care-giver outside the family			X
GAD-7 - parent/care-giver 1	X	X	X
GAD-7 - parent/care-giver 2	X	X	X
PPACS interview on child	X	X	X
PHQ-9 – parent/care-giver 1	X	X	X
PHQ-9 – parent/care-giver 2	X	X	X
Revised Dyadic Adjustment Scale - parent/care-giver 1	X	X	X
Revised Dyadic Adjustment Scale - parent/care-giver 2	X	X	X
Parenting Scale - parent/care-giver 1	X	X	X
Parenting Scale - parent/care-giver 2	X	X	X
CA-SUS	X	X	X
Parent-Child interactions collection of videos	X	X	X
Feedback questionnaire		X	
Serious adverse events	X	X	X

Note that not all families have a participating secondary care-giver, so data on second care-giver will not always be available.

3.10 Randomisation

Randomisation lists were prepared by a statistician independent of the trial (Xinxue Liu) using computer generated 1:1 allocation (ViPP intervention vs treatment as usual) and variable block sizes. Randomisation will be stratified by treatment centre and by number of parents/ care-givers participating (one or two). The randomisation lists (one for each strata) will be uploaded on to InForm (the study electronic data capture system) and eligible subjects allocated online to the next available treatment code in the appropriate randomisation list.

4 Randomised populations

4.1 Intent-to-Treat Population

The primary analysis will be conducted using intention to treat approach. A substantial effort will be made to obtain follow-up data on all participants (unless they drop out of the study and withdraw their consent to our following them up). Baseline data will be analysed on all patients, unless they withdraw consent for this data to be used.

4.2 Safety population

The safety population will be children and their parents/ care-givers who have received at least one intervention as part of the ViPP programme, compared to those allocated to the control group who have not received any ViPP intervention. Adverse events will be compared between arms according to these safety definitions of groups.

4.3 Complier average causal effects (CACE) population

A secondary analysis will be undertaken, using a CACE analysis to determine the effect of receiving the intervention, rather than merely being randomised to receive it. For the purposes of this analysis, we define "receiving the intervention" to mean that the family has attended at least four of the six planned intervention visits, and "not receiving the intervention" as receiving zero to three sessions. These first four sessions relate to delivery of the core intervention, with the extra two sessions being booster sessions. Delays to the planned schedule will not be considered a protocol violation, so long as at least four of the six planned intervention visits were completed before the outcome is assessed. Initially all families in the control group will be analysed as not receiving the intervention (unless, off protocol, they receive at least four sessions of the ViPP intervention).

A sensitivity analysis will be undertaken on this CACE analysis, where families who receive some other video feedback intervention during the study (off protocol) will be analysed as if they received the intervention (unless we know that they received an incomplete course, and that this incomplete course consisted of no more than three sessions).

A further CACE analysis will be performed, to determine the effect of receiving the intervention to a high level of fidelity, rather than merely being randomised to receive it (and rather than receiving it with poor fidelity). To count as receiving the intervention, families would have to receive at least four sessions with a mean level of fidelity above the 25th centile (and then above the 50th centile) of the distribution of fidelity scores within this randomized trial.

Change from official signed draft: previously there was a per protocol population definition described here, rather than a complier average causal effects population.

Change from version 1 to version 2: a new CACE analysis is added which refers to fidelity as well as to number of sessions received.

5 Variables of Analysis

5.1 Primary Efficacy Variable

The severity of behavioural problems will be assessed using the PPACS interview to the primary care-giver and standard PPACS scoring method. This will be collected at 5 months (primary outcome is at this time point) and at 24 months after randomisation.

5.2 Secondary Efficacy Variables

These will be collected at 5 months and at 24 months after randomization.

1) Child Behaviour assessed by the CBCL questionnaire (total score), as assessed by primary and, if possible, secondary care-givers.

2) Strengths and Difficulties Questionnaire (SDQ) relating to qualities of the child, to be completed by primary and, if possible, secondary care-givers.

3) Strengths and Difficulties Questionnaire (SDQ) relating to qualities of the child, as assessed by a nursery teacher/ care-giver from outside the family (only at 2 years follow-up).

4) Parental couple functioning assessed by the Revised Dyadic Adjustment Scale (RDAS), for each parent/ care-giver when two are engaged in the study.

5) Parental mood assessed by the Patient Health Questionnaire 9 (PHQ9), for each parent/ care-giver when both are engaged in the study.

6) Parenting practice assessed by the Parenting Scale, for each parent/ care-giver when both are engaged in the study.

7) Health and social care services provided to the child and services parents/care-givers have attended in relation to their child's needs, using a modified version of the Child and Adolescent Service Use Schedule (CA-SUS).

8) Parental anxiety assessed by the Generalized Anxiety Disorder 7 (GAD7) for each parent/ care-giver when both are engaged in the study.

9) Parental sensitivity and engagement will be assessed, if we are able to secure an additional grant beyond the HTA grant to fund the collection of this data (based on detailed assessment of interviews).

5.3 Safety Variables

There are no obvious adverse events that are expected to be related to the ViPP intervention and the risk to participants is believed to be low. We have therefore not specified any particular type of adverse events in advance, other than serious adverse events, for routine collection. Information on all serious adverse events affecting any family members who are participating in the study (i.e. the child and the one or two participating care-givers) will be collected for the duration of the follow-up period, irrespective of the likely connection to the intervention. Information on mild to moderate adverse events will be collected only if the event might plausibly be attributable to the intervention.

It is important to note that some adverse events (such as incarceration or death of sibling) may be reported indirectly, through the process of re-scheduling appointments, for example, rather than directly as an adverse event. The database notes columns will be scrutinised prior to analysis in order to identify these. Hospitalisation of the child, will be collected via the CASUS form which is designed to collect health care utilisation data for the economic analysis.

5.4 Demographic Variables

For first parent: Relationship to child – parent, adoptive parent, foster parent, step parent, grandparent, sibling, other

For both parents/ care-givers:

Parent age

Parent gender

Parent ethnicity

Relationship status –married/remarried/civil partnership, single (never married, never in civil partnership), cohabiting, divorced (including separated civil partnership, including legally separated), widowed (including from civil partnership)

Highest qualifications (no GSCE's, GCSEs or equivalent, College (A-levels, NVQ or BTEC), undergraduate, postgraduate.

Employment status – full time, part time, paid parental leave, student, no paid job.

Whether or not it is their first child

Number and ages of their other children if not first child.

Child demographics

Age at randomisation

Gender

Ethnicity

Birth weight

Breast feeding attempted or not

Number of months exclusively breast fed

6 Statistical Methodology

6.1 General Methodology

Descriptive data: Continuous/ numeric variables that follow an approximately Normal distribution will be summarised using the mean and standard deviations. Skewed variables will be summarised using the median and inter-quartile range. Categorical variables (binary and ordinal and multinomial) will be presented as frequencies and percentages. Scores from psychometric scales will be treated as numeric.

Statistical models to be used for obtaining estimates of the treatment effect on child behaviour outcomes: These statistical models will have either PPACS score or CBCL score or SDQ at follow-up (based on assessment by the primary or secondary -care giver) as the outcome, and will be adjusted by baseline measurements of the same score, time from randomisation to follow-up assessment, recruitment centre, age of child at recruitment and parental involvement (one or two parents/ care-givers) as fixed effects[1]. SDQ measured by a teacher/ some-one external to the family, will be adjusted for primary care-giver SDQ at baseline.

[1] Adjusting for centre as a random effect, although planned in the protocol, will not be statistically robust owing to there being only a very small number of centres. As an alternative, centre has been included as a fixed effect in the proposed model.

Checking of assumptions underlying hypothesis tests and models: Histograms and box-plots will be used to assess the distributional assumptions required for the models that will be fitted and to check for possible outliers. Log transformations will be applied, where appropriate, in order to render outcomes variable distributions closer to the Normal. If neither the variable itself nor its log result in residuals from the regression analysis that are close enough to the Normal distribution to allow regression results to be reported confidently, then boot-strap techniques will be undertaken to calculate confidence intervals, after relaxing the assumptions of normality. The relationship between the outcomes and other variables will be explored graphically, using scatter plots and box-plots.

Statistical significance level: Results will be interpreted according to a 5% significance level.

Missing data on primary outcome measure (PPACS): Before starting the data analysis, the level, pattern and likely causes of the missingness in the baseline variables and outcomes will be investigated by forming appropriate tables. This information will be used to determine whether the level and type of missing data has the potential to introduce bias into the analysis of the PPACS results.

Missing data on individual questions which make up measurement scales:

For our care-giver outcome variables, we will use the manuals on each questionnaire to determine the appropriate treatment of missing data items. When there is no information on this in the manuals, then we will replace the missing values with single imputed values with a random residual, based on responses to other items on the same subscale of the questionnaire, provided not more than 20% of the questions within the questionnaire

or 33% of the questions within a subscale (for questionnaires other than the PPACS) are missing for that person. Otherwise, we will consider that outcome variable summary score to be missing for that person.

There is no information on treatment of missing values in the manuals for PPACS, our primary outcome measure. For PPACS, CBCL and SDQ (our child behaviour outcomes) a multiple imputation will be used on the individual questions within each questionnaire at baseline and at follow-up, in order to optimise our treatment of missing data. These multiple imputation analyses will be the primary analyses for these outcomes. Any child who has a PPACS at follow-up with at least 50% of the questions completed will be included in this multiple imputation PPACS outcome analysis. We will base our imputation on completed questions and subscale scores from the PPACS questionnaire at outcome and total scores at earlier time points. For multiple imputation at baseline, we will base our imputation on completed questions and subscale scores from within the PPACS questionnaire at baseline only. The methods will be the same for CBCL and for SDQ. These multiple imputation models can be difficult to fit, and sometimes it is not feasible to come up with a solution. If this proves to be the case, after trying a number of different strategies aiming to get a solution, or if there is reason to believe that assumptions are violated to the extent that any solution is not statistically robust, then we will use a simpler method of dealing with missing. The simpler method is to scale up psychometric scores to account for missing responses on individual items that make up the scale. The sum of non-missing items is scaled up, according to the proportion of the maximum total scale score derives from non-missing items. If there are fewer than 10% of the patients who have 10% or more of their questions missing within the specified questionnaires, then the simpler strategy may be used in any case, since it will produce virtually identical results.

Changes from official signed draft: The paragraph on "statistical modelling to be used on obtaining estimates of child behaviour outcomes" was moved to improve the overall flow. This was moved from toward the end of this section to become the second paragraph of this section. Missing outcomes of care-giver outcomes will now generally be dealt with by single imputation with a random residual. For PPACS, the text previously read "Any child who has a PPACS at follow-up with at least 80% of the questions completed will be included in this multiple imputation PPACS outcome analysis (and if a number have fewer than 80% complete, we may revise this threshold downwards)." Because there is indeed some missing data at baseline, because not all questions were relevant to younger children, the 80% threshold has been revised downwards to 50%. The multiple imputation for missing items within PPACS will now be based on individual items and subscale totals, rather than solely on individual items, so that there will generally be some residual variation in the imputation models. The most fundamental change to the PPACS multiple imputation is detailed in section 6.2 primary efficacy analysis below. The simpler strategy for dealing with missing data was clarified; this may be used if there is little missing data or if multiple imputation fails.

Change from SAP version 1.0: We now adjust all key outcome analyses (primary and secondary outcomes on child behaviour and on parental well-being, including their subscales) for time from randomisation to follow-up assessment.

6.2 Primary Efficacy Analysis

The primary endpoint, externalizing behaviour (PPACS), will be analysed at follow-up, using the regression model suggested above, with multiple imputation of individual missing questions within the PPACS questionnaire, as described under treatment of missing values. This approach will maximise the power, by including baseline scores into the regression analysis. It will also ensure the most appropriate and unbiased treatment of anyone who has missing data on any item within the PPACS questionnaire. The primary analysis will also incorporate multiple imputation for anyone whose PPACS at outcome is completely missing (or with fewer items completed than the threshold). At 5 months follow-up, these will be imputed based on randomised group, gender of child, age of child at 5 month assessment, baseline PPACS, CBCL and SDQ scores. At 2 year follow-up, these will be imputed based on randomised group, gender of child, age of child at 2 year assessment, and PPACS, CBCL and SDQ scores (at 5 month follow-up, if available or else using these PPACS, CBCL and SDQ scores at baseline).

Sensitivity analyses will firstly be a complete case analysis (with multiple imputation solely of missing items within PPACS). We will perform further sensitivity analyses by assuming firstly that completely missing PPACS scores are one SDC lower (than anticipated by multiple imputation), and secondly by assuming that they are one SDC higher (than anticipated by multiple imputation) (truncated to maximum and minimum possible scores on PPACS). [SDC represents one standard deviation of changes in PPACS where changes are

calculated as PPACS at f/u minus PPACS at baseline].There will be further sensitivity analysis, based on those with missing items within the PPACS questionnaire. They are filled in initially on a missing at random assumption, and then by assuming that they are one point higher on each item than MI prediction, and then one point lower (truncated to maximum and minimum possible scores on each item).

Additional sensitivity analysis will be added to take account of our original analysis plan, which did not incorporate adjustment for length of follow-up (from randomisation to measurement of outcome) .Lack of adjustment for this length of follow-up will be combined with the different assumptions about missing values, including on the complier average causal effects model and final model listed below. The complier average causal effects model also has a sensitivity analysis noted on it. This sensitivity (without adjustment for length of follow-up) will be applied to total scores on PPACS, SDQ (primary and secondary care-giver assessment) and CBCL (primary and secondary care-giver assessment).

An additional sensitivity analysis on the PPACS will only include PPACS measurements when they have at least 80% of questions completed. Anyone without PPACS measurements at follow-up (on this basis) will be excluded from the analysis (since this is the analysis method that was written into our official signed draft).

Changes and additions from official signed draft: The primary efficacy analyses now include multiple imputation of outcomes, for people whose PPACS is completely missing at follow-up. The original analysis is now listed as a sensitivity analysis. Other sensitivity analyses have been added. The technical aside was removed from this section since it is not relevant to analysis as presented here.

Changes from SAP 1: Sensitivity analyses and primary analyses have been reversed, in respect of adjustment for length of follow-up. This adjustment is now incorporated into the main analyses (of both 5 month and of 24 month outcomes). Lack of such adjustment is now incorporated into sensitivity analyses.

6.3 Complier average causal effects analysis

This will be performed by two stage least squares regression analysis on **PPACS**. The definitions for compliers and non-compliers/treatment not received is given in 4.3. As is standard practice for CACE analysis, it is assumed that there is no effect of being randomized to a particular group, other than the effect that results from receiving the intervention in practice (with at least 4 visits).

This section replaces the per protocol analysis in the official signed draft.

6.4 Secondary Efficacy Analysis

The impact of the intervention on the secondary measures assessing child behaviour and parental outcomes, will be assessed using the same approach as for the primary endpoint, with the same treatment of missing values for CBCL and SDQ, and a different treatment of missing values that does not involve multiple imputation for all other outcomes, as detailed above. Secondary outcomes which are measured on both parents/ care-givers: namely child behaviour (SDQ and CBCL), (parental couple functioning (assessed using RDAS), parental mood (assessed using PHQ9), parenting practice (assessed using the Parenting Scale), and parental anxiety (assessed using GAD7)) will be analysed separately by gender. In the case where both the primary and secondary care-givers are included in this study and both are of the same gender, the mean score of the two will be calculated and used in the analysis in order to avoid violating assumptions of independence. Parental sensitivity may be added here and analysed in the same way (subject to separate funding being received for its measurement).

Changes from official signed draft: AUDIT assessment of alcohol consumption is no-longer being analysed as a secondary outcome.

Change from SAP version 1.0: The outcomes included in the secondary outcomes have been clarified. This paragraph now corresponds to the analysis methods written into the footnotes of the tables (i.e. the tables of this current SAP and also in the previous SAP version 1.0). The analysis methods for SDQ and CBCL was not mentioned in the text of the previous SAP, so the tables was the only source for information that clarified their method of analysis in SAP version 1.0.

6.5 Safety Analysis

A table of serious adverse events and any adverse events considered to be relevant will be produced, by type of event and by treatment received.

The table will show numbers of children who have at least one such adverse event of a given type and then numbers of families where at least one parent/care-giver is affected by this type of adverse event. Percentages (of children/ families) will be calculated and presented. Proportions will be compared between treatment groups using Fisher's exact test.

6.6 Inter-rater Reliability Analysis of Primary Outcome, PPACS

The inter-rater reliability of PPACS is assessed, using an intra-class correlation coefficient. This will be based on separate ratings of PPACS scores by at least two different raters, on 30 patients (based on recorded interviews) and at each of the three time points. Inter-rater reliability will be reported separately at each time point. These reliability coefficients will be reported in the text.

Change from SAP version 1.0: We now have subsets of 30, rather than 45, patients at each time point.

6.7 Interim Analysis

There are no planned interim analyses.

7 Economic Analysis

The economic evaluation will take the NHS/Personal Social Services perspective preferred by NICE (ref 7), and will include the use of all health and social services by the child and by parent/care-givers in relation to their child's needs over the 24-month follow-up.

7.1 Economic measures

Data on resource use will be recorded in interview with parents/care-givers using a modified version of the Child and Adolescent Service Use Schedule (CA-SUS) and will be collected at baseline (covering the previous 3 months), and at the 5- and 24-month follow-ups (covering the period since previous interview). Modifications to the CA-SUS were based on review of recent literature and clinical feedback.

7.2 Valuation and analysis of resources

For each item of service use reported in the CA-SUS, a unit cost will be applied and the total costs for each participant calculated. Unit costs will be for the most recent financial year over which the trial data will be collected, and will be reported in UK pounds sterling. Costs and outcomes in the second year will be discounted by 3.5%, as recommended by NICE (ref 71).

Intervention costs will be calculated using a standard micro-costing (bottom-up) approach (ref 8) and will be based on therapist salaries plus on-costs (employers' national insurance and superannuation contributions) and appropriate capital, administrative and managerial overheads. Data on intervention contacts will be collected directly from health visitor records and indirect time (preparation, supervision, administration, travel etc.) will be estimated using questionnaires completed by each health visitor delivering the intervention on the time they spend on different activities. National UK unit costs will be applied to all hospital contacts (ref 9), community health and social care contacts (ref 10), medication (ref 11), and accommodation services (ref 10).

Resource use items will not be tested for statistical significance to avoid excessive significance testing and because the focus of the economic analysis is on cost and cost-effectiveness.

7.3 Costs and short-term cost-effectiveness

The total costs, as well as costs per sector, in each trial arm will be summarised using the mean and standard deviation. Despite the skewed nature of cost data, differences in mean costs will be analysed using standard parametric t-tests to enable inferences to be made about the arithmetic mean (ref 12).

Cost-effectiveness will be explored in terms of the primary outcome measure (PPACS). This will be based on multiple imputations of individual missing items within the PPACS questionnaire, rather than for people when PPACS is completely missing at follow-up (in line with the primary outcome analysis). It will be assessed using (i) through the calculation of incremental cost-effectiveness ratios (ICER the additional cost of one intervention compared with another divided by the additional effect) (ref 13) and (ii) using the net monetary benefit (NMB) approach based on the use of a linear function of costs and effect and assuming specified values for the willingness to pay for each additional unit of effect (ref 14). Uncertainty around the cost and effectiveness estimates will be represented by cost-effectiveness acceptability curves (CEAC) (ref 15). The CEAC is a plot of the probability of the intervention being cost-effective (y axis) for a range of possible values of willingness to pay per unit improvement in outcome (x axis).

Additionally, cost-consequences analysis will outline the costs alongside all secondary outcome measures (listed in Section 5.2) in order to explore potential economic impacts of the intervention more broadly. All analyses of cost will be adjusted for recruitment centre, age of child at recruitment, and parental involvement (one or two parents/ care-givers), in line with clinical analyses, plus the baseline variable of interest (cost, PPACS).

Long-term cost-effectiveness

Longer term outcomes will be explored using decision analytic modelling, utilising data from the trial supplemented by available data from literature. Whilst no method of direct estimation of health-related quality of life, and thus quality adjusted life years (QALYs) currently exists for infants and pre-school children, if data allow, attempts will be made to model expected cost-utility by using existing data sets to map the results of the SDQ onto an appropriate utility scale. It may be possible to map alternative outcomes onto utility scores at the time of data analysis, so this will be finalised during the course of the study.

8 Index of Tables

Tables that will be presented in the report.

8.1 Table: Route of Screening and Recruitment of Families into the RCT

Recruitment Route from data collected at initial SDQ assessment:	Children screened for behavioural problems<br>**n (%)**	Eligible Families<br>**n (%)**	Family recruited into RCT<br>**n (%)**<br>**[% of eligibles]**	Two parents/ care-givers participating in RCT<br>**n (%)**<br>**[% of recruits]**
children centre	n (%)	n (%)	n (%)<br>[% of eligibles]	n (%)<br>[% of recruits]
community association	n (%)	n (%)	n (%)<br>[% of eligibles]	n (%)<br>[% of recruits]
health centre	n (%)	n (%)	n (%)<br>[% of eligibles]	n (%)<br>[% of recruits]
Mail	n (%)	n (%)	n (%)<br>[% of eligibles]	n (%)<br>[% of recruits]
Nursery	n (%)	n (%)	n (%)<br>[% of eligibles]	n (%)<br>[% of recruits]
Online	n (%)	n (%)	n (%)<br>[% of eligibles]	n (%)<br>[% of recruits]
unknown	n (%)	n (%)	n (%)<br>[% of eligibles]	n (%)<br>[% of recruits]
**Total**	**n (100%)**	**n (100%)**	**n (100%)**<br>**[% of eligibles]**	**n (100%)**<br>**[% of recruits]**

8.2 Table: Centre of Families Screened and Recruited into the RCT

Recruitment Centre:	Children screened for behavioural problems<br>n (%)	Eligible Families<br>n (%)	Family recruited into RCT<br>n (%)<br>[% of eligibles]	Two parents/ care-givers participating in RCT<br>n (%)<br>[% of recruits]
Camden	n (%)	n (%)	n (%)<br>[% of eligibles]	n (%)<br>[% of recruits]
Islington	n (%)	n (%)	n (%)<br>[% of eligibles]	n (%)<br>[% of recruits]
Hillingdon	n (%)	n (%)	n (%)<br>[% of eligibles]	n (%)<br>[% of recruits]
Oxfordshire	n (%)	n (%)	n (%)<br>[% of eligibles]	n (%)<br>[% of recruits]
(other)	n (%)	n (%)	n (%)<br>[% of eligibles]	n (%)<br>[% of recruits]
Unknown	n (%)	n (%)		
**Total**	**n (100%)**	**n (100%)**	**n (100%)<br>[% of eligibles]**	**n (100%)<br>[% of recruits]**

8.3 Table: Baseline Characteristics of Families Screened and Recruited into the RCT

	Children screened for behavioural problems	Family satisfies RCT Recruitment Criteria	Family recruited into RCT	Two parents/ care-givers participating in RCT
	n=	n=	n=	n=
Male Child:				
Age of Child: Mean (SD) [range]				
**Demographics of primary care-giver:**				
Relationship to child:				
Biological or adoptive or step parent				
Foster parent				
Other				
Male:				
Age: Mean (SD) [range]				
Ethnicity: White				
Mixed				
Asian				
Black				
other				
Highest Qualification: GCSE or lower				
A-level/ NQV/ BTEC				
Graduate				

8.4 Table: Baseline Demographics of Children by Randomised Group: N (%)

**Randomised Group:**	**ViPP Group**			**Control Group**		
**Number of Participating Parents/ Care-givers:**	**Two**	**One**	**1 or 2**	**Two**	**One**	**1 or 2**
	n=	n=	n=	n=	n=	n=
Gender: male						
Age of Child: mean (SD) [months]						
Birth-weight of child: Mean (SD) [kg]						
Duration of exclusive breast feeding: Mean (SD) [months]						
**Ethnicity:** White						
Mixed						
Asian						
Black						
Other						
**Recruitment Route for Screening Questionnaire (%):** Health Visitor developmental review or other clinic						
Online advertisement (e.g. netmums, NCT, etc.)						
Poster/face to face recruitment at childrens centre						
Poster/face to face recruitment/mailshot at another centre (GPs, CAMHS)						
By Post						
Word of Mouth						
Other						
**Recruitment Centre (%):**						
Camden						
Islington						
Hillingdon						
Oxfordshire						
(other regions)						

8.5 Table: Baseline Demographics of Primary Care-giver by Randomised Group: N (%)

**Randomised Group:**	**ViPP Group**			**Control Group**		
**Number of Participating Parents/ Care-givers:**	**Two**	**One**	**1 or 2**	**Two**	**One**	**1 or 2**
	n=	n=	n=	n=	n=	n=
Gender: male						
Age: Mean (SD) [range] [years]						
Parental status of primary care-giver:						
Parent (including step or adoptive)						
Foster parent						
Other						
Marital status of primary care-giver:						
Married/ civil partnership/ cohabiting						
Divorced/ widowed/ legally separated						
Single and none of the above						
In relationship, but not cohabiting						
Participating child is first child: Yes						
Ethnicity: White						
Mixed						
Asian						
Black						
Other						
Employment status: Employed						
Paid parental leave						
Self-employed						
Student						
Looking after home and children						
Highest Qualification: GCSE or lower						
A-level/ NQV/ BTEC						
Graduate						
Mood (PHQ9): Mean (SD)						
Anxiety (GAD7): mean (SD)						
Couple Functioning (RDAS): mean (SD)						
Hazardous alcohol consumption (%)						

Footnote: Hazardous alcohol consumption is based on AUDIT questionnaire using a cut-off of 5

8.6 Table: Baseline Demographics of Secondary Care-giver by Randomised Group: N (%)

Whether or not this care-giver is participating	Participating		Not participating	
**Randomised group:**	**VIPP group**	**Control Group**	**VIPP group**	**Control Group**
	**n=**	**n=**	**n=**	**n=**
Gender: Male:				
Age: Mean (SD) [range]				
Parental status of primary care-giver:				
Parent (including step or adoptive)				
Foster parent				
Other				
Marital status of primary care-giver:				
Married/ civil partnership/ cohabiting				
Divorced/ widowed/ legally separated				
In relationship but not cohabiting				
Single and none of the above				
Participating child is first child: Yes				
One other child				
Two or more other children				
Ethnicity: White				
Mixed				
Asian				
Black				
Other				
Employment status: Paid work, employed				
Self-employed				
Paid parental leave				
Student				
Retired				
Looking after home and children				
Highest Qualification: GCSE or lower				
A-level/ NQV/ BTEC				
Graduate				
Mood (PHQ9): Mean (SD)				
Anxiety (GAD7): mean (SD)				
Couple Functioning (RDAS): mean (SD)				
Hazardous alcohol consumption				

Footnote: Hazardous alcohol consumption is based on AUDIT questionnaire using a cut-off of 5

8.7 Table: Primary and Secondary Outcome Measures of Child Behaviour, ITT analysis.

Randomised Group:	ViPP Group		Control Group		Treatment Effect (95% CI)	p-value
	n	Mean (SD)	n	Mean (SD)		
**PPACS (primary care-giver reported):**						
Baseline						
5 month follow-up						
24 month follow-up						
**Child behaviour (CBCL) (primary care-giver reported)**						
Baseline						
5 month follow-up						
24 month follow-up						
**Strengths and Difficulties of Child (SDQ) (primary care-giver reported)**						
Baseline						
5 month follow-up						
24 month follow-up						
24 months follow-up . (reporter external to family)						
**Child behaviour (CBCL) (secondary care-giver reported)**						
Baseline						
5 month follow-up						
24 month follow-up						
**Strengths and Difficulties of Child (SDQ) (secondary care-giver reported)**						
Baseline						
5 month follow-up						
24 month follow-up						

n=number of children with outcome reported by specified care-giver

Treatment effect is the difference in mean between treatment groups from regression analysis of the outcome measure on the baseline score of that same measurement, on treatment centre, on randomised group, on length of follow-up, on age of child and on number of parents/ care-givers participating (all treated as fixed effects).

Change from official signed draft: Results on secondary care-givers have been added to the above table. Surplus columns on sample size (which were never intentionally included) have been removed.

8.8 Table: Causal Average Complier Effects analysis on Primary and Secondary Outcomes of Child Behaviour

Randomised Group:	ViPP Group		Control Group		Treatment Effect (95% CI)	p-value
	n	Mean (SD)	n	Mean (SD)		
**PPACS (primary care-giver reported):**						
Baseline						
5 month follow-up						
24 month follow-up						
**Child behaviour (CBCL) (primary care-giver reported)**						
Baseline						
5 month follow-up						
24 month follow-up						
**Strengths and Difficulties of Child (SDQ) (primary care-giver reported)**						
Baseline						
5 month follow-up						
24 month follow-up						
24 month follow-up (reporter . external to family)						
**Child behaviour (CBCL) (secondary care-giver reported)**						
Baseline						
5 month follow-up						
24 month follow-up						
**Strengths and Difficulties of Child (SDQ) (secondary care-giver reported)**						
Baseline						
5 month follow-up						
24 month follow-up						

n=number of children with outcome reported by specified care-giver

Treatment effect is the difference in mean between treatment groups from regression analysis of the outcome measure on the baseline score of that same measurement, on treatment centre, on randomised group, on length of follow-up, on age of child and on number of parents/ care-givers participating (all treated as fixed effects).

Change from official signed draft: The title is changed, so that this now reflect the complier average causal effects model. Results on secondary care-givers have been added to the above table. Surplus columns on sample size (which were never intentionally included) have been removed.

8.9 Table: Subscales of Primary PPACS and Secondary Child Behaviour (CBCL) Outcomes Measures of Child Behaviour, primary care-giver reported, ITT analysis

**Randomised Group:**	**ViPP Group**		**Control Group**		**Treatment Effect**	
	**n**	**Mean (SD)**	**n**	**Mean (SD)**	**(95% CI)**	**p-value**
**PPACS - ADHD/ Hyperkinesis**						
Baseline						
5 month follow-up						
24 month follow-up						
**PPACS - conduct problems**						
Baseline						
5 month follow-up						
24 month follow-up						
**CBCL- externalising**						
Baseline						
5 month follow-up						
24 month follow-up						
**CBCL- internalising**						
Baseline						
5 month follow-up						
24 month follow-up						
**CBCL - attention**						
Baseline						
5 month follow-up						
24 month follow-up						
**CBCL - aggression**						
Baseline						
5 month follow-up						
24 month follow-up						

Treatment effect is the difference in mean between treatment groups from regression analysis of the outcome measure on the baseline score of that same measurement, on treatment centre, on randomised group, on length of follow-up, on age of child and on number of parents/ care-givers participating (all treated as fixed effects).

8.10 Table: Subscales of Strengths and Difficulties Questions (SDQ) of Child Behaviour, ITT analysis, reported by primary care-giver except where specified otherwise

Randomised Group:	ViPP Group		Control Group		Treatment Effect (95% CI)	
	n	Mean (SD)	n	Mean (SD)		p-value
**SDQ - Externalising**						
Baseline						
5 month follow-up						
24 month follow-up						
24 month follow-up, reporter external to the family						
**SDQ - Conduct**						
Baseline						
5 month follow-up						
24 month follow-up						
24 month follow-up, reporter external to the family						
**SDQ - Hyperactivity**						
Baseline						
5 month follow-up						
24 month follow-up						
24 month follow-up, reporter external to the family						
**SDQ - Emotional**						
Baseline						
5 month follow-up						
24 month follow-up						
24 month follow-up, reporter external to the family						
**SDQ - Peer Problems**						
Baseline						
5 month follow-up						
24 month follow-up						
24 month follow-up, reporter external to the family						
**SDQ - Impact Score**						
Baseline						
5 month follow-up						
24 month follow-up						
24 month follow-up, reporter external to the family						
**SDQ - Pro-Social**						
Baseline						
5 month follow-up						
24 month follow-up						
24 month follow-up, reporter external to the family						

Treatment effect is the difference in mean between treatment groups from regression analysis of the outcome measure on the baseline score of that same measurement, on treatment centre, on randomised group, on length of follow-up, on age of child and on number of parents/ care-givers participating (all treated as fixed effects).

8.11 Table: Secondary Outcomes on Child Behaviour according to perception of male and female care-givers, ITT analysis

**Randomised Group:**	**ViPP Group**		**Control Group**		**Treatment Effect (95% CI)**	**p-value**
	**n**	**Mean (SD)**	**n**	**Mean (SD)**		
**Child behaviour (CBCL): female care-giver reported:**						
Baseline						
5 month follow-up						
24 month follow-up						
**male care-giver reported:**						
Baseline						
5 month follow-up						
24 month follow-up						
**Strengths and Difficulties of Child (SDQ): female care-giver reported**						
Baseline						
5 month follow-up						
24 month follow-up						
**male care-giver reported**						
Baseline						
5 month follow-up						
24 month follow-up						
**CBCL externalising: female care-giver reported:**						
Baseline						
5 month follow-up						
24 month follow-up						
**male care-giver reported:**						
Baseline						
5 month follow-up						
24 month follow-up						
**CBCL internalising: female care-giver reported:**						
Baseline						
5 month follow-up						
24 month follow-up						
**male care-giver reported:**						
Baseline						
5 month follow-up						
24 month follow-up						
**SDQ Externalising: female care-giver reported**						
Baseline						
5 month follow-up						
24 month follow-up						

**male care-giver reported**
Baseline
5 month follow-up
24 month follow-up

Treatment effect is the difference in mean between treatment groups from regression analysis of the outcome measure on the baseline score of that same measurement, on treatment centre, on randomised group, on length of follow-up, on age of child and on number of parents/ care-givers participating (all treated as fixed effects).

Where any child has two male or two female care-givers, their scores will be averaged prior to inclusion in the above analysis, to avoid violating the assumption of independence.

8.12 Table: Secondary Outcomes on Parental/ Care-giver Well-being and Parental Practice by Gender of Care-giver, ITT analysis.

Randomised Group:	ViPP Group		Control Group		Treatment Effect (95% CI)	p-value
	n	Mean (SD)	n	Mean (SD)		
**Mothers/ female care-givers**						
**Parenting Practice (parenting scale)**						
Baseline						
5 month follow-up						
24 month follow-up						
**Parental/care-giver Mood (PHQ9)**						
Baseline						
5 month follow-up						
24 month follow-up						
**Parental/care-giver Anxiety (GAD7)**						
Baseline						
5 month follow-up						
24 month follow-up						
**Parental/ care-giver Couple Functioning (RDAS)**						
Baseline						
5 month follow-up						
24 month follow-up						
**Parental/ care-giver Sensitivity***						
Baseline						
5 month follow-up						
24 month follow-up						
**Fathers/ male care-givers**						
**Parenting Practice (parenting scale)**						
Baseline						
5 month follow-up						
24 month follow-up						
**Parental/care-giver Mood (PHQ9)**						
Baseline						
5 month follow-up						
24 month follow-up						

**Parental/care-giver Anxiety (GAD7)**
Baseline
5 month follow-up
24 month follow-up
**Parental/ care-giver Couple Functioning (RDAS)**
Baseline
5 month follow-up
24 month follow-up
**Parental/ care-giver Sensitivity***
Baseline
5 month follow-up
24 month follow-up

Treatment effect is the difference in mean between treatment groups from regression analysis of the outcome measure on the baseline score of that same measurement, on treatment centre, on randomised group, on length of follow-up, on age of child and on number of parents/ care-givers participating (all treated as fixed effects).

Where any child has two male or two female care-givers, their scores will be averaged prior to inclusion in the above analysis, to avoid violating the assumption of independence.

Changes from official signed draft: AUDIT assessment of alcohol consumption has been removed from this table.

8.13 Table: Attendance at Sessions and Satisfaction with Treatment amongst those allocated to the ViPP group

		One parent/ care-giver agreed to participate	Two parents/ care-givers agreed to participate	
			First	Second
		n (%)	n (%)	n (%)
Attended all sessions				
Number of session attended				
	5			
	4			
	3			
	2			
	1			
	0			
Satisfaction, questionnaire scores, amongst those who attended at least one session: n: mean (SD)				
Overall score				
Helpfulness				
Enjoyableness				
Relevance				
Format				
Amongst those who attend all sessions				
Amongst those who attend 4-5 sessions				
Amongst those who attend 1-3 sessions				

8.14 Table: Number of Therapists delivering ViPP Intervention

Centre	Number of Patients who Received ViPP	Number of Therapists who delivered ViPP	Number of patients treated by each therapist: Median [Range]
Camden			
Hillingdon			
Islington			
Oxfordshire			
Barking and Dagenham			
Peterborough			
Hertfordshire			
TOTAL			

Changes from the official signed draft: This table has been added.

8.15 Table: Treatment Fidelity, Therapist reported

	Visit 1	Visit 2	Visit 3	Visit 4	Visit 5	Visit 6
**Primary Care-giver:**	n=	n=	n=	n=	n=	n=
**Present at visit (%):**						
**Elements of intervention completed (%):**						
Speak for child						
Attachment messages						
Set Message						
Set Explanation						
Exploration of messages						
Written messages						
**Secondary Care-giver:**	n=	n=	n=	n=	n=	n=
Present at visit **(%):**						
**Elements of intervention completed (%):**						
Speak for child						
Attachment messages						
Set Message						
Set Explanation						
Exploration of messages						
Duration of visit, therapist reported: Mean (SD)						

8.16 Table: Treatment Fidelity, Independent Assessment based on Audio Recordings

	Visit 1	Visit 2	Visit 3	Visit 4	TOTAL
**Primary Care Giver present at visit: Video recordings obtained: n/N (%)**					
**Proportion of therapists who recorded at least one visit and included here: n/N (%)**					
**Number of visits assessed**					
**Elements of intervention completed (%):**					
Speak for child					
Attachment messages					
Set Message					
Set Explanation					
Exploration of messages					
Written messages					
**Secondary Care-Giver present a visit: Video recordings obtained: n/N (%)**					
**Elements of intervention completed (%):**					
Speak for child					
Attachment messages					
Set Message					
Set Explanation					
Exploration of messages					
Duration of video recording: Mean (SD)					
Duration of visit, therapist reported: Mean (SD)					

8.17 Table: Adverse Events

	Received ViPP (at least one appointment)	Allocated to control group (and did not receive any appointments for ViPP)	
	(n=number of families)	(n=number of families)	p-value by Fisher's exact test for difference between groups
**For the child: n (% of families)**			
Any Adverse Events that are considered relevant, by category, giving number of children affected			
Serious Adverse Events, by category, giving number of children affected			
**For parents/care-givers: n (% of families)**			
Any Adverse Events that are considered relevant, by category, giving number of families affected			
Serious Adverse Events, by category, giving number of families affected			

9 Index of Supplementary Tables

9.1 Table: Sensitivity Analyses for lack of adjustment for length of follow-up: Primary and Secondary Outcomes on Child Behaviour, ITT analysis

Randomised group	ViPP Group		Control Group		Treatment Effect	
	n	Mean (SD)	n	Mean (SD)	(95% CI)	p-value
**PPACS (primary care-giver reported):**						
Baseline						
5 month follow-up						
24 month follow-up						
**Child behaviour (CBCL) (primary care-giver reported)**						
Baseline						
5 month follow-up						
24 month follow-up						
**Strengths and Difficulties of Child (SDQ) (primary care-giver reported)**						
Baseline						
5 month follow-up						
24 month follow-up						
24 month follow-up (reporter external to family)						
**Child behaviour (CBCL) (secondary care-giver reported)**						

Baseline
5 month follow-up
24 month follow-up
**Strengths and Difficulties of Child (SDQ) (secondary care-giver reported)**
Baseline
5 month follow-up
24 month follow-up

Treatment effect is the difference in mean between treatment groups from regression analysis of the outcome measure on the baseline score of that same measurement, on treatment centre, on randomised group, on age of child and on number of parents/ care-givers participating (all treated as fixed effects).

n=number of children with outcome reported by specified care-giver

Change from official signed draft: This table has been added, reflecting new sensitivity analyses on key secondary, as well as on PPACS, as noted in section 6.2.

9.2 Table: Demographics of Families with missing PPACS data: N (%)

	**No missing PPACS data**	**PPACS missing at 5 months**	**PPACS missing at 2 years**
	n=	n=	n=
**Two participating parents/ care-givers:**			
Male Child (%):			
Age of Child: mean (SD) [range]			
**Recruitment Route for Screening Questionnaire (%):**			
Health Visitor developmental review or other clinic			
Online advertisement (e.g. netmums, NCT, etc.)			
Poster/face to face recruitment at children's centre			
Poster/face to face recruitment/mailshot at another centre (GPs, CAMHS)			
By Post			
Word of Mouth			
Other			
**Region of screening/ recruitment (%):**			
Camden			
Islington			
Hillingdon			
(other regions)			
**Demographics of Primary Care-giver**			
Gender:			
Age: Mean (SD) [range]			
**Demographics of Secondary Care-giver**			
Gender:			
Age: Mean (SD) [range]			

9.3 Table: Baseline and 5 month values of outcome measures in those with missing PPACS data at follow-up: Mean (SD)

	**No missing PPACS data**	**PPACS missing at 5 months**	**PPACS missing at 2 years**
	n=	n=	n=
**Child behaviour (CBCL)**			
Baseline			
5 month follow-up			
**Strengths and Difficulties of Child (SDQ)**			
Baseline			
5 month follow-up			
**SUB-SCALES OF CBCL**			
**Child Behaviour (CBCL), aggression sub-scale**			
Baseline			
5 month follow-up			
**Child Behaviour (CBCL), attention sub-scale**			
Baseline			
5 month follow-up			
**Mothers/ female care-givers**			
**Parenting Practice (parenting scale)**			
Baseline			
5 month follow-up			
**Parental/care-giver Mood (PHQ9)**			
Baseline			
5 month follow-up			
**Parental/care-giver Anxiety (GAD7)**			
Baseline			
5 month follow-up			
**Parental/ care-giver Couple Functioning (RDAS)**			
Baseline			
5 month follow-up			
**Fathers/ male care-givers**			
**Parenting Practice (parenting scale)**			
Baseline			
5 month follow-up			
**Parental/care-giver Mood (PHQ9)**			
Baseline			
5 month follow-up			
**Parental/care-giver Anxiety (GAD7)**			
Baseline			
5 month follow-up			
**Parental/ care-giver Couple Functioning (RDAS)**			
Baseline			

5 month follow-up

1978

9.4 Table: Primary Outcome, PPACS, at 5 months and at 24 months follow-up: Regression coefficients, ITT analysis.

	5 months follow-up		24 months follow-up	
	Regression coefficient (95% CI)	p-value	Regression coefficient (95% CI)	p-value
Baseline PPACS score				
Number of parents/ care-giver participating (2 versus 1)				
Age of child at randomisation (per month):				
Recruitment centre: Camden				
Islington				
Hillingdon				
(others)				
Constant				

Regression analysis of PPACS scores (and other scores) on the baseline score, treatment centre, randomised group, age of child and number of parents/ care-givers participating (as fixed effects).

10 Figures to present:

10.1 CONSORT Flow Diagram

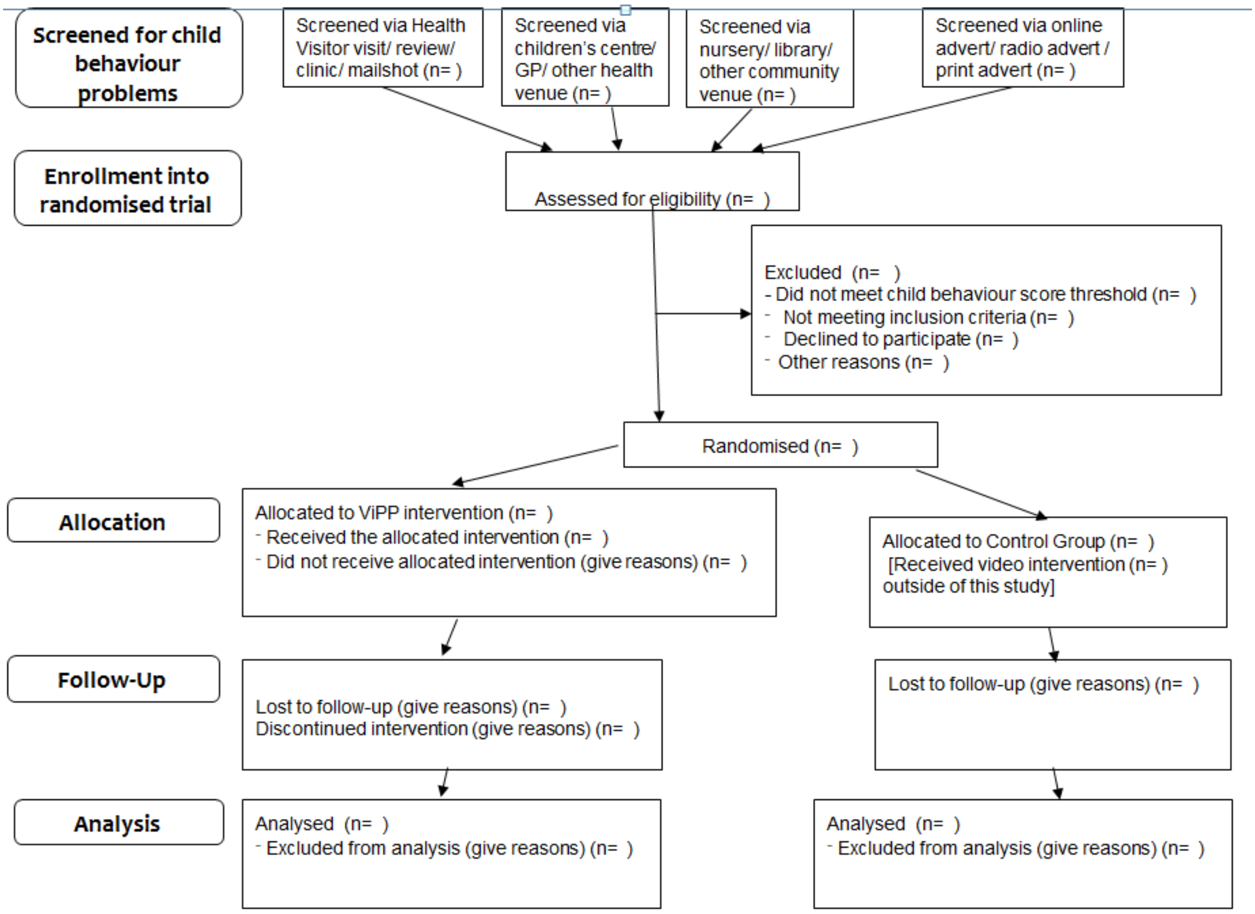

10.2 Forest Plot for Sensitivity Analysis

A forest plot will be plotted for PPACS primary outcome, firstly for our primary analysis, and then for specified sensitivity analyses. For all analyses, missing items from within the PPACS questionnaire will be accounted for by using a multiple imputation, as specified for the primary outcome analysis. Missing PPACS outcome data is defined here as when PPACS is completely missing or sufficient items from within it are missing such that multiple imputation is deemed inappropriate. These will be interpreted to determine whether or not it is possible and/or plausible that missing data has biased the outcome findings. Similar sensitivity analyses may be undertaken for SDQ and CBCL outcome total scores, this time averaging scores over primary and secondary care-givers, prior to analysis, for those families where both are available. All PPACS sensitivity analyses will be presented via such a Forest plot.

All sensitivity analyses on PPACS listed in section 6.2 will be presented in these sensitivity forest plots.

Change from official signed version of analysis plan: This section now refers to the newly added sensitivity analyses in section 6.2 on PPACS outcome and to the amended primary analysis.

Change from SAP version 1: There is no-longer reference to average scores over primary and secondary care-givers, since this does not feature in our main analyses. Hence it does not make sense to report these averages in our sensitivity analyses.

10.3 Forest Plot for Sub-group Analyses

Forest plots will be reported by child age at baseline (12-23 months compared to 24-36 months), and by number of parents participating (1 versus 2). The overall result will be included on the same plot. Sub-group findings will be interpreted cautiously, with emphasis on the overall findings, being appropriate for all families eligible for participation in this RCT.

Fig 3 is an example of such a plot and illustrates the effect of tamoxifen on mammographic density in the IBIS-1 chemoprevention study for various sub-groups, defined by breast cancer risk factors. For the HS, HS study, instead of mean change in density we would have adjusted mean difference in PPACS score between the treatment groups and instead of risk factors (body mass index, age at entry, etc) we would have our proposed sub-groups (far fewer than shown below) and sensitivity analyses.

Fig. 3. Mean change in breast density (95% confidence intervals) with tamoxifen (measured from baseline to 54 months) in subgroups of other risk factors (n = 641 women with baseline breast density of >10%). Negative values reflect decreased breast density; positive values reflect increased breast density. PD = proliferative breast disease; AH = atypical hyperplasia; HRT = hormone replacement therapy. All represents overall unstratified reduction in % density with tamoxifen.

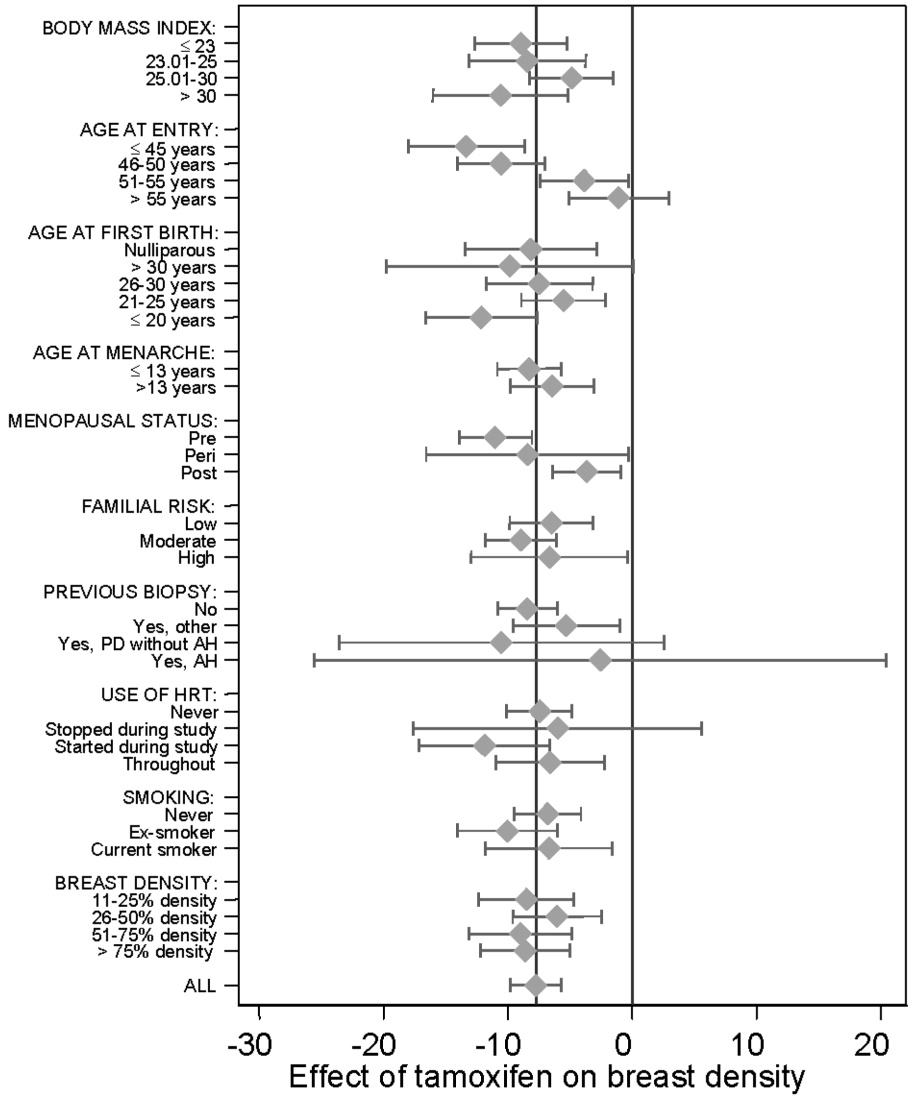
BODY MASS INDEX:
≤ 23
23.01-25
25.01-30
> 30
AGE AT ENTRY:
≤ 45 years
46-50 years
51-55 years
> 55 years
AGE AT FIRST BIRTH:
Nulliparous
> 30 years
26-30 years
21-25 years
≤ 20 years
AGE AT MENARCHE:
≤ 13 years
>13 years
MENOPAUSAL STATUS:
Pre
Peri
Post
FAMILIAL RISK:
Low
Moderate
High
PREVIOUS BIOPSY:
No
Yes, other
Yes, PD without AH
Yes, AH
USE OF HRT:
Never
Stopped during study
Started during study
Throughout
SMOKING:
Never
Ex-smoker
Current smoker
BREAST DENSITY:
11-25% density
26-50% density
51-75% density
> 75% density
ALL
-30
-20
-10
0
10
20
Effect of tamoxifen on breast density

11 References

1) Kahan BC, Jairath V, Dore CJ and Morris TP. 2014. The risks and rewards of covariate adjustment in randomized trials: an assessment of 12 outcomes from 8 studies. Trials **15,** 15-139.
2) National Institute for Health and Care Excellence. 2013. Conduct Disorders in Children and Young People. London: NICE.
3) Gardner, F., Burton, J., Klimes, I. 2006. Randomised controlled trial of a parenting intervention in the voluntary sector for reducing child conduct problems: Outcomes and mechanisms of change. *Journal of Child Psychology and Psychiatry*. **47,** 1123–1132.
4) Scott, S., Sylva, K., Doolan, M., Price, J., Jacobs, B., Crook, S. and Landau, S. 2010. Randomised controlled trial of parent groups for child antisocial behaviour targeting multiple risk factors: the SPOKES project. *Journal of Child Psychology and Psychiatry*. **15**, 48-57.
5) Lindsay, G., Strand, S. and Davis, H. 2011. A comparison of the effectiveness of three parenting programmes in improving parenting skills, parent mental-well-being and children's behaviour when implemented on a large scale in community settings in 18 English local authorities: the parenting early intervention pathfinder (PEIP). *BMC Public Health*. **11**, 962.
6) Lindsay, G. and Strand, S. 2013. Evaluation of the national roll-out of parenting programmes across England: the parenting early intervention programme (PEIP). *BMC Public Health*. **13**(1), 972.
7) National Insitute for Health and Clinical Excellence. Guide to the methods of technology appraisal. London: NICE; 2013.
8) Thompson SG, Barber JA. How should cost data in pragmatic randomised controlled trials be analysed? British Medical Journal. 2000;320:1197-2000.
9) (DH) Department of Health. NHS Reference Costs 2015–16. London DH; 2016.
10) Curtis L, Burns A. Unit Costs of Health & Social Care 2015. Cantebury: PSSRU, 2016.
11) Royal Pharmaceutical Society of Great Britain. British National Formulary London: British Medical Association; 2017.
12) Thompson SG, Barber JA. How should cost data in pragmatic randomised controlled trials be analysed? British Medical Journal. 2000;320:1197-2000.
13) Van Hout BA, Al MJ, Gordon GS, Rutten FH. Costs, effects and cost-effectiveness ratios alongside a clinical trial. Health Economics. 1994;3:309-19.
14) Stinnett AA, Mullahy J: Net health benefits: a new framework for the analysis of uncertainty in cost-effectiveness analysis. Med Decis Making 1998, 18(2S):S65–S80.
15) Barber JA, Thompson SG. Analysis of cost data in randomised trials: an application of the non-parametric bootstrap. Statistics in Medicine. 2000;19:3219-36.
16) Fenwick E, O'Brien B, Briggs A. Cost-effectiveness acceptability curves - facts, fallacies and frequently asked questions. Health Economics. 2004;13:405-15.
