# Supplementary material for: A Brief Home-Based Parenting Intervention to Reduce Behavior Problems in Young Children: A Pragmatic Randomized Clinical Trial
Source: JAMA Pediatr. 2021 Mar 15;175(6):1–10. doi: 10.1001/jamapediatrics.2020.6834 (PMC7961467; doi:10.1001/jamapediatrics.2020.6834)
Supplement: Supplement 2. — eTable 1. Baseline Characteristics of Participating Secondary Caregivers by Treatment Allocation eTable 2. ITT Analysis of Change in Children’s Behavior Problems on the Secondary Outcome Subscales (Primary Caregiver Reported CBCL and SDQ) at 5-Month Follow-up eTable 3. Complier Average Causal Effects Analysis on Primary and Secondary Outcomes of Child Behavior eTable 4. Occurrence of Protocol Deviations and Violations eFigure 1. Forest Plot for Sensitivity Analysis at 5-Month Follow-up eFigure 2. Subgroup Analysis of the Primary Outcome (PPACS Total) by Age of Child, Number of Participating Caregivers, and Baseline (SDQ-Externalizing) Behavior [file jamapediatr-e206834-s002.pdf]

Supplementary Online Content

O'Farrelly C, Watt H, Babalis D, et al. A brief home-based parenting intervention to reduce behavior problems in young children: a pragmatic randomized clinical trial. *JAMA Pediatr.* Published online March 15, 2021.
doi:10.1001/jamapediatrics.2020.6834

eTable 1. Baseline Characteristics of Participating Secondary Caregivers by Treatment Allocation
eTable 2. ITT Analysis of Change in Children's Behavior Problems on the Secondary Outcome Subscales (Primary Caregiver Reported CBCL and SDQ) at 5-Month Follow-up
eTable 3. Complier Average Causal Effects Analysis on Primary and Secondary Outcomes of Child Behavior
eTable 4. Occurrence of Protocol Deviations and Violations
eFigure 1. Forest Plot for Sensitivity Analysis at 5-Month Follow-up
eFigure 2. Subgroup Analysis of the Primary Outcome (PPACS Total) by Age of Child, Number of Participating Caregivers, and Baseline (SDQ-Externalizing) Behavior

This supplementary material has been provided by the authors to give readers additional information about their work.

© 2021 O'Farrelly C et al. *JAMA Pediatrics*.

eTable 1. Baseline Characteristics of Participating Secondary Caregivers by Treatment Allocation

	Trial arm	
**Characteristic**	**VIPP-SD group (n=26)**	**UC group (n=25)**
Sex (male), n (%)	23 (88)	20 (80)
Age (years), mean (SD)	35·4 (6·4)	38·3 (8·9)
Parental status of caregiver, n (%)		
Parent (including step or adoptive)	26 (100)	23 (92)
Grandparent	0 (0)	2 (8)
Ethnicity, n (%)		
White	19 (73)	17 (68)
Mixed	2 (8)	1 (4)
Asian	3 (12)	2 (8)
Black	1 (4)	2 (8)
Other	1 (4)	3 (12)
Relationship status, n (%)		
Married/civil partnership/cohabiting	25 (96)	24 (96)
Divorced/widowed/legally separated	0 (0)	1 (4)
Single and none of the above	0 (0)	0 (0)
In relationship but not cohabiting	1 (4)	0 (0)
Employment status, n (%)		
Employed	26 (100)	16 (64)
Paid parental leave	0 (0)	0 (0)
Self-employed	0 (0)	5 (20)
Student	0 (0)	0 (0)
Looking after home and children	0 (0)	4 (16)
Highest Qualification, n (%)		
GCSE or lower	2 (8)	3 (12)
A-level/ NVQ/ BTEC	4 (15)	5 (20)
Graduate	20 (77)	17 (68)

Data are n (%) or mean (SD). VIPP-SD group=Video-feedback Intervention to promote Positive Parenting and Sensitive Discipline plus usual care.

© 2021 O'Farrelly C et al. *JAMA Pediatrics*.

eTable 2. ITT Analysis of Change in Children's Behavior Problems on the Secondary Outcome Subscales (Primary Caregiver Reported CBCL and SDQ) at 5-Month Follow-up

	**Trial arm**						
	**VIPP-SD group**		**UC group**			**Standardised**	
**Outcome**	**n**	**Mean (SD)**	**n**	**Mean (SD)**	**Adjusted Mean difference[a] (95% CI)**	**Effect size (95% CI)**	**p value**
**CBCL externalising subscale**							
Baseline	151	16·5 (8·1)	149	17·5 (8·3)			
5 months	140	13·3 (8·3)	145	15·2 (8·5)	1·15 (-0·32, 2·63)	0·14 (-0·04, 0·31)	0.12
**CBCL internalising subscale**							
Baseline	151	8·9 (7·2)	149	9·7 (7·5)			
5 months	140	7·1 (6·5)	145	8·5 (6·8)	1·03 (-0·10, 2·16)	0·15 (-0·01, 0·32)	0.07
**CBCL attention subscale**							
Baseline	151	3·8 (2·0)	149	4·2 (2·3)			
5 months	140	2·9 (2·1)	145	3·5 (2·2)	0·25 (-0·18, 0·68)	0·11 (-0·08, 0·30)	0.26
**CBCL aggression subscale**							
Baseline	151	12·7 (6·9)	149	13·3 (7·0)			
5 months	140	10·4 (6·8)	145	11·8 (6·9)	0·98 (-0·25, 2·22)	0·14 (-0·04, 0·32)	0.12
**SDQ externalising subscale**							
Baseline	150	9·4 (3·0)	149	9·4 (3·5)			
5 months	140	7·6 (3·4)	145	8·2 (3·7)	0·68 (0·00, 1·37)	0·18 (0·00, 0·37)	0.05
**SDQ conduct subscale**							
Baseline	150	3·6 (2·0)	149	3·4 (2·1)			
5 months	140	3·1 (1·9)	145	3·1 (2·0)	0·27 (-0·14, 0·67)	0·13 (-0·07, 0·33)	0.20
**SDQ hyperactivity subscale**							
Baseline	150	5·9 (1·9)	149	6·1 (2·2)			
5 months	140	4·5 (2·1)	145	5·1 (2·3)	0·37 (-0·08, 0·81)	0·16 (-0·03, 0·35)	0.11
**SDQ emotional problems subscale**							
Baseline	150	1·4 (1·5)	149	1·6 (1·5)			
5 months	140	1·4 (1·7)	145	1·4 (1·5)	0·01 (-0·32, 0·33)	0·01 (-0·21, 0·22)	0.96
**SDQ peer problems subscale**							
Baseline	150	3·0 (1·9)	149	3·0 (1·8)			
5 months	140	2·3 (1·9)	145	2·5 (2·0)	0·23 (-0·19, 0·64)	0·12 (-0·10, 0·33)	0.29
**SDQ prosocial subscale**							
Baseline	150	5·7 (2·3)	149	5·4 (2·2)			
5 months	140	6·6 (2·0)	145	6·3 (2·2)	-0·07 (-0·47, 0·34)	-0·03 (-0·22, 0·16)	0.75

Data are mean (SD), adjusted treatment difference (95% CI), or standardised effect sizes (95% CI). Lower scores indicate fewer behaviour problems with the exception of the SDQ prosocial subscale where higher scores indicate lower levels of problems.
[a] Mean difference between treatment groups from linear regression analysis adjusted for baseline score, treatment centre, length of follow-up, age of child and number of parents/caregivers participating. Positive differences represent greater adjusted decreases in symptoms in the VIPP-SD rather than in the UC group, with the exception of the SDQ prosocial subscale.
[b] Standardised effect size is the standardised difference in mean (adjusted as above). This is Cohen's d, the difference in mean divided by the standard deviation of controls at follow-up. Cohen's d values of 0.4 to 0.6 represent typical values of Cohen's d. These do not directly relate to clinical importance of results, since an assessment of a characteristics of each individual scale/ subscale is required.
CBCL=Child Behavior Checklist. SDQ=Strengths and Difficulties Questionnaire. VIPP-SD group=Video-feedback Intervention to

© 2021 O'Farrelly C et al. *JAMA Pediatrics*.

promote Positive Parenting and Sensitive Discipline programme plus usual care.

© 2021 O’Farrelly C et al. *JAMA Pediatrics*.

eTable 3. Complier Average Causal Effects[a] Analysis on Primary and Secondary Outcomes of Child Behavior

	Trial arm						
	VIPP-SD group		UC group			Standardised	
**Outcome**	**n**	**Mean (SD)**	**n**	**Mean (SD)**	**Mean difference[b] (95% CI)**	**Effect size[c] (95% CI)**	**p value**
**PPACS**							
Baseline	151	33.5 (9.0)	149	32.4 (10.6)			
5 months	140	28.8 (9.2)	146	30.3 (9.9)	2.59 (0.24, 4.94)	0.26 (0.02, 0.5)	0.03
**CBCL**							
Baseline	151	40.7 (21.7)	149	42.7 (21.1)			
5 months	140	32.5 (20.6)	145	37.2 (21.0)	3.56 (0.04, 7.09)	0.17 (0.00, 0.34)	0.05
**SDQ**							
Baseline	150	13.8 (4.8)	149	14.0 (4.7)			
5 months	140	11.3 (5.1)	145	12.2 (5.2)	1.03 (-0.01, 2.06)	0.20 (0.00, 0.39)	0.05

Data are mean (SD), adjusted treatment difference (95% CI), or standardised effect sizes (95% CI). Lower scores indicate fewer behaviour problems.

[a] Complier average causal effects (CACE) results are based on the assumption that there is no effect of being randomised to VIPP-SD, nor of receiving 1, 2 or 3 VIPP-SD visits. Receiving 4, 5 or 6 VIPP-SD visits counts as receiving the intervention.

[b] Difference in mean is the difference between treatment groups from linear regression analysis of the outcome measure on the baseline score of that same measurement, on treatment centre, on randomised group, on length of follow-up, on age of child and on number of parents/caregivers participating (all treated as fixed effects). Positive differences represent greater adjusted decreases in symptoms in the VIPP-SD rather than in the UC group.

[c] Standardised effect size is the standardised difference in mean (adjusted as above). This is Cohen's d, the difference in mean divided by the standard deviation of controls at follow-up. Cohen's d values of 0.4 to 0.6 represent typical values of Cohen's d. These do not directly relate to clinical importance of results, since an assessment of a characteristics of each individual scale/ subscale is required.

PPACS=Preschool Parental Account of Children's Symptoms. CBCL=Child Behavior Checklist. SDQ=Strengths and Difficulties Questionnaire. VIPP-SD group=Video-feedback Intervention to promote Positive Parenting and Sensitive Discipline programme plus usual care.

© 2021 O'Farrelly C et al. *JAMA Pediatrics*.

eTable 4. Occurrence of Protocol Deviations and Violations

Deviation/violation type	VIPP-SD group	UC group
**Protocol deviations**		
Participant had some missing data	1	2
Participant did not complete follow-up assessment	11	3
Enrolment error	1	2
**Protocol violations**		
Participant did not take up any VIPP-SD treatment	12	N/A

Data are n.

© 2021 O'Farrelly C et al. *JAMA Pediatrics*.

eFigure 1. Forest Plot for Sensitivity Analysis at 5-Month Follow-up

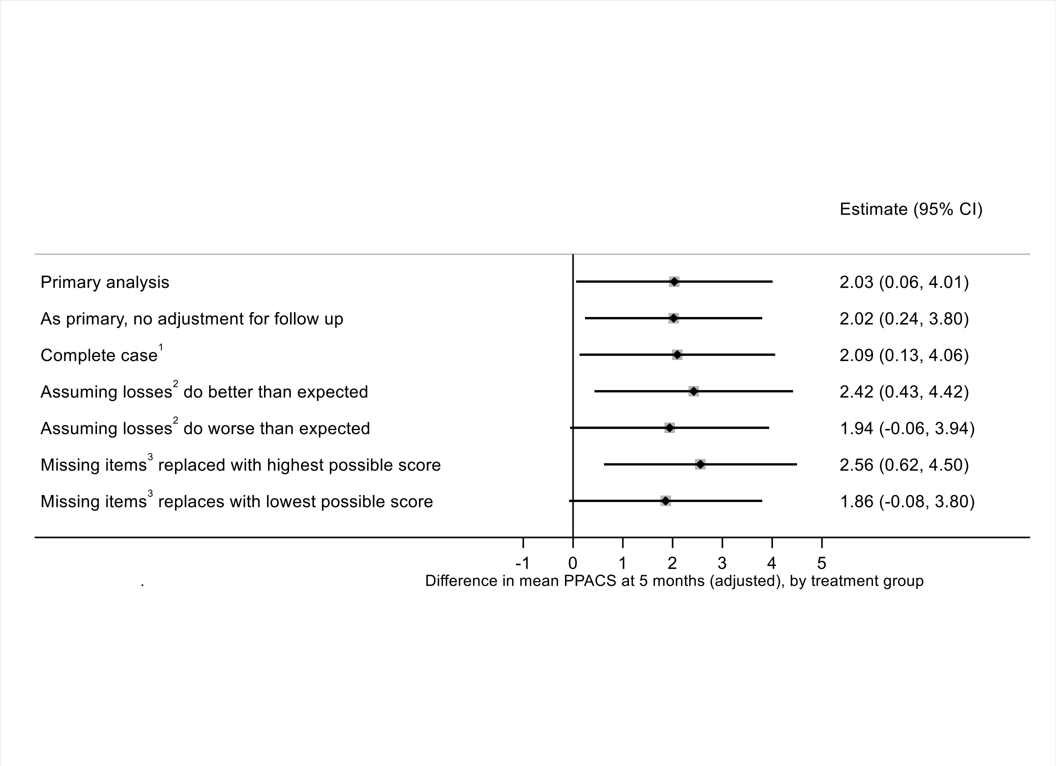

The primary analyses included multiple imputation for unrateable items within the PPACS scales; multiple imputation for PPACS total scores in those who did not complete their 5-month follow-up assessment; and adjustment for length of follow-up. Sensitivity analyses included repeating the primary analysis (as above) with no adjustment for length of follow-up. [1]Using multiple imputation [MI] solely for unrateable items on the PPACS scale and excluding those who did not complete their 5-month follow-up. [2]Assuming better and worse PPACS scores than predicted by multiple imputation by adding or subtracting (as appropriate) one standard deviation of the mean change in PPACS scores between baseline and 5-month follow-up assessment to the imputed value in those who did not complete their 5-month follow-up.[3] Unrateable PPACS items were replaced with the highest or lowest possible score for the item.

PPACS=Preschool Parental Account of Children’s Symptoms.

© 2021 O’Farrelly C et al. *JAMA Pediatrics*.

eFigure 2. Subgroup Analysis of the Primary Outcome (PPACS Total) by Age of Child, Number of Participating Caregivers, and Baseline (SDQ-Externalizing) Behavior

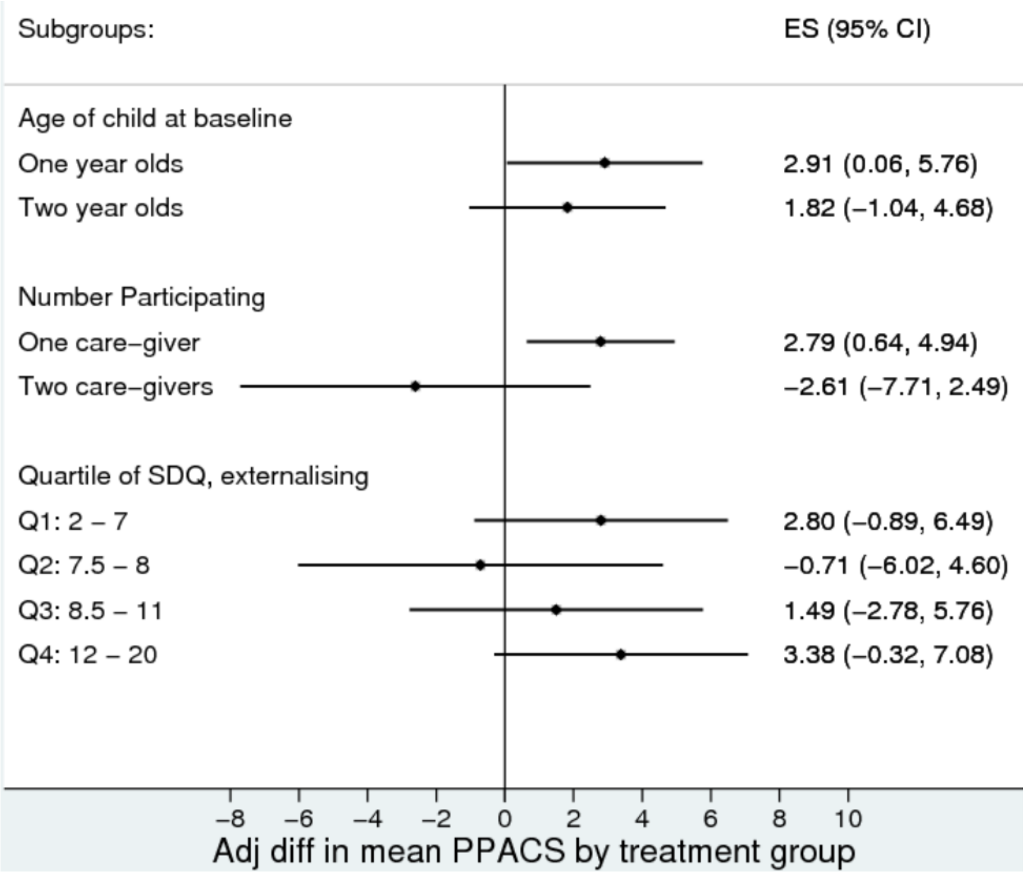

Data are mean (SD) or adjusted treatment difference (95% CI). All subgroup analysis were pre-specified except for SDQ externalising.

[a] Adj diff in mean is the difference between treatment groups from linear regression analysis of the outcome measure adjusted for the baseline score, treatment centre, randomised group, length of follow-up, age of child and number of parents/caregivers participating. Positive differences represent greater adjusted decreases in symptoms in the VIPP-SD rather than in the UC group.
SDQ=Strengths and Difficulties Questionnaire. PPACS=Preschool Parental Account of Children's Symptoms.

© 2021 O'Farrelly C et al. *JAMA Pediatrics*.
