# Supplementary material for: A Brief Home-Based Parenting Intervention to Reduce Behavior Problems in Young Children: A Pragmatic Randomized Clinical Trial
Source: JAMA Pediatr. 2021 Mar 15;175(6):1–10. doi: 10.1001/jamapediatrics.2020.6834 (PMC7961467; doi:10.1001/jamapediatrics.2020.6834)
Supplement: Supplement 3. — Data Sharing Statement [file jamapediatr-e206834-s003.pdf]

Data Sharing Statement

O'Farrelly. A Brief Home-Based Parenting Intervention to Reduce Behavior Problems in Young Children. *JAMA Pediatr*. Published March 15, 2021. doi:10.1001/jamapediatrics.2020.6834

Data
Data available: Yes
Data types: Deidentified participant data, Data dictionary
How to access data: Data will be available one year after publication from Paul Ramchandani - pr441@cam.ac.uk
When available: beginning date: 01-01-2022

Supporting Documents
Document types: None

Additional Information
Who can access the data: researchers whose proposed use of the data has been approved
Types of analyses: For a specified purpose
Mechanisms of data availability: After approval of a proposal with a signed dat access agreement
